# Interventions for maintenance of surgically induced remission in Crohn’s disease: a systematic review and network meta-analysis

**DOI:** 10.1136/bmjgast-2025-002086

**Published:** 2025-12-21

**Authors:** Morris Gordon, Shiyao Liu, Vassiliki Sinopoulou, Daniel Arruda Navarro Albuquerque, Gordon Moran

**Affiliations:** 1 University of Central Lancashire, Preston, England, UK; 2 School of Medicine and Dentistry, University of Central Lancashire, Preston, UK; 3 University of Nottingham, Nottingham, England, UK

**Keywords:** CROHN'S DISEASE, SURGERY FOR IBD, META-ANALYSIS, IBD CLINICAL

## Abstract

**Objectives:**

Approximately 50% of patients with Crohn’s disease (CD) undergo surgery, and a significant proportion suffer from a post-surgical recurrence. We conducted a network meta-analysis to compare the efficacy of various interventions.

**Design:**

Systematic review and network meta-analysis.

**Data sources:**

MEDLINE, EMBASE and Cochrane Library were searched from inception up to February 2025.

**Eligibility criteria:**

Randomised controlled trials (RCTs), reported in any language, comparing treatments used for maintaining surgically induced remission in CD were included. The primary outcomes were clinical relapse, endoscopic relapse and withdrawal due to adverse events.

**Data extraction and synthesis:**

Two reviewers independently extracted data and assessed risk of bias. Certainty of evidence was rated with GRADE (Grading of Recommendations Assessment, Development and Evaluation), and SUCRA (surface under the cumulative ranking curve) was used to rank treatments.

**Results:**

There were 34 RCTs (n=3197). For clinical relapse, adalimumab reduced the risk of relapse compared with placebo (moderate certainty), risk ratio (RR) 0.31 (95% CI 0.16 to 0.60), moderate effect size. Two treatments may reduce the risk of clinical relapse (low certainty): 5-aminosalicylic acid (RR 0.79, 95% CI 0.66 to 0.94; trivial effect size) and purine analogues (RR 0.79, 95% CI 0.66 to 0.96; trivial effect size). All other treatments were of very low certainty. For endoscopic relapse, vedolizumab probably reduced the risk of relapse (moderate certainty), RR 0.37 (95% CI 0.17 to 0.80), large effect size. Adalimumab may reduce the risk of endoscopic relapse (low certainty), RR 0.47 (95% CI 0.27 to 0.80), large effect size. All other treatments were of very low certainty.

**Conclusions:**

Adalimumab and vedolizumab reduce endoscopic relapse with moderate to large effects supported by moderate to low certainty evidence. Adalimumab also prevents clinical relapse with moderate certainty. Other therapies either had evidence of trivial effect size or very low certainty evidence. Postoperative maintenance should be individualised based on patient risk and treatment profile.

**WHAT IS ALREADY KNOWN ON THIS TOPIC**

- Post-surgical relapse is common in Crohn’s disease (CD).
- However, optimal maintenance therapy remains uncertain.
- This network meta-analysis compares the efficacy and safety of treatments in maintaining post-surgical remission.

**WHAT THIS STUDY ADDS**

- Adalimumab and vedolizumab reduced clinical and endoscopic relapse, respectively, with moderate certainty and large effect sizes.
- Other treatments had trivial effects or very low certainty.

**HOW THIS STUDY MIGHT AFFECT RESEARCH, PRACTICE OR POLICY**

- Moderate-certainty evidence supports use of effective advance therapies to prevent relapse after CD surgery.
- These findings may inform treatment selection and support shared decision-making, while recognising that recommendations are not one-size-fits-all and should be individualised.

## Introduction

 Crohn’s disease (CD) is a chronic, relapsing and remitting inflammatory disease of the gastrointestinal tract with a UK prevalence of 0.4%,^1 2^ a global prevalence of ~5 million cases, with the majority of countries experiencing an increase in the age-standardised prevalence rate, with newly industrialised nations showing a persistent increase in incidence.^3 4^

Improvements in early detection and advances in medical therapy have led to a reduction in surgery rates, with a 5-year cumulative risk of approximately 18%.^5^ However, the need for repeat surgery remains a significant clinical challenge. A recent meta-analysis reported that 17.7% of patients require a second resection within 5 years of their first surgery, increasing to 31.3% within 10 years.^5 6^ Referral-centre data show that about 50% relapse clinically within 5 years, and almost all have endoscopic relapse within 3 years after surgery.^2^

Conventional therapies, such as corticosteroids and immunomodulators, played a significant role in the management of CD before the development of advanced targeted therapies. Various classes of biologics, namely, anti-tumour necrosis factor (TNF) antibodies, anti-integrins, anti-IL-12/23p40, anti-IL23p19 and oral small molecules (JAKi) have been developed in the last two decades and many of them are now assimilated in world-wide inflammatory bowel disease (IBD) practice.^7 8^

With the availability of multiple treatment options, the choice of therapy to be used in a post-surgical setting can be challenging. Clinical and patient factors cause variable efficacy, complicating personalised decisions. The development of optimal treatment strategies has been identified as a top research priority for IBD by the UK priority-setting partnership with the James Lind Alliance.^9^

Given the different interventions reported recently and diverse outcomes, a comprehensive synthesis of evidence to evaluate the comparative effectiveness is essential. Network meta-analysis (NMA) offers a robust framework to compare all available data from both direct and indirect evaluations and rank them based on their relative effectiveness and safety. This systematic review and NMA aimed to evaluate the effects and potential harms of available interventions for maintaining surgically induced remission in CD and rank the treatments in order of effectiveness.

## Methods

### Study reporting and protocol registration

A protocol for this review was made publicly available prospectively through the University of Central Lancashire’s online repository.^10^

This systematic review is reported in accordance with PRISMA guidelines (see online supplemental file for checklist).

### Search strategy

The search strategy was developed in collaboration with an information specialist. We searched MEDLINE, EMBASE and Cochrane Library from inception to February 2025 (online supplementary file eAppendix 2).

### Study selection and inclusion criteria

Randomised controlled trials (RCTs) compared different interventions used for maintaining surgical induced remission in people with CD were included. Participants who have received maintenance treatment for at least 6 months were included. Studies that assessed dietary manipulation or herbal medicines were excluded. No restrictions were applied regarding language, publication date or publication status. Trials that were non-randomised, quasi-randomised or contained non-randomised components, such as induction phases, long-term follow-up periods or control groups lacking randomisation, were excluded. Additionally, studies comparing different dosages of the same intervention, top-down versus bottom-up treatment strategies, dose escalation protocols or monitoring of trough levels were not considered eligible.

Two reviewers independently screened the titles and abstracts to identify potentially eligible studies (SL and VS). Full-text articles of these studies were then retrieved by the same two reviewers. Any disagreements were resolved by discussion or by consulting the third review author (MG) if necessary. We contacted study authors for clarification regarding study eligibility where required.

### Study outcomes

The primary outcomes of this study were clinical relapse, endoscopic relapse and withdrawal due to adverse events (WAEs). Studies that reported either the proportion of participants who failed to maintain remission or the time to relapse were considered to provide the most relevant clinical and endoscopic relapse outcome measures. We accepted the authors’ definitions for clinical and endoscopic relapse. Secondary outcomes included total adverse events (TAEs) and serious adverse events (SAEs).

### Outcome thresholds

We used prospectively agreed on outcome thresholds for the assessment of imprecision of magnitude effects which were decided via an online Delphi survey of IBD stakeholders (clinicians, nurses, patients) (online supplemental file eTable 6).^11 12^ Pre-agreed thresholds are the recommended approach for assessing confidence in NMA and guidelines.^13^

### Data extraction and risk-of-bias assessment

Data extraction was performed independently by two review authors using a pre-designed data extraction form. The risk of bias for the included studies was independently evaluated by two reviewers using the Cochrane Risk of Bias (RoB) 1 tool.^14^ Discrepancies between reviewers were resolved through consensus and discussion with a senior author.

### Data synthesis and statistical analysis

We conducted a NMA using the frequentist model with the netmeta package in R to compare the interventions across studies.^15^ The network for the models was presented graphically through network diagrams, allowing assessment of the evidence available for the different comparisons. Any concurrent treatment lasting more than 6 months was incorporated into the intervention classification.

All review outcomes were dichotomous and were expressed in risk ratios (RRs) with corresponding 95% CIs. Missing dichotomous data were handled using a modified intention-to-treat (ITT) principle, assuming participants lost to follow-up were treatment failures. We assessed the assumption of transitivity by comparing the distribution of potential effect modifiers across the pairwise comparisons. Analyses were conducted for the data defined by the authors as primary endpoints or end of the randomised study data.

Heterogeneity was assessed statistically using the I^2^ statistic for each pairwise comparison, and with the loop-specific approach for the direct and indirect estimates. Surface under the cumulative ranking curve (SUCRA) was used to rank treatments, and placebo was used as the comparison treatment for all other treatments. Funnel plots were used to assess publication bias for pairwise analyses with at least 10 studies.

### Subgroup and sensitivity analyses

Methodological heterogeneity was explored through subgroup and sensitivity analyses. Studies were stratified into two subgroups based on the duration of follow-up: short-term (≤12 months) and long-term (>12 months). Several sensitivity analyses were conducted to assess the robustness of the primary outcomes. One analysis excluded studies that enrolled only high-risk patients (as defined by study). Another excluded studies that did not use the Crohn’s disease activity index (CDAI) to define clinical relapse. A third analysis excluded studies that included patients with clinical or endoscopic relapse at baseline. The fourth sensitivity analysis excluded studies in which antibiotics were used concurrently, regardless of the duration.

### GRADE assessment for the certainty of evidence

The quality of the studies was appraised, and the reviewers assigned a level of evidence to each study using the Grading of Recommendations Assessment, Development and Evaluation (GRADE) criteria with the approach focusing on NMA.^16 17^ The certainty of the direct and indirect evidence was assessed based on risk of bias, inconsistency, indirectness and publication bias. Subsequently, the certainty of the network evidence was evaluated for imprecision and incoherence, taking into account the percentage contribution of the direct and indirect evidence. The results were presented using ‘GRADEing Of Relative effect Diagram Of NMA’ (GORDON) plots to aid the interpretation and integration of efficacy, ranking, magnitude and certainty data.^18^

GRADE was used in combination with SUCRA to rank treatments. In the summary of findings tables, treatments are ranked from higher to lower SUCRA probability, and their corresponding GRADE certainty and estimates are presented. In the Abstract, Results section and graphical plot figures, treatments are presented from high to low GRADE certainty and ranked by SUCRA probability within their respective GRADE assessment rating (high, moderate or low).

## Results

**Figure 1:**
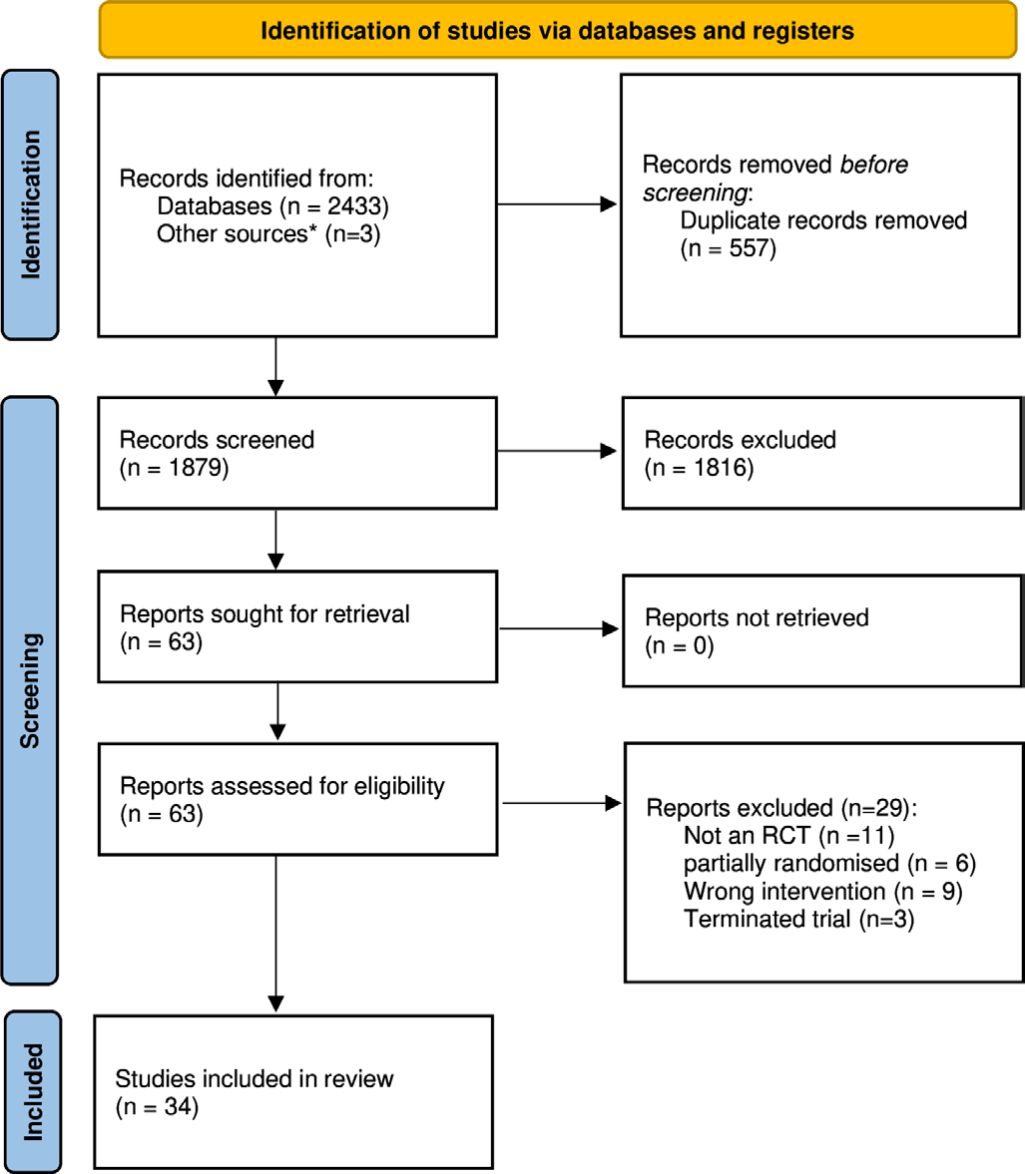
PRISMA (Preferred Reporting Items for Systematic Review and Meta-Analysis Protocols) flow diagram. RCT, randomised controlled trial.*Records identified by screening the reference lists of all included studies and relevant systematic reviews, as well as abstracts from major gastroenterology conferences.

A total of 2436 records were identified by the systematic search and its updates. Sixty-three records (associated records were merged) were assessed, and 29 were excluded with reasons which resulted in 34 included RCTs (n=3197) (online supplemental eTable 1 and 5). Inter-reviewer agreement was at least substantial (κ=0.72). The results of the search are presented in the PRISMA (Preferred Reporting Items for Systematic Review and Meta-Analysis Protocols) flow diagram (figure 1, online supplemental eAppendix 3 and 4). The risk of bias summary for the included studies is presented in online supplemental eFigure 4 and eAppendix 1.

Across the included studies, 20 reported the mean age of each group, which ranged from 30.5 to 40 years. In 13 studies, participant age was reported as a median. Thirty-one studies reported the gender distribution of each group with both male and female included. In 14 of these studies, each group had a higher proportion of male participants. Twenty studies reported the number of current smokers. Length of treatment if included studies ranged between 6 and 72 months. The sample sizes of included studies ranged between 20 and 324. The majority of the studies recruited participants within 3 months of surgery or before hospital discharge, except in Reinisch 2010, where participants were enrolled between 6 and 24 months post-surgery. The time since operation was not reported in Sutherland 1997.

Most studies enrolled patients in clinical and/or endoscopic remission at baseline, while seven included clinical relapse and two included endoscopic relapse. Five studies enrolled only high-risk patients (described by study authors using a range of definitions); the definitions were presented in online supplemental eTable 1. Most studies used CDAI as the criterion for clinical relapse, typically applying a threshold of 150 or 200. Eight studies adopted alternative criteria as indicators (online supplemental eTable 3). Eighteen studies defined endoscopic recurrence as Rutgeerts score ≥i2. One study each used thresholds of ≥i1, i2a or i2b, while three studies defined recurrence as ≥i3. One study used the Rutgeerts score but did not specify the threshold, and one study did not use the Rutgeerts score (online supplemental eTable 3). More details on dosages, time from surgery to recruitment, concurrent therapies, site of surgery, length of therapy and timepoint of outcome measurement of the studies can be found in online supplemental eTables 1 and 2.

**Figure 2:**
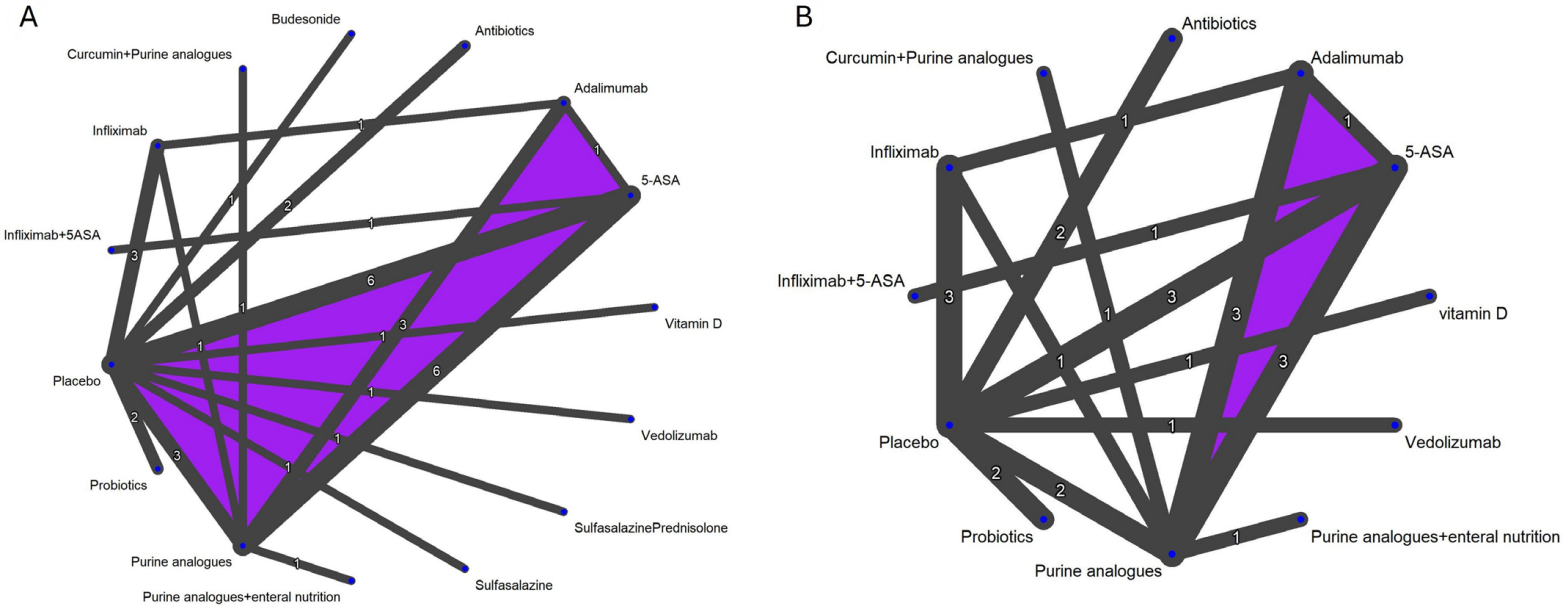
Network plots for (A) clinical relapse and (B) endoscopic relapse.

Summary of findings tables and GRADE decisions for all outcomes can be found in online supplemental eTable 7; network plots in online supplemental eFigure 1; network forest plots, SUCRA probabilities and direct/indirect/network estimates forest plots in online supplemental eFigure 2; and subgroup and sensitivity analyses in online supplemental eFigure 3. No pairwise comparisons included 10 or more studies; therefore, publication bias funnel plots were not generated. Network plots for clinical relapse and endoscopic relapse are shown in figure 2.

### Clinical relapse

**Figure 3:**
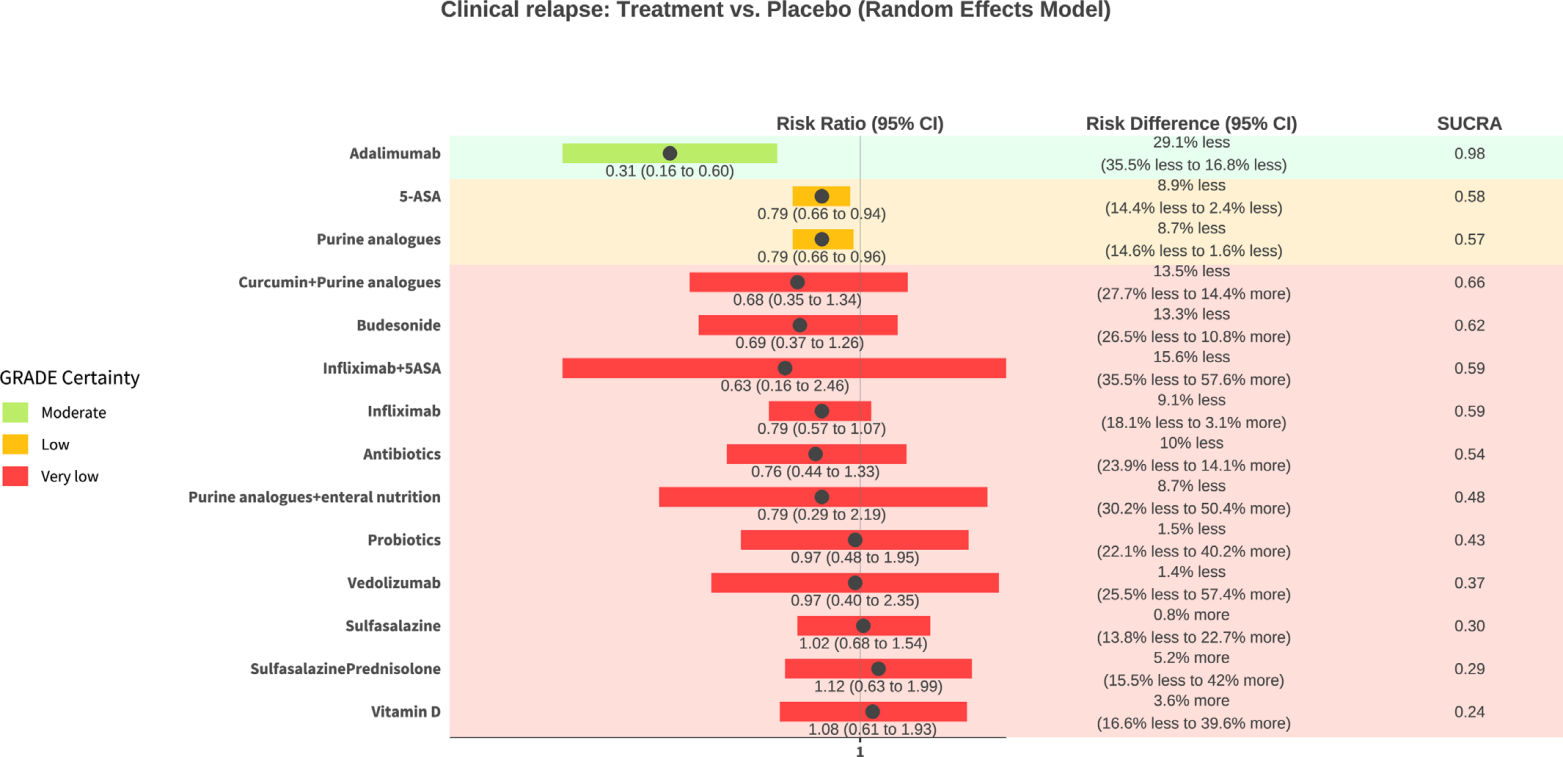
GORDON (GRADEing Of Relative effect Diagram Of NMA) plot of clinical relapse network results with placebo as comparison. GRADE, Grading of Recommendations Assessment, Development and Evaluation; NMA, network meta-analysis; SUCRA, surface under the cumulative ranking curve.

Thirty-two (n=3016) studies assessing 15 interventions (5-Aminosalicylic acid (5-ASA), adalimumab, antibiotics, budesonide, curcumin+purine analogues, infliximab, infliximab+5 ASA, probiotics, purine analogues, purine analogues+enteral nutrition, sulfasalazine, sulfasalazine+prednisolone, vedolizumab, vitamin D) were included in the clinical relapse NMA (online supplemental eTable 4, figure 2). Individual treatments were compared with placebo to further examine the results of the NMA. The risk of clinical relapse with placebo was 42.4% (range 12–95.5%). Network heterogeneity was 27.7% (I^2^). Table 1, figure 3 and online supplemental eFigures 1–3 provide a summary and graphical presentation for this outcome.

**Table 1:** Summary of findings for clinical relapse

Clinical relapse							
Patient or population: people with Crohn’s disease post-surgery							
Settings: hospital setting							
Intervention: advanced therapies/purine analogues/purine analogues+enteral nutrition/5-ASA/sulfasalazine/ antibiotics/probiotics/synbiotics/ curcumin+purine analogues/vitamin D/budesonide/sulfasalazine+prednisolone							
Comparison: placebo							
**Treatment (timepoint of outcome measurement)**	**Network evidence**		**Anticipated absolute effects for network estimate**			**Number needed to treat (NNT) (95% CI)**	**Notes**
	**RR (95% CI)**	**Certainty**	**Risk with placebo**	**Risk with agent****†** **(95% CI)**	**% risk difference with agent****‡** **(95% CI)**		
**Adalimumab (12–24 months)**	**0.31 (0.16 to 0.6)**	**Moderate**	**424 per 1000**	**131 per 1000 (68 to 254)**	**29.1% less** **(35.5% less to 16.8% less)**	**3 (3 to 6)**	**Probably moderate effect better than placebo (small to large)**
		**⊕⊕⊕⊖**					
Curcumin+purine analogues (6 months)	0.68 (0.35 to 1.34)	Very low	424 per 1000	288 per 1000 (148 to 568)	13.5% less (27.7% less to 14.4% more)	NA	The data are very uncertain
		⊕⊖⊖⊖					
Infliximab+5-ASA (36 months)	0.63 (0.16 to 2.46)	Very low	424 per 1000	267 per 1000 (68 to 1000)	15.6% less \(35.5% less to 57.6% more)	NA	The data are very uncertain
		⊕⊖⊖⊖					
Budesonide (12 months)	0.69 (0.37 to 1.26)	Very low	424 per 1000	293 per 1000 (157 to 534)	13.3% less (26.5% less to 10.8% more)	NA	The data are very uncertain
		⊕⊖⊖⊖					
Infliximab (12–24 months)	0.79 (0.57 to 1.07)	Very low	424 per 1000	335 per 1000 (242 to 454)	9.1% less (18.1% less to 3.1% more)	NA	The data are very uncertain
		⊕⊖⊖⊖					
**5-ASA (12–72 months)**	**0.79 (0.66 to 0.94)**	**Low**	**424 per 1,000**	**335 per 1000 (280 to 399)**	**8.9% less** **(14.4% less to 2.4% less)**	**11 (7 to 40)**	**Maybe trivial effect better than placebo (trivial to small)**
		**⊕⊕⊖⊖**					
**Purine analogues** **(6–36 months)**	**0.79 (0.66 to 0.96)**	**Low**	**424 per 1000**	**335 per 1,000 (280 to 407)**	**8.7% less** **(14.6% less to 1.6% less)**	**11 (7 to 59)**	**Maybe trivial effect better than placebo (trivial to small)**
		**⊕⊕⊖⊖**					
Antibiotics (6–2 months)	0.76 (0.44 to 1.33)	Very low	424 per 1000	322 per 1000 (187 to 564)	10% less (23.9% less to 14.1% more)	NA	The data are very uncertain
		⊕⊖⊖⊖					
Purine analogues+enteral nutrition (12 months)	0.79 (0.29 to 2.19)	Very low	424 per 1000	335 per 1000 (123 to 929)	8.7% less (30.2% less to 50.4% more)	NA	The data are very uncertain
		⊕⊖⊖⊖					
Vedolizumab (6 months)	0.97 (0.4 to 2.35)	Very low	424 per 1000	411 per 1,000 (170 to 996)	1.4% less (25.5% less to 57.4% more)	NA	The data are very uncertain
		⊕⊖⊖⊖					
Probiotics (6–12 months)	0.97 (0.48 to 1.95)	Very low	424 per 1000	411 per 1000 (204 to 827)	1.5% less (22.1% less to 40.2% more)	NA	The data are very uncertain
		⊕⊖⊖⊖					
Sulfasalazine (18 months)	1.02 (0.68 to 1.54)	Very low	424 per 1000	432 per 1000 (288 to 653)	0.8% more (13.8% less to 22.7% more)	NA	The data are very uncertain
		⊕⊖⊖⊖					
Vitamin D (6 months)	1.08 (0.61 to 1.93)	Very low	424 per 1000	458 per 1000 (259 to 818)	3.6% more (16.6% less to 39.6% more)	NA	The data are very uncertain
		⊕⊖⊖⊖					
Sulfasalazine+prednisolone (36 months)	1.12 (0.63 to 1.99)	Very low	424 per 1000	475 per 1000 (267 to 844)	5.2% more (15.5% less to 42% more)	NA	The data are very uncertain
		⊕⊖⊖⊖					

**GRADE Working Group grades of evidence**

**High certainty**: We are very confident that the true effect lies close to that of the estimate of the effect.

**Moderate certainty**: We are moderately confident in the effect estimate; the true effect is likely to be close to the estimate of the effect, but there is a possibility that it is substantially different.

**Low certainty**: Our confidence in the effect estimate is limited; the true effect may be substantially different from the estimate of the effect.

**Very low certainty**: We have very little confidence in the effect estimate; the true effect is likely to be substantially different from the estimate of effect.

* The risk with placebo has been calculated based on the cumulative placebo rates of all studies with a placebo arm.

† The risk with treatment has been calculated by multiplying the risk with control with the RR (95% CI). If the calculation results in more than 1000 per 1000 people, the number has been capped to 1000. Numbers have been rounded up to the closest whole number.

‡ The % risk difference has been calculated by subtracting the risk with control from the risk with treatment (95% CI) and dividing by 10. If the calculation results in more than 100% the number has been capped to 100%. Numbers have been rounded up to the closest whole number

GRADE, Grading of Recommendations Assessment, Development and Evaluation; RR, risk ratio.

None of the interventions were rated high for GRADE certainty. One treatment, adalimumab, was rated as moderate GRADE certainty evidence and probably reduces the risk of clinical relapse compared with placebo (RR 0.31, 95% CI 0.16 to 0.60, Number needed to treat (NNT)=3 (95% CI 3 to 6), moderate effect size magnitude). Two treatments were rated as low GRADE certainty evidence and may reduce the risk of clinical relapse compared with placebo. In order of SUCRA ranking, these are 5-ASA (RR 0.79, 95% CI 0.66 to 0.94, NNT=11 (95% CI 7 to 40), trivial effect size magnitude) and purine analogues (RR 0.79, 95% CI 0.66 to 0.96, NNT=11 (95% CI 7 to 59), trivial effect size magnitude). Eleven treatments had very low GRADE certainty and no conclusions can be drawn (online supplemental eTable 7).

Subgroup and sensitivity analysis results were presented in online supplemental eFigure 3. Subgroup analysis showed that in studies with a follow-up period of ≤12 months, only adalimumab (RR 0.28, 95% CI 0.10 to 0.77) demonstrated a statistically significant effect in preventing clinical relapse compared with placebo. In studies with a follow-up period of >12 months, both adalimumab (RR 0.24, 95% CI 0.08 to 0.69) and 5-ASA (RR 0.80, 95% CI 0.66 to 0.97) reduced the risk of clinical relapse compared with placebo. Sensitivity analyses revealed variations in treatment effects across different study populations. When excluding trials limited to high-risk patients, infliximab, adalimumab, 5-ASA and purine analogues remained effective. Excluding studies that did not employ CDAI definitions of relapse, adalimumab and antibiotics were superior to placebo. Finally, after removing studies with patients already experiencing clinical relapse at baseline, 5-ASA and purine analogues maintained their efficacy. No notable differences were observed in the sensitivity analysis excluding studies with concurrent antibiotic use, as compared with the main network analysis.

### Endoscopic relapse

**Figure 4:**
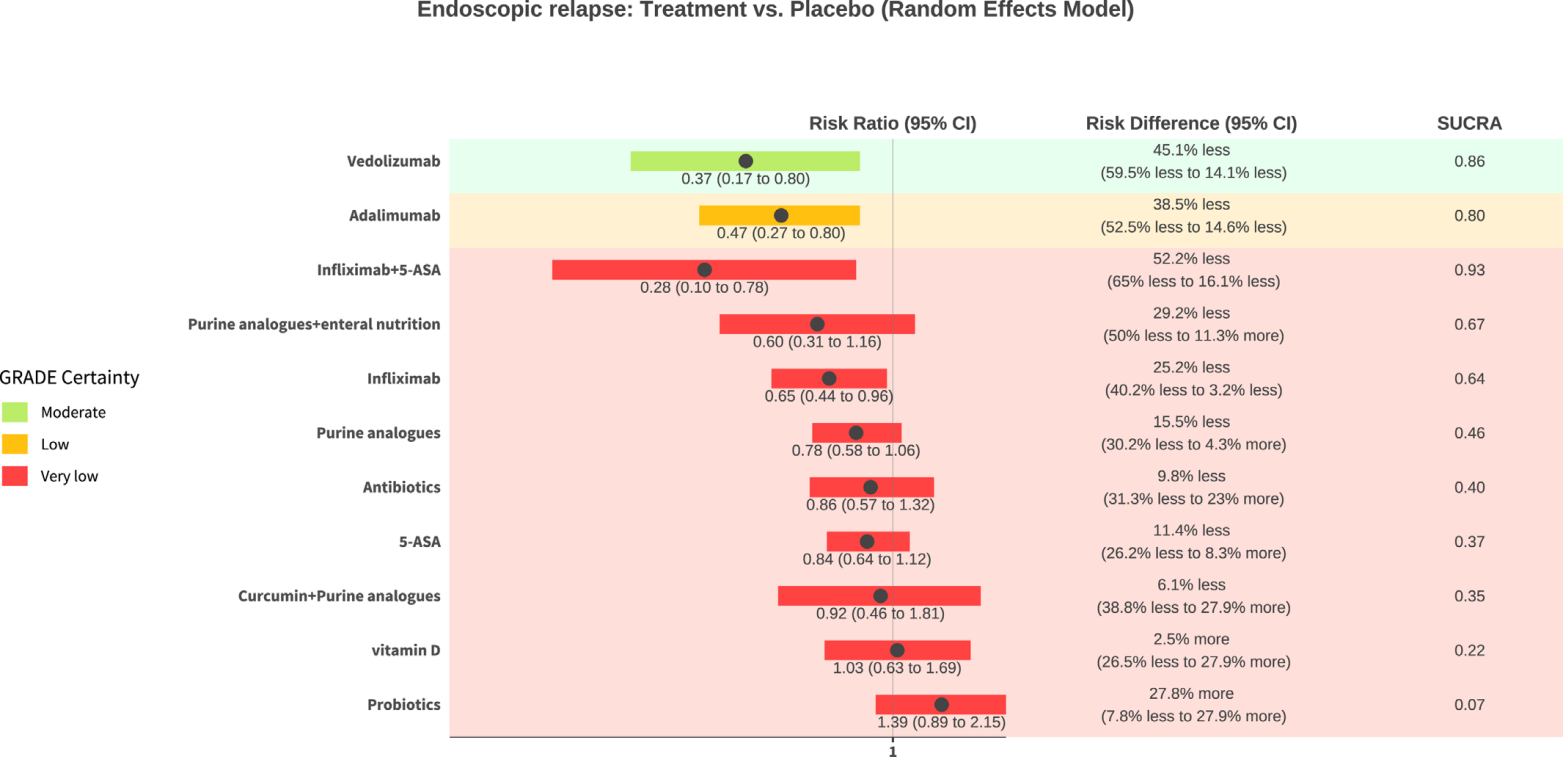
GORDON (GRADEing Of Relative effect Diagram Of NMA) plot of endoscopic relapse network results with placebo as comparison. GRADE, Grading of Recommendations Assessment, Development and Evaluation; NMA, network meta-analysis; SUCRA, surface under the cumulative ranking curve.

Twenty-four (n=2198) studies assessing 12 interventions (5-ASA, adalimumab, antibiotics, curcumin+purine analogues, infliximab, infliximab+5 ASA, probiotics, purine analogues, purine analogues+enteral nutrition, vedolizumab, vitamin D) were included in the endoscopic relapse network meta-analysis (online supplemental eTable 4, figure 2). The risk of endoscopic relapse with placebo was 72.1% (range 50–85.9%). Network heterogeneity was 61.5% (I^2^). Online supplemental eTable 7, figure 4 and online supplemental eFigures 1–3 provide a summary and graphical presentation of the results for this outcome.

None of the interventions were rated high for GRADE certainty. One treatment, vedolizumab, was rated as moderate GRADE certainty evidence and probably reduces the risk of endoscopic relapse compared with placebo (RR 0.37, 95% CI 0.17 to 0.8, NNT=2 (95% CI 2 to 7), large effect size magnitude). One treatment, adalimumab, was rated as low GRADE certainty evidence and may reduce the risk of endoscopic relapse compared with placebo (RR 0.47, 95% CI 0.27 to 0.8, NNT=3 (95% CI 2 to 7), large effect size magnitude). The results for nine of the treatments had very low GRADE certainty and no conclusions can be drawn (online supplemental eTable 7).

Subgroup and sensitivity analyses (≤12-month follow-up, excluding baseline endoscopic relapse, excluding concurrent antibiotic use) showed no considerable differences compared with the main network analysis. Among studies with a follow-up period of >12 months and in analyses excluding trials limited to high-risk patients, infliximab did not demonstrate a significant difference in preventing endoscopic relapse compared with placebo.

### Withdrawal due to adverse events

Twenty-five (n=2306) studies assessing 13 interventions (5-ASA, adalimumab, antibiotics, curcumin+purine analogues, infliximab, probiotics, purine analogues, purine analogues+enteral nutrition, sulfasalazine, synbiotics, vedolizumab, vitamin D) were included in the WAE network meta-analysis (online supplemental eTable 4). The risk of WAE with placebo was 13.7% (range 0–36.6%). Network heterogeneity was 32.8% (I^2^). Online supplemental eTable 7 and eFigures 1–3 provide a summary and graphical presentation of the results for this outcome.

The results for all of the treatments had very low GRADE certainty and no conclusions can be drawn (online supplemental eTable 7).

Visual inspection of the subgroup and sensitivity analysis results for studies’ follow-up period and excluding trials limited to high-risk patients did not reveal considerable differences compared with the main network analysis.

### SAEs and TAEs

Fourteen studies (n=1780) assessing 9 interventions for SAEs, and 22 studies (n=2128) assessing 10 interventions for TAEs (online supplemental eTable 4). Network heterogeneity was 5.5%, 0% and 21.3% respectively (I^2^). Online supplemental eTable 7 and eFigures 1–3 provide summaries of the results for these outcomes.

For both SAEs and TAEs, the results for all of the treatments had very low GRADE certainty, and no conclusions can be drawn (online supplemental eTable 7).

Visual inspection of the subgroup and sensitivity analysis results for studies follow-up period and excluding trials limited to high-risk patients did not reveal considerable differences compared with the main network analysis.

## Discussion

The management of CD has undergone advancements in recent years, with surgery continuing to be an effective treatment option. However, postoperative relapse remains a frequently reported challenge.^2 19 20^ Traditionally, immunomodulators such as AZA and 6-Mercaptopurine (6-MP) have been widely used; however, advanced therapies developed over recent decades may significantly improve relapse prevention, offering alternatives to traditional immunomodulators.^21^ Given the rapid evolution in pharmacotherapy, there is currently a gap in the availability of updated evidence synthesis summarising the latest evidence regarding the efficacy and safety of these interventions.

The NMA for the prevention of clinical relapse (32 studies) identified adalimumab as the most effective treatment strategy (NNT=3), with moderate certainty of evidence. The network for the prevention of endoscopic relapse (24 studies) identified vedolizumab (moderate certainty) and adalimumab (low certainty) as effective treatments (NNT=2 and 3, respectively). The combination of infliximab and 5-ASA, as well as infliximab alone, also demonstrated efficacy in preventing endoscopic relapse compared with placebo, although this finding had very low certainty of evidence and so no conclusions can be drawn. These findings are broadly consistent with previous reviews.^21 22^

These findings suggest that advanced therapies have a definite and primary role in preventing clinical relapse in the post-surgery setting. It is worth noting that the lack of evidence for infliximab should not be misinterpreted as evidence of no effect. Similarly, many therapies have not been studied and so no comments can be made. The postoperative management of CD should be individualised and personalised. The opportunity to initiate therapy after surgery must be balanced with the real risk of relapse post-surgery. It is suggested that an individual discussion is held with the patient to allow an informed decision which is mindful of patient-specific risk factors. This has been suggested in the recent UK IBD guidelines^23^ and both ECCO European guidelines^24^ and the AGA USA guidelines.^25^

Additionally, 5-ASA and purine analogues may be effective but demonstrated only trivial benefits over placebo. In the subgroup analysis by length of follow-up duration, adalimumab remained effective in both short-term and long-term follow-up groups, probably indicating the attenuated loss of response seen with this anti-TNF agent compared with, for instance, infliximab.^26 27^ 5-ASA demonstrated efficacy only in studies with treatment durations exceeding 12 months, but with the same trivial size of effect, essentially only effective in 9% of patients. Although the use of 5-ASA in CD remains controversial and is not recommended in current international guidelines,^28 29^ they are still commonly prescribed.^30^ Early disease recurrence may be driven by more aggressive disease activity, and 5-ASA has only weak anti-inflammatory properties so may not be an effective treatment strategy in the vast majority of patients with CD in the post-surgery state.^31^

Purine analogues also demonstrated a trivial benefit over placebo with low certainty of evidence. Despite the similarity of this result to 5-ASA, the reasons behind this and interpretation of the result are different. Cochrane evidence does highlight the discrepancy between the ITT and per protocol populations within the context of purine analogue use.^32^ Approximately one-third of patients withdrew due to adverse effects when receiving purine analogues. While primary randomised trials show some evidence of efficacy among patients who continued therapy,^18^ this effect is diminished in the meta-analysis and the current network meta-analysis due to high drop-out rates. In practice, this means there may be certain patients who are motivated to use purine analogues, such as those already on these therapies or perhaps with previous experience of them without side effects. This again can be discussed with patients as part of individual decision making. Although purine analogues and to a lesser extent 5-ASAs continue to be used in clinical practice, the evidence supporting their benefit is trivial in magnitude and of low or very low certainty.

It is worth noting that a significant limitation in assessing clinical relapse is the treatment duration in the included studies. Some immunosuppressants require a considerable period to achieve therapeutic effects. In the subgroup analysis of studies with treatment durations ranging from 13 to 72 months, more interventions demonstrated efficacy compared with placebo. Moreover, as follow-up duration extends, additional factors such as loss of response come into play, a variable that is of great significance especially in the TNF class. However, most RCTs included in this analysis had follow-up periods of within 1 year. While studies by Orlando *et al*^33^ and Rutgeerts *et al*^34^ followed patients for up to 10 and 3 years, respectively, the lack of randomisation limited their inclusion to end-of-treatment data within this NMA. Future trials with longer treatment durations are warranted to better evaluate the long-term effectiveness of interventions for preventing relapse.

There are issues with the completeness of the evidence that are worthy of consideration. It is clear that the evidence base for both purine analogues and 5-ASA is limited due to quality issues. This is disappointing given that both of these therapies are the most studied over many years with many patients. Having reviewed this topic many times before, we have had personal contact with many of the authors and maximised any opportunities to get additional information, so there is no scope for shifting this. Normally, this would lead to a call for further evidence, but given the magnitude that was apparent, this will be a challenge in terms of a recommendation. It is also apparent that for a number of the advanced therapies, there is a complete lack of evidence or, in the case of infliximab, limited strength of evidence. As such, and as has already been stated, this means that our conclusions are limited to those where there is research, and there may be several other therapeutic options, but currently, this is a gap in the evidence base. Another key gap is in terms of length of follow-up. Clearly, surgery is a major event and follow-up for prevention of relapse is vital. In particular, the endoscopic network involves studies that are mainly short, and the top performing therapy was only considered up to 6 months. This must be considered as a limitation in the applicability and completeness of the evidence.

Furthermore, heterogenous populations were studied with only a select cohort of studies recruiting patients with a high risk phenotype^35^,^39^ or with active disease at inclusion.^3840^,^45^ Another important potential confounder is the endoscopic definition of disease relapse. This is very variable in the included studies, ranging from Rutgeerts i2a to Rutgeerts i3. Prospective cohort studies did indeed show that a modified Rutgeerts score ≥i1 is associated with clinical recurrence, a modified Rutgeerts score of i1 or i2b is associated with endoscopic recurrence and a score ≥i2 b is associated with surgical recurrence. This granularity in disease activity measurements is not always reflected in the studies to date and possibly the endoscopic inclusions used may not be as clinically relevant to the most appropriate population.^46^ As is always a limitation in the endoscopic studies, central reading is a priority to limit reader bias, with a stable expert pool a must to ensure consistent results.^47^ Again, this standard methodology is not always reflected in the studies to date.

Finally, the vast majority of studies have used CDAI to define clinical relapse. CDAI is heavily weighted by subjectivity and the discordance between clinical symptoms and disease burden has been heavily documented in the last several years in the IBD literature.

There are also a number of key limitations within the methods of this review that must be considered when interpreting these findings. We have already commented on the use of the intention to treat principle. This is, for several reasons, an appropriate approach to take within reviews of this type. However, in the case of therapies with perhaps issues of tolerability, this can give a less than fully accurate view of the evidence. Previous cohort studies have indicated that discontinuation of purine analogue therapy due to intolerance may be as high as >30% in those of a female gender with the age of therapy initiation of >40 years of age particularly affected.^48^ This is not to suggest that a change of methods would be appropriate; rather, this must be considered as a limitation that is innate in this approach. It is also worth noting that NMA requires studies to be connected to the network, and as such, there are some of the therapies that simply could not be included due to not being compatible or not reporting the same outcomes. While they may be considered more fully within individual metro analysis in a network measure, there is the risk that they are not given due focus. However, we feel this risk is balanced given that most of them have been considered in individual meta-analyses in recent years, many of which our team has authored.

Future research is clearly needed, but we would suggest that this needs to take a number of important steps to be of best use to the field. Future studies need to include a more homogenous population, with a specific timeframe from the surgical intervention while using validated endoscopic scores and clinically relevant timelines.

## Conclusion

There is evidence that both adalimumab and vedolizumab are probably effective with a large magnitude of effect in preventing post-surgical relapse. There is a discrepancy between the efficacy of vedolizumab in the preoperative setting and our findings in this postoperative evidence synthesis. In moderate-to-severe CD, vedolizumab has trivial efficacy^49^ and is not suggested for the treatment of this condition.^23^ The heterogeneity of study populations, study design and definition of relapse might have accounted for the difference in findings. Moreover, postoperative CD has an inflammatory phenotype which is more responsive to treatment than a more complicated stricturing and penetrating phenotype very commonly observed in moderate to severe CD recruiting to clinical trials.^18 31^ Evidence for purine analogues and 5-ASA demonstrated they may be effective, but both with a trivial magnitude of effect. The use of purine analogues in this setting is heavily affected by compliance due to tolerability. The remaining therapies were of very low certainty and so no conclusions can be drawn. This analysis supports, and indeed this informs up-to-date guidelines in the UK and matches those in Europe and the USA, suggesting that advanced therapies most likely have a role in practice for high-risk patients moving forward. Conversely, at the moment, the mainstream use of alternative therapies in the post-surgical setting cannot be recommended.

## Supplementary material

10.1136/bmjgast-2025-002086 online supplemental file 1

## Data Availability

All data relevant to the study are included in the article or uploaded as supplemental information.
