# Supplementary material for: Interventions for maintenance of surgically induced remission in Crohn’s disease: a systematic review and network meta-analysis
Source: BMJ Open Gastroenterol. 2025 Dec 21;12(1):e002086. doi: 10.1136/bmjgast-2025-002086 (PMC12718597; doi:10.1136/bmjgast-2025-002086)
Supplement: online supplemental file 1 [file bmjgast-12-1-s001.docx]

Contents

[**eTable 1.** Characteristics of included studies (n=34) 2](#_Toc214381212)

[**eTable 2.** Surgery-related characteristics of included studies (n=34) 15](#_Toc214381213)

[**eTable 3.** Included studies’ efficacy outcome definitions and baseline disease activity (n=34) 21](#_Toc214381214)

[**eTable 4.** Outcomes data reported in the included studies. 24](#_Toc214381215)

[**eTable 5**. Excluded studies and reasons for exclusion. 27](#_Toc214381216)

[**eTable 6.** Predefined Magnitude Effect Thresholds. 29](#_Toc214381217)

[**eTables 7**. SUMMARY OF FINDINGS TABLES AND GRADE DECISIONS 30](#_Toc214381218)

[**eFigures 1**. Network plots 50](#_Toc214381219)

[**eFigures 2**. NETWORK FOREST PLOTS, SUCRA PROBABILITIES, AND DIRECT/INDIRECT/NETWORK ESTIMATES FOREST PLOTS 53](#_Toc214381220)

[**eFigures 3**. SUBGROUP AND SENSITIVITY ANALYSES 62](#_Toc214381221)

[**eFigure 4.** Risk of bias summary 68](#_Toc214381222)

[**eAppendix 1**. Supporting Judgements for Risk of Bias Assessment 69](#_Toc214381223)

[**eAppendix 2**. Search strategies 90](#_Toc214381224)

[**eAppendix 3**. References to studies included in this review 90](#_Toc214381225)

[**eAppendix 4**. References to studies excluded from this review 93](#_Toc214381226)

# **eTable 1.** Characteristics of included studies (n=34)

| **Study ID (Author, year)** | **Intervention**  **(dosage)**  **(numbers randomised)** | **Comparator**  **(dosage)**  **(numbers randomised)** | **Age, years** | **Sex per group**  **(M/F)** | **Smoker per group** | **Duration of disease, n** | **Concurrent therapies** | **Prior medication, n** | **Disease phenotype, n** | **Length of therapy** | **Timepoint when primary outcomes were measured** | **Studies including high-risk patients only** |
| --- | --- | --- | --- | --- | --- | --- | --- | --- | --- | --- | --- | --- |
| **Ardizzone 2004** | Azathioprine  (2 mg/kg/d) (n=71) | Mesalazine  (3 g/day)  (n=71) | Mean:  38.4 overall | IG: 45/26 CG: 50/26 | IG: 28/71 CG: 36/71 | IG: < 10 years 45, 10 years or more 26  IG: < 10 years 37, 10 years or more 34 | Concomitant use of the following drugs was not allowed: corticosteroids (with the exception of initial tapering), anti–tumor necrosis factor α, methotrexate, sulfasalazine, antibiotics (for a cumulative duration of more than 10 days), nonsteroidal anti-inflammatory drugs, and other aminosalicylates. Symptomatic treatment with antacids, anti-diarrheal agents, or spasmolytic agents was allowed but had to be scrupulously recorded for calculation of the CDAI score. | IG: Mesalamine or sulfasalazine 26, Corticosteroids 23, Immunosuppressants 6, None 16  CG: Mesalamine or sulfasalazine 36, Corticosteroids 18, Immunosuppressants 3, None 14 | Not reported | 24 months | 24 months (Patients were seen every 6 months.) | Not reported |
| **Armuzzi 2013** | Azathioprine  (2.5 mg/kg/d for 1 year)  (n=11) | Infliximab  (5 mg/kg/d at 0, 2, and 6 weeks and then every 8 weeks for 1 year) (n=11) | median (range): 32 (18 to 70) overall  IG: 32 (21−45)  CG: 34 (24−37) | IG: 7/4 CG: 8/3 | Not reported | median (IQR)  IG: 24 (12-54) months  CG: 24 (15-81) months | All patients also received oral metronidazole (500 mg bid) for 2 weeks after surgery. No other CD-related drugs were admitted during the study. | IG: infliximab 4, AZA 2  CG: infliximab 6, AZA 3 | Not reported | 12 months | 12 months | High-risk patients only. Definition: 2 or more than the following factors: young age at diagnosis (≤30 years), penetrating disease behavior, active smoking, perianal disease at diagnosis of CD, previous surgery and less than 3 years from previous surgery |
| **Bergman 1976** | Sulfasalazine  (Salazopyrin)  + prednisolone (n=57) | No treatment (n=40) | median: 28 overall | reported for those 84 who completed the study:  IG: 20/29 CG: 18/17 | Not reported | Not reported | Not reported | Not reported | Not reported | 8 months | Clinical relapse not reported; 33 weeks for secondary outcomes | Not reported |
| **Bommelaer 2020** | Curcumin  (3 g/d) +  azathioprine  (2-2.5 mg/kg/day)  (n=31) | placebo (n=31) | mean (SD):  IG: 35.0 (10.5)  CG: 37.6 (13.8)  Overall: 36.3 (12.0) | IG: 15/16  CG: 6/25 | IG: 7/31  CG: 13/31 | mean (SD):  IG: 7.9 (6.7) years  CG: 8.3 (9.7) years | No anti-TNF therapy | Not reported | IG: B1 inflammatory 7, B2 stricturing 12, B3 penetrating 11  CG: B1 inflammatory 4, B2 stricturing 16, B3 penetrating 21 | 6 months | 6 months (Patients were seen at 1, 3, 6 and 12 months after surgery.) | Not reported |
| **Brignola 1995** | Mesalazine  (3 g/d)  (n=44) | Placebo (n=43) | mean (SD):  IG: 39 (17)  CG: 34 (10) | IG: 22/22 CG: 20/23 | IG: 22/44 CG: 22/43 | IG: 75 (73) years  CG: 69 (54) years | Not reported | Not reported | Not reported | 12 months | 12 months | Not reported |
| **Caprilli 1994** | Mesalazine  (2.4 g/d)  (n=55) | No treatment (n=55) | mean (range):  IG: 35.5 (16 to 61)  CG: 33.7 (16 to 58) | IG: 32/15 CG: 23/25 | Not reported | mean (range):  IG: 5.0 (0-1 6) years  CG: 4.6 (0-1 7) years | No further medical treatment was given with the exception of antidiarrhoeal drugs. | IG: mesalazine 22, corticosteroids 28, metronidazole 12, sulphasalazine 9  CG: mesalazine 24, corticosteroids 31, metronidazole 12, sulphasalazine 12 | Not reported | Unclear | 24 months  (clinical and laboratory assessment: at 2 weeks after surgery, at 3, 6, and 12 months, and annually thereafter. Colon-ileoscopy: 6 and 12 months, and annually thereafter.) | Not reported |
| **Chermesh 2007** | Synbiotics 2000  (1 daily dose)  (n=20) | placebo (n=10) | mean (SD):  IG: 36.1 (13.0)  CG: 34.7 (9.9) | IG: 15/5 CG: 8/2 | IG: 8/20 CG: 2/10 | Not reported | Not reported | Not reported | IG: Noninflammatory 18, Inflammatory 2  CG: Noninflammatory 9, Inflammatory 1 | 24 months | 24 months (Patients were seen 0, 1, 2 and 3 months and every 3 months  thereafter till 24 months postsurgery) | Not reported |
| **De Bruyn 2021** | vitamin D  (weekly 25,000 IU)  (n=72) | placebo (n=71) | Median (IQR)  IG: 31 (25–46)  CG: 33 (25–46) | IG: 28/44  CG: 29/42 | IG: 13/70  CG: 13/67 | Not reported | All CD medication was stopped except for ongoing steroids, which were tapered gradually in the weeks after surgery according to local guidelines. No multivitamin or open-label vitamin D preparations were allowed during the study period and patients were not allowed to use tanning beds. | IG: Prednisone 45, Budesonide 35, Mesalamine 22, Azathioprine 47, 6-Mercaptopurine 13, Methotrexate 8, Infliximab 12, Adalimumab 13, Any anti-TNF 19  IG: Prednisone 43, Budesonide 34, Mesalamine 17, Azathioprine 40, 6-Mercaptopurine 20, Methotrexate 6, Infliximab 21, Adalimumab 11, Any anti-TNF 10 | IG: B1 inflammatory 11, B2 stricturing 40, B3 penetrating 21, P perianal disease 0  CG: B1 inflammatory 17, B2 stricturing 36, B3 penetrating 17, P perianal disease 0 | 6 months | 6 months (Patients were assessed at baseline and at weeks 2, 6, 12, and 26.) | Not reported |
| **D'Haens 2008** | Azathioprine  (100 to 150  mg/d)  (n=40) | placebo  (n=41) | mean:  IG: 38.8 (22 to 67)  CG: 40.0 (21 to 69 | IG: 24/16 CG: 20/21 | IG: 13/40 CG: 17/41 | Not reported | All patients received 3 months of metronidazole  therapy at a dose of 250 mg 3 times per day. Patients who could not tolerate metronidazole were switched to  ornidazole 500 mg twice per day orally. Antibiotics were allowed during the study for concurrent infections, but not for CD. Topical therapy for perianal CD could be continued if necessary. Cholestyramine was allowed for the treatment of bile acid diarrhea. | IG: Azathioprine 3, Steroid use at surgery 12  CG: Azathioprine 2, Steroid use at surgery 9 | Not reported | 12 months | 12 months (Patients were seen 2, 6, 12, 20, 28, 36, 44, and 52 weeks after randomization.) | 1 risk factor for the development of early/severe postoperative recurrence of their CD, based on the available literature: young age (<30 years); active smoking; corticosteroid use in the 3 months before surgery; surgery for the 2nd, 3rd, or 4th resection; and perforating disease, namely, abscess or fistula as an indication for surgery |
| **D'Haens 2025** | Vedolizumab (300 mg IV at week 0,8,16 and 24)  (n=43) | placebo (n=37) | median (range)  IG: 36 (19–79)  CG: 36 (18–74) | IG: 24/19  CG: 19/18 | Active smoking (more than  ten cigarettes per day)  IG: 5/43  CG: 8/37 | median (range)  IG: 9 (0–30) years  CG: 8 (0–45) years | All other preoperative medications were discontinued, except for loperamide, cholestyramine, and corticosteroids. | IG: anti-TNF 27, steroid use at baseline 3  CG: anti-TNF 23, steroid use at baseline 3 | Not reported | 6 months (24 weeks) | 6 months (26 weeks) | at least one risk factor for postoperative recurrence that included active smoking (more than ten cigarettes per day), perforating complications (abscess or fistula), previous exposure to TNF antagonists, or more than one previous resection |
| **Duan 2024** | Azathioprine + Exclusive enteral nutrition (AZA: 1 mg/kg/day for the first month, subsequently increased to 2–2.5 mg/kg /day) EEN: last for 3 months immediately following surgery followed by a normal diet) (n=42) | Azathioprine  (AZA: 1 mg/kg/day for the first month, subsequently increased to 2–2.5 mg/kg /day) (n=42) | Age at surgery for those 81 who completed the study: median [IQR]  IG: 33 [26–41]  CG: 36 [26–45] | for those 81 who completed the study:  IG: 29/12  CG: 30/10 | Smoking history  IG: 8/41  CG: 9/40 | median [IQR]  IG: 53 [26–83] months  CG: 31 [16–96] months | Naive to immunosuppressive or biological agents. Patients received remote medical care and follow-up regularly. Patients were educated and followed-up by telephone, WeChat app, and other digital tools. | IG: None 5, 5-Aminosalicylic acids 36  CG: None 6, 5-Aminosalicylic acids 34 | IG: Stricturing 24, Penetrating 17  CG: Stricturing 19, Penetrating 21 | 12 months | 12 months | one or more postoperative recurrence risk factors [active smoking, penetrating disease, history of intestinal resection, perianal lesions, patient age <30 years, resection of more than 50 cm of the intestine |
| **Ewe 1999** | Budesonide  (3 mg/d)  (n=40) | Placebo (n=40) | mean (SD):  IG: 35 (12)  CG: 33 (9) | IG: 21/22 CG: 16/24 | Not reported | mean (SD):  IG: 100 (74) months  CG: 81(58) months | No other drugs used in the treatment of Crohn’s disease were allowed. | Not reported | Not reported | 12 months | 12 months (Patients were seen at 6 weeks after surgery, at 3, 6, 9 and 1 2 months thereafter.) | Not reported |
| **Fukushima 2018** | Infliximab  (5 mg/kg at 0, 2, and 6 weeks, followed by every 8 weeks for 2 years)  (n=21) | No treatment (n=22) | mean (range):  IG: 36.6 (19 to 55)  CG: 37.6 (23 to 74) | IG: 17/4 CG: 13/9 | IG: 5/21 CG: 2/22 | mean (range):  IG: 5.5 (1–11) years  CG: 6.2 (1–11) years | The concomitant use of immune-modulators (e.g., azathioprine and 6-mercaptopurine) and immune-suppressants (e.g., cyclosporine and tacrolimus) was not allowed. | Prior infliximab  IG: 4  CG: 2 | IG: B1 inflammatory ,1 B2 stricturing 13, B3 penetrating 5  CG: B1 inflammatory 0, B2 stricturing 11, B3 penetrating 8, P perianal disease | 24 months | 24 months (Patients were seen at 6, 12,  18, and 24 months) | Not reported |
| **Hanauer 2004** | Mesalazine  (3 g/d)  (n=44) | 6-  mercaptopurine  (50 mg/d) (n=47)  Placebo  (n=40) | mean (SD):  IG1: 34.1 (10.9)  CG1: 34.9 (11.5)  CG2: 34.2 (10.9) | IG: 19/25 CG1: 23/24  CG2: 18/22 | Not reported | mean (SD):  IG1: 120 (105) months  CG1: 113 (94) months  CG2: 127 (100) months | No concurrent treatment for Crohn’s disease, aside from topical therapy for perianal disease, was allowed. Continuous use of nonsteroidal anti-inflammatory drugs was not allowed. | Not reported | Not reported | 24 months | 24 months (Patients were seen at week 7 after initiating treatment with the study drug and then at 3-month intervals for 24 months) | Not reported |
| **Hellers 1999** | Budesonide  (6 mg/d)  (n=63) | Placebo  (n=67) | mean (range): IG: 34 (20 to 76)  CG: 36 (17 to 81) | IG: 35/28 CG: 27/39 | Not reported | Not reported | Use of systemic glucocorticoids had to be discontinued within 30 days of surgery. No other concurrent medication for the treatment of CD, such as sulfasalazine, olsalazine, mesalamine, 4-aminosalicylic acid, metronidazole, immunosuppressive agents, or tuberculostatic agents, was permitted. Antibiotics were allowed in the immediate postoperative period but had to be discontinued before the study treatment was started. Antidiarrheals, such as loperamide and other opiates, were allowed. | Not reported | Not reported | 12 months | 12 months  (Patients were seen after 4 weeks (63 days) and after 13, 26, 39, and 52 weeks of treatment (61 week)) | Not reported |
| **Herfarth 2006** | 5-ASA  (4g/d)  (n=37) | Azathioprine  (2.0 to 2.5 mg/kg body weight/d)  (n=42) | Not reported | Not reported | Not reported | Not reported | Not reported | Not reported | Not reported | 12 months | 12 months | Not reported |
| **Herfarth 2013** | Ciprofloxacin  (1 g/d)  (n=17) | Placebo (n=16) | median (range):  IG: 33 (19 to 70)  CG: 27 (18 to 61) | IG: 10/7 CG: 8/8 | IG: 4/17 CG: 0/16 | median (range):  IG: 10 (0 -51) years  CG: 6 (0-25) years | No other treatments for CD or therapies involving more than 10 days of broad-spectrum antibiotics were permitted | IG: Mesalamine 4, Immunosuppression (azathioprine/6-MP or anti-TNF agent) 3, Steroids (Systemic steroids or budesonide) 7  CG: Mesalamine 5, Immunosuppression (azathioprine/6-MP or anti-TNF agent) 5, Steroids (Systemic steroids or budesonide) 4 | IG: Non-stricturing, non-penetrating 4, stricturing 8, penetrating 5 perianal disease  CG: Non-stricturing, non-penetrating 2, stricturing 10, penetrating 4 | 6 months | 6 months (Patients were seen at weeks 4, 12 and 24 and called at weeks 8, 18 and 28 after the start of medication.) | Not reported |
| **Hirsch 2023** | 6-mercaptopurine (Starting dose: 50 mg/day; escalate every 1–2 weeks; target: 1–1.5 mg/kg)  (n = 16) | adalimumab （week 0-160 mg; week 2-80 mg; week 4-40 mg. 40mg every other week）  (n = 19) | Mean (SE)  IG: 31.3 (1.9)  CG: 33.1 (2.1) | IG: 11/5  CG: 14/5 | Current smoker  IG: 5/16  CG: 2/19 | Mean (SD)  IG: 2.5 (6.7) years  CG: 5 (5.7) years | Patients experienced prior nonresponse or intolerance to TNFi agents were excluded. | IG: 6-MP 7, anti-TNF 2  IG: 6-MP 4, anti-TNF 7 |  | Unclear | 13 months  (Patients were seen at week 32 and week 58.) | Not reported |
| **Lochs 2000** | Mesalazine  (4 g/d)  (n=154) | Placebo  (n=170) | mean (SD):  IG: 33.5 (10.0)  CG: 33.8 (10.2) | IG: 71/81 CG: 85/81 | Not reported | Mean (SD)  IG: 6.5 (6.4) years  CG: 6.9 (6.4) years | Concomitant medication with the following drugs was disallowed during the trial: glucocorticoids with the exception of initial tapering, nonsteroidal anti-inflammatory drugs, immunosuppressive drugs, metronidazole, methotrexate, sulfasalazine, and other 5-aminosalicylates. Symptomatic treatment with antidiarrheal, antacid, or spasmolytic medication was allowed but had to be thoroughly documented. | Not reported | Not reported | 18 months | 18 months (Patients were seen at 6 weeks, 3 months, and every 3 months thereafter until 18 months.) | Not reported |
| **Lopez Sanroman 2017** | Azathioprine  (2.5 mg/kg/d)  (n=39) | Adalimumab (160 mg subcutaneously, then 80 mg at Week 2, or 40 mg at Week 4 and every 2 weeks)  (n=45) | median (interquartile range):  IG: 37.00 (31.00 to 47.00)  CG: 35.00 (30.0 to 40.0) | IG: 23/16 CG: 19/26 | IG: 9/39  CG: 11/45 | IG: 7.31 years  CG: 8.11 years | metronidazole 250 mg three times a day by mouth was added for the first 3 months. | IG: Glucocorticoids 38, Immunosuppressants [thiopurines or methotrexate] 28, anti- TNFα 21  CG: Glucocorticoids 42, Immunosuppressants [thiopurines or methotrexate] 35, anti- TNFα 28 | IG: B3 penetrating 11, P perianal disease 8  CG: B3 penetrating 20, P perianal disease 4 | Unclear | 12 months  (Patients were seen at week 24 and week 52) | Not reported |
| **Marteau 2006** | *Lactobacillus*  *johnsonii* LA1  (52218 CFU of lyophilised LA1 /d)  (n=48) | Placebo  (n=50) | median (interquartile range):  IG: 32 (27 to 42)  CG: 29 (27 to 34) | IG: 26/22 CG: 29/21 | Not reported | median (interquartile range):  IG: 33 (4–99) months  CG: 24 (7–88) months | Concomitant medication with the following drugs was not allowed: antibiotics for more than 15 days; aminosalicylates; glucocorticoids (after gradual withdrawal); non steroidal anti-inflammatory drugs; immunosuppressive drugs; anti-tumour necrosis factor agents; thalidomide; and other probiotics. Loperamide and cholestyramine were allowed | IG: Steroid 19  cG: Steroid 21 | IG: Penetrating 20  CG: Penetrating 25 | 6 months | 6 months | Not reported |
| **McLeod 1995** | Mesalazine  (3 g/d)  (n=88) | Placebo  (n=81) | mean (SD):  IG: 38.9 (13.1)  CG: 38.9 (13.2) | IG: 49/38 CG: 49/27 | Not reported | Not reported | Patients who were taking prednisone, sulfasalazine, metronidazole, or imuran and these drugs could not be discontinued were excluded. | Not reported | Not reported | 72 months | 72 months maximum (Patients were followed up every 3 months.) | Not reported |
| **Mowat 2016** | Mercaptopurine  (1 mg/kg/d) (n=128) | Placebo  (n=112) | mean (SD):  IG: 39.2 (12.08)  CG: 38.21 (13.4) | IG: 49/79 CG: 45/67 | Not reported | mean (SD):  IG: 7.7 (9.7)  CG: 7.6 (9.5) | Before randomisation, postoperative infections were treated and existing treatments for Crohn's disease stopped. | IG: Mercaptopurine 14, Azathioprine 80, Either thiopurine 81, Infliximab 21, Methotrexate 8, Other corticosteroids 97, Any immunosuppressants 112  IG: Mercaptopurine 5, Azathioprine 47, Either thiopurine 50, Infliximab 15, Methotrexate 7, Other corticosteroids 79, Any immunosuppressants 86 | Not reported | 36 months | 36 months  (Patients were seen weekly for the first 6 weeks and thereafter at 6-weekly intervals.) | Not reported |
| **Orlando 2020** | Azathioprine (2.0-2.5 mg/kg/day) (n=22) | Mesalazine (4 gr/die/day) (n=24) | Age at surgery, median [IQR]  IG: 38.0 [29.3, 44.8]  CG: 34.5 [27.8, 51.3] | IG: 14/8  CG: 16/8 | Current smoker  IG: 10/12  CG: 10/14 | Not reported | Not reported | Not reported | IG: structuring 20, fistulizing 2  IG: structuring 17, fistulizing 7 | 12 months | 12 months (Patients were followed-up at 15, 30 and 90  days, and then, every 3 months until 12 months and 10 years after randomization) | Not reported |
| **Prantera 2002** | LGG probiotic  (2.46 g/d; 6 billion CFU twice daily)  (n=23) | Placebo (n=22) | mean (range):  IG: 37.3 (22 to 71)  CG: 36.2 (22 to 64) | IG: 14/9 CG: 15/7 | IG: 10/23  CG: 6/22 | mean (range)  IG: 6.5 (0.6–21)  CG: 7.4 (1–19) | Antidiarrhoeals such as loperamide or other opiates, and  colestiramine, were allowed provided their use had been  calculated in the Crohn’s disease activity index (CDAI). | Not reported | Not reported | 12 months | 12 months (Patients were seen after 13, 26, 39, and 52 weeks of treatment.) | Not reported |
| **Regueiro 2009** | Infliximab (n=11) | Placebo (n=13) | median: IG: 43  CG: 32 | IG: 6/5 CG: 10/3 | IG: 5/11  CG: 1/11 | IG: 1.19 years  CG: 2.12 yeaars | Nearly half of the patients (four infliximab, seven placebo) were on an immunomodulator at time of surgery and continued these medications throughout the duration of the study. Seven patients (four infliximab, three placebo) were taking corticosteroids at the time of surgery, and all were weaned off completely by 2 weeks postoperatively. | Prior infliximab  IG: 3  CG: 5 | IG: B2 stricturing 0, B3 fistula 11  IG: B2 stricturing 2, B3 fistula 11 | 12 months | 12 months (Patients were seen at weeks 0, 2, 6, 14, 22, 30, 38, 46, 54, 56 and 60.) | Not reported |
| **Regueiro 2016** | Infliximab  (5 mg/kg q8w) (n=147) | placebo  (n=150) | mean (SD):  IG: 37.11 (3.49)  CG: 35.4 (12.41) | IG: 77/70 CG: 81/69 | Not reported | Mean (SD)  IG: 8.38 (8.651)  CG: 6.39 (7.457) | Patients receiving oral aminosalicylates or immunosuppressives (azathioprine [AZA], 6-mercaptopurine [6-MP], or methotrexate [MTX]) pre-surgery could continue treatment with maintenance of stable doses after resection. Patients not receiving these agents pre- surgery could not receive them post-surgery. Rectal aminosalicylates were discontinued at least 2 weeks before randomization. Initiation of corticosteroids or antibiotics for CD treatment was prohibited. | IG: Any CD medication 136, Anti-tumor necrosis factor 37 (Adalimumab 21, Infliximab 18, Certolizumab 3), Corticosteroid (excluding budesonide) 104, Budesonide 63, Immunosuppressive drugs 85 (6-MP 19, AZA 73, Methotrexate 11), Aminosalicylates 100, Antibiotics 94  CG: Any CD medication 144, Anti-tumor necrosis factor 30 (Adalimumab 17, Infliximab 15, Certolizumab 0), Corticosteroid (excluding budesonide) 96, Budesonide 67, Immunosuppressive drugs 88 (6-MP 22, AZA 77, Methotrexate 7), Aminosalicylates 101, Antibiotics 88 | Not reported | 24 months | 18 months (Patients were also seen prior to or at week 104 for the safety outcome.) | at least one of the following risk factors for disease recurrence: 1) qualifying surgery that was their second intra- abdominal resection within 10 years, 2) third or more intra-abdominal resection, 3) resection for a penetrating CD complication (e.g., abscess or fistula), 4) a history of perianal fistulizing CD provided the event had not occurred within 3 months, or 5) smoking 10 or more cigarettes per day for the past year. |
| **Reinisch 2010** | Mesalazine  (4 g/d) +  placebo  azathioprine (n=41) | Azathioprine  (2.0 to 2.5 mg/  kg/d) + placebo mesalazine (n=37) | Mean (SD):  IG: 35.5 (13.6)  CG: 36.0 (10.7) | IG: 24/17 CG: 20/17 | IG: 17/41  CG: 20/37 | Not reported | Medications prohibited during the study: immunosuppressants other than study drug, allopurinol, oxipurinol, or thiopurinol, AZA-containing or MES-containing drugs other than study drug, anti-TNFa therapy, oral antibiotics for > 4 weeks or more than 3 cycles of 2weeks, NSAIDs for > 2 weeks, corticosteroids, and cimetidine. | IG: Mesalazine 26, Sulfasalazine 1, Budesonide 13, Corticosteroids 16, Azathioprine 8, Infliximab 1, Other 6  IG: Mesalazine 28, Sulfasalazine 4, Budesonide 9, Corticosteroids 23, Azathioprine 6, Infliximab 2, Other 6 | IG: Non-stricturing/non-penetrating 6, Stricturing 15, Penetrating 16  CG: Non-stricturing/non-penetrating 4, Stricturing 10, Penetrating 27 | 12 months | 12 months (Patients were seen or samples were collected at baseline and weeks 2, 4, 8, 12, 18, 24, 30 and 36, 42, 48 with the final study visit at week 52.) | Not reported |
| **Rutgeerts 2005** | Ornidazole  (1 g/d)  (n=40) | Placebo  (n=40) | median (range): IG: 35 (26 to 44)  CG: 30.5 (24 to 41.25) | IG: 16/22 CG: 20/20 | IG: 17/40  CG: 19/40 | Median (IQR)  IG: 7 (4–11.75) years  CG: 3 (0.5–7.25) years | All other Crohn’s disease–related drugs were discontinued at the time of surgery except for glucocorticosteroids. Steroids were tapered after inclusion and were stopped within 1 month of inclusion. | IG: Immunosuppression 7, Steroids 20  CG: Immunosuppression 9, Steroids 14 | IG: inflammatory 4, Stenosing 16, penetrating 18  CG: inflammatory 5, Stenosing 14, penetrating 21 | 12 months | 12 months (Patients were seen at 3, 12, 24, 36 months.) | Not reported |
| **Savarino 2013** | Mesalazine  (3g/d)  (n=18) | Adalimumab  (160 to 80 mg  0 to 2 weeks  and 40 mg  thereafter) (n=16)  Azathioprine  (2 mg/kg/d) (n=17) | mean (range):  IG: 49 (24 to 69)  CG1: 45 (22 to 66)  CG2: 46 (25 to 65) vs | IG: 8/10 CG1: 8/8  CG2: 9/8 | IG: 6/18  CG1: 9/16  CG2: 4/1 | Mean (range)  IG: 6.9 (1– 18) years  CG1: 8.4 (1– 17) years  CG2: 7.9 (1– 17) years | Participants on antibiotics or immunomodulators at entry into the study discontinued these medications 12 weeks before surgery. Continuous use of NSAIDs was not allowed during the study. No other medications were prescribed except for occasional tablets of paracetamol or tramadol. | Prior infliximab  IG: 1  CG1: 5  CG2: 4 | IG: B2 stricturing 4, B3 fistula 14  CG1: B2 stricturing 4, B3 fistula 12  CG2: B2 stricturing 5, B3 fistula 12 | 24 months | 24 months (Patients were seen weekly for the first 4 weeks and then every 2 months.) | Not reported |
| **Sutherland 1997** | Mesalazine  (3 g/d)  (n=31) | Placebo (n=35) | Unclear | Unclear | Not reported | Not reported | No steroid, other mesalazine preparations, aspirin or other NSAIDs, immunosuppressives, narcotics except codeine or loperamide, antibiotics for longer than 14 days. | Not reported | Not reported | 11 months | 11 months (Assessments were required at 4, 12, 24, 36, 48 weeks.) | Not reported |
| **Tursi 2014** | Infliximab  (5 mg/kg at 0,  2, and thereafter 8-week  intervals)  (n=10) | Adalimumab  (160-80-40  mg/2-week  intervals)  (n=10) | mean (range): IG: 30.5 (20 to 33)  CG: 34.5 (22 to 39) | IG: 5/5 CG: 4/6 | IG: 3/10  CG: 2/10 | median (range)  IG: 48 (6–130) months  CG: 48 (6–144) months | All patients also received oral metronidazole (500 mg bid) for 2 weeks after surgery. No other CD-related drugs were admitted during the study. | IG: Mesalazine 5, azathioprine 2, infliximab 5  CG: Mesalazine 5, azathioprine 3, infliximab 4 | Not reported | 12 months | 12 months (Patients were contacted monthly.) | Not reported |
| **Wenckert 1978** | Sulfasalazine  (3 g/d)  (n=32) | Placebo  (n=34) | median: 24.5 overall | 33/33 overall | Not reported | Not reported | Other specific treatment was avoided. | Not reported | Not reported | 18 months | 18 months (after 1 month, 3 months, 6 months, etc., every third month. Relapse-free patients were observed for 24 months.) | Not reported |
| **Yoshida 2012** | Infliximab  (5 mg/kg at  8-week intervals) + Mesalamine  (1500 mg/day)  (n=15) | Mesalamine  (1500 mg/day)  (n=16) | mean (SE):  IG: 36.9 (11.6)  CG: 32.8 (10.2) | IG: 11/4 CG: 12/4 | IG: 3/15  CG: 3/16 | mean (SE):  IG: 11.6 (8.8) years  CG: 9.2 (7.1) years | If a patient was to receive AZA, 6- MP, PSL or increase the dosage of an ongoing medication, withdrawal from the trial was considered. | IG: ED ≤ 1200 kcal 11, Mesalamine 15, Corticosteroids 2, Immunomodulators 3, Infliximab 0  CG: ED ≤ 1200 kcal 7, Mesalamine 16, Corticosteroids 1, Immunomodulators 2, Infliximab 1 | Not reported | 36 months | 12 and 36 months. | Not reported |

# **eTable 2.** Surgery-related characteristics of included studies (n=34)

| **Study ID (Author, year)** | **Intervention**  **(dosage)**  **(numbers randomised)** | **Comparator**  **(dosage)**  **(numbers randomised)** | **Site of surgery**  **/ *exclusions** | **Indication for surgery, n** | **Type of surgery, n** | **Previous surgery, n** | **Time from surgery to recruitment** |
| --- | --- | --- | --- | --- | --- | --- | --- |
| **Ardizzone 2004^1^** | Azathioprine  (2 mg/kg/d) (n=71) | Mesalazine  (3 g/day)  (n=71) | Small bowel only 25.3; colon 5.6; small  bowel and colon 9.8; upper gastrointestinal tract 16.2  *Surgical procedures other than conservative surgery or for perianal disease only | Not reported | IG: Stricturoplasty 18, Minimal bowel resection 36, Minimal bowel resection stricturoplasty 17  CG: Stricturoplasty 18, Minimal bowel resection 34, Minimal bowel resection stricturoplasty 19 | Previous surgery  IG: 38  CG: 31 | < 2 weeks |
| **Armuzzi 2013^2^** | Azathioprine  (2.5 mg/kg/d for 1 year)  (n=11) | Infliximab  (5 mg/kg/d at 0, 2, and 6 weeks and then every 8 weeks for 1 year)  (n=11) | Not reported  *Active perianal disease, presence of  stoma | Not reported | Not reported | Previous surgery  IG: 4  CG: 4 | 2 to 4 weeks |
| **Bergman 1976^3^** | Sulfasalazine  (Salazopyrin)  + prednisolone  (n=57) | No treatment  (n=40) | Not reported | Not reported | Not reported | Not reported | Not reported |
| **Bommelaer 2020^4^** | Curcumin  (3 g/d) +  azathioprine  (2-2.5 mg/kg/day)  (n=31) | placebo  (n=31) | Ileal 27; colonic 2; ileocolonic 33  *Not reported | Not reported | Not reported | Prior bowel resection  IG: 14  CG: 14 | < 15 days |
| **Brignola 1995^5^** | Mesalazine  (3 g/d)  (n=44) | Placebo  (n=43) | Ileum 56, ileocaecal 46  *Surgery other than in ileal or ileocaecal  region | Not reported | Not reported | Not reported | ≤1 months |
| **Caprilli 1994^6^** | Mesalazine  (2.4 g/d)  (n=55) | No treatment  (n=55) | Not reported  *Disease localisation to the jejunum,  proximal ileum, leN colon, or ano-rectum | IG: occlusion 19, perforation 3, abscess 7, fistula 14, intractability 6,  recurring sub-occlusion 17,  others 1  IG: occlusion 19, perforation 2, abscess 9, fistula 11, intractability 10,  recurring sub-occlusion 21,  others 2 | IG: elective 35, emergency 12  CG: elective 36, emergency 12 | Not reported | 2 weeks |
| **Chermesh 2007^7^** | Synbiotics 2000  (1 daily dose)  (n=20) | placebo  (n=10) | Not reported  *Not reported | Not reported | Not reported | Previous surgery  IG: 4  CG: 2 | As soon as  participants  resume oral  intake after  surgery |
| **De Bruyn 2021^8^** | vitamin D  (weekly 25,000 IU)  (n=72) | placebo  (n=71) | Ileum only 67; colon only 3; ileo-colonic 72  * Macroscopic evidence for CD at the proximal or distal resection margin; ileorectal anastomosis or active perianal fistulae; patients with an exten-sive small-bowel resection, additional stricturoplasty or other small- bowel resections; patients with stoma | Not reported | Not reported | Previous surgery  IG: 17  CG: 14 | < 2 weeks |
| **D'Haens 2008^9^** | Azathioprine  (100 to 150  mg/d)  (n=40) | placebo  (n=41) | Perforating disease 48  *Macroscopic evidence for CD proximally or distally to the site of resection or the  presence of frank pancolitis or an ileorectal anastomosis, participants with a  stoma; operation for fibrostenosis only | Not reported | Not reported | 1st/2nd/3rd surgery  IG: 24/12/2  CG: 32/8/1 | 2 weeks |
| **D'Haens 2025^10^** | Vedolizumab (300 mg IV at week 0,8,16 and 24)  (n=43) | placebo  (n=37) | Not reported  *Not reported | Not reported | Not reported | Previous surgery  IG: 16  CG: 12 | 4 weeks |
| **Duan 2024^11^** | Azathioprine + Exclusive enteral nutrition (AZA: 1 mg/kg/day for the first month, subsequently increased to 2–2.5 mg/kg /day) EEN: last for 3 months immediately following surgery followed by a normal diet) (n=42) | Azathioprine  (AZA: 1 mg/kg/day for the first month, subsequently increased to 2–2.5 mg/kg /day) (n=42) | Ileal 22, Ileocolonic 59 *NR | Not reported | Laparoscopic surgery  IG: 33  CG: 33 | Prior bowel resection  IG: 6  CG: 6 | 2 weeks |
| **Ewe 1999^12^** | Budesonide  (3 mg/d)  (n=40) | Placebo  (n=40) | Ileum 25, colon 15, ileocolon 60  *Not reported | (more than one nomination possible)  IG: failure of medical treatment 11; ileus chronic obstruction, stenosis, stricture 36; fistula, abscess, abdominal mass 25; perforation and peritonitis 1  CG: failure of medical treatment 14; ileus chronic obstruction, stenosis, stricture 35; fistula, abscess, abdominal mass 16, massive bleeding 1, perianal disease 1 | (more than one nomination possible)  IG: ileal resection or stricturoplasty 8; ileo-caecal resection-right hemicolectomy 26, segmental colonic resection 5, colectomy 1  CG: ileal resection or stricturoplasty 7; ileo-caecal resection-right hemicolectomy 23, segmental colonic resection 6, colectomy 3 | Previous operation  IG: 25  CG: 27 | < 2 weeks |
| **Fukushima 2018^13^** | Infliximab  (5 mg/kg at 0, 2, and 6 weeks, followed by every 8 weeks for 2 years)  (n=21) | No treatment  (n=22) | Ileum 26; colon 10; ileocolon 54  *More than 3 intestinal resections, presence of a stoma | IG: Stricture 10,  Obstruction 1, Fistula 1, Abscess 1  CG: Stricture 14,  Obstruction 0, Fistula 4, Abscess 1 | Not reported | Number of previous surgical resection 0/1/2 times  IG: 17/2/0  CG: 15/3/1 | 0 to 4 weeks |
| **Hanauer 2004^14^** | Mesalazine  (3 g/d)  (n=44) | 6-  mercaptopurine  (50 mg/d)  (n=47)  Placebo  (n=40) | Not reported  *Active perianal disease or any active disease in other segments of the intestine | Not reported | Not reported | Not reported | Before postoperative  hospital discharge |
| **Hellers 1999^15^** | Budesonide  (6 mg/d)  (n=63) | Placebo  (n=67) | Not reported  *Septic complications, > 100 cm of terminal ileum resected | IG: Obstruction 36, Disease activity 22, Complications 4, Other reasons 1  CG: Obstruction 42, Disease activity 19, Complications 5, Other reasons 0 | Not reported | Previous resection  IG: 19  CG: 17 | < 2 weeks |
| **Herfarth 2006^16^** | 5-ASA  (4g/d)  (n=37) | Azathioprine  (2.0 to 2.5 mg/kg body weight/d)  (n=42) | Not reported | Not reported | Not reported | Not reported | 2 weeks |
| **Herfarth 2013^17^** | Ciprofloxacin  (1 g/d)  (n=17) | Placebo (n=16) | Non-stricturing, non-penetrating 18; stricturing 55; penetrating 27  *Gross evidence of CD at the operative  margins or in the proximal or distal segments of the intestine, presence of a stoma | Not reported | Not reported | Number of resections, 1/2/3/>3 times  IG: 14/2/0/1  CG: 13/2/1/0 | ≤ 2 weeks |
| **Hirsch 2023^18^** | 6-mercaptopurine (Starting dose: 50 mg/day; escalate every 1–2 weeks; target: 1–1.5 mg/kg)  (n = 16) | adalimumab （week 0-160 mg; week 2-80 mg; week 4-40 mg. 40mg every other week）  (n = 19) | NR *residual inflammatory disease, i.e., residual ileal or colonic inflammation or perianal disease | Not reported | Not reported | Not reported | ≤ 2 weeks |
| **Lochs 2000^19^** | Mesalazine  (4 g/d)  (n=154) | Placebo  (n=170) | IIeal 49; ileocolonic 56; colonic 5  *Short bowel syndrome, presence of an  ileocolonic stoma, more than 3 surgeries | IG: Fistula 1, Stenosis 16, Inflammation 9, Fistula stenosis 5, Fistula + inflammation 12, Stenosis + inflammation 78, Fistula + stenosis + inflammation 28,  No information 3 | IG: radical 112, non-radical 35  CG: radical 123, non-radical 40 | Not reported | ≤ 10 days |
| **Lopez Sanroman 2017^20^** | Azathioprine  (2.5 mg/kg/d)  (n=39) | Adalimumab (160 mg subcutaneously, then 80 mg at Week 2, or 40 mg at Week 4 and every 2 weeks)  (n=45) | Ileal 58, ileocolonic 41  *Postsurgical stoma, resection for short indolent stenosis, inaccessible anastomosis to endoscopy | Not reported | Not reported | Previous resections  IG: 3 (7.7%)  CG: 3 (6.7%) | ≤ 2 weeks |
| **Marteau 2006^21^** | *Lactobacillus*  *johnsonii* LA1  (52218 CFU of lyophilised LA1 /d)  (n=48) | Placebo  (n=50) | Ileum 55; ileocolon 41; colon 4  *Total or subtotal colectomy, intestinal bypass or stricturoplasty, stomy, carcinoma resection, or abscess drainage | Not reported | Not reported | Previous surgery  IG: 7 (15%)  CG: 9 (18%) | ≤ 21 days |
| **McLeod 1995^22^** | Mesalazine  (3 g/d)  (n=88) | Placebo  (n=81) | IIeal 21; ileocolonic 46; colonic 33 | IG: Failure of medical management 43, Fistula or abscess 21, Obstruction 18, Bleeding 3, Perianal disease 0, Other 2  CG: Failure of medical management 44, Fistula or abscess 16, Obstruction 10, Bleeding 3, Perianal disease 2, Other 1 | Nine patients in each group had two resections for Crohn's disease.  IG: Small bowel resection 8, Terminal ileal/ileocolic resection 59, Segmental colon resection 7, Total abdominal colectomy 1, Proctocolectomy 13, Proctectomy 3  CG: Small bowel resection 7, Terminal ileal/ileocolic resection 50, Segmental colon resection 0, Total abdominal colectomy 2, Proctocolectomy 12, Proctectomy 7 | Mean no. of resections (SD)  IG: 1.6 (0.8)  CG: 1.6 (0.8) | ≤ 8 weeks |
| **Mowat 2016^23^** | Mercaptopurine  (1 mg/kg/d) (n=128) | Placebo  (n=112) | Ileal 39; colonic 2; ileocolonic 59  *Need for further surgery, strictureplasty  alone, formation of a stoma | Not reported | Not reported | Previous surgery  IG: 46  CG: 28 | ≤ 3 months |
| **Orlando 2020^24^** | Azathioprine (2.0-2.5 mg/kg/day) (n=22) | Mesalazine (4 gr/die/day) (n=24) | terminal ileum and part of the right colon  *conservative surgery, surgery for Crohn's colitis without ileitis, presence of other unresected lesions, active perianal CD, previous bowel resection comprising more than one meter | IG: Stricturing disease 20, Fistulizing disease 1, abscess 1  CG: Stricturing disease 20, Fistulizing disease 4, abscess 0 | Not reported | Previous bowel resection  IG: 3 (13.6%)  CG: 5 (20.8%) | 2 weeks |
| **Prantera 2002^25^** | LGG probiotic  (2.46 g/d; 6 billion CFU twice daily)  (n=23) | Placebo (n=22) | Ileum 78; ileocolon 13; colon 9  *Active perianal disease; presence of CD in other intestinal tracts; postoperative  septic complications | IG: Obstruction 17, Fistula 6, Failure of medical therapy 0  IG: Obstruction 15, Fistula 4, Failure of medical therapy 3 | Not reported | Previous resection  IG: 5 (21.7%)  CG: 6 (27.3%) | ≤ 10 days |
| **Regueiro 2009^26^** | Infliximab (n=11) | Placebo (n=13) | Ileum only 21; ileum and colon 79  *Not reported | Not reported | Not reported | Number of surgical resections 1/2/3 times  IG: 7/3/1  CG: 9/3/1 | 0 to 4 weeks |
| **Regueiro 2016^27^** | Infliximab  (5 mg/kg q8w) (n=147) | placebo  (n=150) | Ileum 98; colon 55.7; proximal small intestine, stomach, and/or oesophagus 4.1;  perianal 10.1; extra-intestinal manifestations 12.2  *Surgery more than 10 years after CD diagnosis, stricturing disease involving < 10 cm of bowel | Not reported | Not reported | Prior intra-abdominal surgeries 0/1-2/>2 times  IG: 79/63/4  CG: 91/51/8 | ≤ 45 days |
| **Reinisch 2010^28^** | Mesalazine  (4 g/d) +  placebo  azathioprine (n=41) | Azathioprine  (2.0 to 2.5 mg/  kg/d) + placebo mesalazine (n=37) | Not reported  *Short bowel syndrome, an ileocolonic  stoma | Not reported | Not reported | Not reported | 6 to 24  months |
| **Rutgeerts 2005^29^** | Ornidazole  (1 g/d)  (n=40) | Placebo  (n=40) | Not reported  *Pure fibrostenotic disease without biologic inflammation, strictureplasties, 2-  step resections with temporary ileostoma | Not reported | Not reported | Number of resections 1/2/3/>3 times  IG: 26/9/2/1  CG: 31/7/2/0 | < 2 weeks |
| **Savarino 2013^30^** | Mesalazine  (3g/d)  (n=18) | Adalimumab  (160 to 80 mg  0 to 2 weeks  and 40 mg  thereafter) (n=16)  Azathioprine  (2 mg/kg/d) (n=17) | Ileum 49, ileocolonic 51  *Fibrostenotic stricture, macroscopically  active disease not resected at the time of  surgery, and presence of a stoma | Not reported | Not reported | Number of surgical resections 1/2/3 times  IG: 13/4/1  CG1: 21/3/1  CG2: 15/2/0 | 2 to 4 weeks |
| **Sutherland 1997^31^** | Mesalazine  (3 g/d)  (n=31) | Placebo (n=35) | Ileal 49, ileocolonic 50, unknown 1 | Not reported | Not reported | Not reported | 2 to 4 weeks |
| **Tursi 2014^32^** | Infliximab  (5 mg/kg at 0,  2, and thereafter 8-week  intervals)  (n=10) | Adalimumab  (160-80-40  mg/2-week  intervals)  (n=10) | Not reported  *Active perianal disease, the presence of stoma | Not reported | Not reported | Not reported | 4 to 6 weeks |
| **Wenckert 1978^33^** | Sulfasalazine  (3 g/d)  (n=32) | Placebo  (n=34) | n/a | Not reported | Not reported | Not reported | 2 to 4 weeks |
| **Yoshida 2012^34^** | Infliximab  (5 mg/kg at  8-week intervals) + Mesalamine  (1500 mg/day)  (n=15) | Mesalamine  (1500 mg/day)  (n=16) | Ileum 26, ileocolon 74  *Macroscopically active disease missed  during surgery or the presence of abscess | IG: Obstruction 12, Abscess 3  CG: Obstruction 14, Abscess 2 | IG: Ileal resection 4, Ileocecal resection 11  CG: Ileal resection 4, Ileocecal resection 10 | Number of surgical resections 1/≥2 times  IG: 11/4  CG: 10/6 | 0 to 4 weeks |

# **eTable 3.** Included studies’ efficacy outcome definitions and baseline disease activity (n=34)

| **Study ID (Author,**  **Year)** | **Definition of clinical relapse** | **Definition of endoscopic relapse** | **Baseline clinical relapse characteristics** | **Baseline endoscopy relapse characteristics** |
| --- | --- | --- | --- | --- |
| **Ardizzone 2004** | CDAI > 200 | n/a  Surgical relapse: need for  another surgical procedure | CDAI≤150 | NR |
| **Armuzzi 2013** | HBI ≥ 8 | Rutgeerts' score ≥ i2 | NR | NR |
| **Bergman 1976** | X-ray  Typical roentgenological findings for CD | n/a | NR | NR |
| **Bommelaer 2020** | CDAI > 150 | Rutgeerts’ index ≥ i2a | NR | NR |
| **Brignola 1995** | CDAI > 150 | Rutgeerts' score ≥ 3 | NR | NR |
| **Caprilli 1994** | CDAI > 150 and100 points over previous value | n/a | Mesalazine: CDAI 326 (105-538)  No treatment: CDAI 321 (78-540) | NR |
| **Chermesh 2007** | CDAI, definition not stated (presented as mean  change) | Rutgeerts score, definition not stated  (presented as mean change) | NR | NR |
| **De Bruyn 2021** | CDAI ≥ 220 | Rutgeerts i ≥ 2 | Vitamin D: CDAI median (range) 165 (19–394)  Placebo: 136 (1–463) | NR |
| **D'Haens 2008** | CDAI > 250 | Rutgeerts i ≥ 2 | NR | NR |
| **D'Haens 2025** | CDAI increase of >70 points | modified Rutgeerts score ≥i2b | Vedolizumab: 108 (11–312) Placebo: 111 (21–349) | NR |
| **Duan 2024** | CDAI ≥ 150 | Rutgeerts score ≥ i2b | NR | NR |
| **Ewe 1999** | rise in CDAI from 60 up to 200 from the first follow-up or a CDAI > 200 | Rutgeerts i ≥ 2 | NR | Rutgeerts = 0 |
| **Fukushima 2018** | CDAI > 150 | Rutgeerts score  ≥ i3 | IFX: CDAI Mean (range) 151 (64–331); CDAI > 200 n=4  No treatment: 154 (17–355) CDAI > 200 n=5 | NR |
| **Hanauer 2004** | Clinical recurrence grading  > 2 | Rutgeerts i > 1  Radiographic relapse: radiographic recurrence grading > 2 | All clinical relapse free | All endoscopic relapse free |
| **Hellers 1999** | CDAI > 200 | Rutgeerts i ≥ 2 | NR | Baseline Endoscopy score around 0 |
| **Herfarth 2006** | severe endoscopic relapse, withdrawal due to clinical relapse or to adverse drug reaction | NR | NR | NR |
| **Herfarth 2013** | HBI ≥ 5 | Rutgeerts score ≥ i2 or Marteau score ≥ c2 | NR | NR |
| **Hirsch 2023** | CDAI ≥ 150 | Rutgeerts score ≥ i2 | CDAI ≤ 150 | All included patients were in clinical remission |
| **Lochs 2000** | 1 of the following: increase in CDAI above 250; increase in CDAI above 200 but by a minimum of 60 points over the lowest postoperative value for 2 consecutive weeks, indication for surgery; development of a new fistula; and occurrence of a septic complication. | Rutgeerts i ≥ 1 | All patients were in clinical remission. | NR |
| **Lopez Sanroman 2017** | CDAI > 200 | Rutgeerts i ≥ 2 | Postsurgical CDAI< 200:  Azathioprine: 24/38 (63.2%)  Adalimumab: 29/42 (69%) | NR |
| **Marteau 2006** | CDAI ≥ 200 | Rutgeerts i > 1 | NR | NR |
| **McLeod 1995** | Severe symptoms to warrant treatment | Presence of endoscopic or radiological evidence of disease and included both  Asymptomatic and symptomatic patients | Patients who had no gross residual disease were eligible for entry. All in remission. | Patients who had no gross residual disease were eligible for entry. All in remission. |
| **Mowat 2016** | CDAI > 150 and a 100-point increase from baseline and the need for anti-inflammatory rescue treatment or primary surgical intervention | Rutgeerts i ≥ 2 | Patients with residual active Crohn's disease present after surgery were excluded. No clinical relapse. | Patients with residual active Crohn's disease present after surgery were excluded. |
| **Orlando 2020** | CDAI > 200 | Rutgeerts i ≥ 2 | NR | Rutgeerts’ score at randomization  5-ASA: 2.71 ± 0.62  AZA:2.91 ± 0.61 |
| **Prantera 2002** | CDAI > 150 | Rutgeerts i ≥ 2 | NR | NR |
| **Regueiro 2009** | CDAI > 200 | Rutgeerts score ≥ i2 | CDAI >200  Infliximab: 6 (54.5%)  Placebo: 4 (30.8%) | NR |
| **Regueiro 2016** | CDAI > 200 and a ≥70 point increase from baseline | Rutgeerts score ≥ i2 | CDAI <200 | NR |
| **Reinisch 2010** | CDAI score ≥ 200 and an increase of ≥ 60 points from baseline or study drug discontinuation due to lack of efficacy or an intolerable adverse drug reaction | Rutgeerts i ≥ 2 | CDAI <200 | moderate or severe endoscopic recurrence |
| **Rutgeerts 2005** | the occurrence of symptoms. The CDAI at that time needed to be > 250. Clinical recurrence was also diagnosed if reoperation or other Crohn’s disease–related therapy was necessary. | Rutgeerts score ≥ i2 | NR | NR |
| **Savarino 2013** | 1. ≥ 2 on the clinical recurrence grading scale by Hanauer  2. CDAI > 200 | Rutgeerts i ≥ 2  Radiologic relapse: ≥ 2 radiographic recurrence grading scale  HRQOL: IBDQ > 170 | ADA: Mean CDAI (range) 268 (163– 430); CDAI > 200 12 (75%)  AZA: 248 (152– 430); 11 (64.7%)  Mesalamine: 266 (152– 420); 13 (72.2%) | NR |
| **Sutherland 1997** | 1st occurrence of a CDAI > 150 as well as an absolute value of at least 60 points higher than baseline or where physician diagnosed a flare-up of disease, but a full diary card was not available for the calculation of the final CDAI | n/a | CDAI <150 | NR |
| **Tursi 2014** | HBI ≥ 8 | Rutgeerts score ≥ i2 | NR | NR |
| **Wenckert 1978** | Special control charts. The relapses were not based on index calculation. | n/a | Relapse-free | Relapse-free |
| **Yoshida 2012** | 1. CDAI >150  2. IOIBD ≥ 2 | Rutgeerts score ≥ i2 | NR | NR |

# **eTable 4.** Outcomes data reported in the included studies.

| **Study ID (author,**  **Year)** | **Clinical relapse** | **Endoscopic relapse** | **Withdrawals due to adverse events** | **Serious adverse events** | **Total adverse events** |
| --- | --- | --- | --- | --- | --- |
| **Ardizzone 2004** | PA: 31/71 5-ASA: 30/71 | Not reported | PA: 15/71 5-ASA: 6/71 | PA: 15/71 5-ASA: 6/71 | PA: 18/71 5-ASA: 27/71 |
| **Armuzzi 2013** | PA: 2/11 inf: 1/11 | PA: 5/11 inf: 1/11 | PA: 1/11 inf: 0/11 | PA: 0/11 inf: 0/11 | PA: 0/11 inf: 0/11 |
| **Bergman 1976** | pl: 15/40 SulfPred: 24/57 | Not reported | Not reported | Not reported | Not reported |
| **Bommelaer 2020** | PA: 14/31 cur+PA: 12/31 | PA: 18/31 cur+PA: 21/31 | PA: 4/31 cur+PA: 4/31 | PA: 2/31 cur+PA: 5/31 | Not reported |
| **Brignola 1995** | pl: 14/43 5-ASA: 13/44 | pl: 29/43 5-ASA: 17/44 | pl: 3/43 5-ASA: 5/44 | Not reported | pl: 3/43 5-ASA: 5/44 |
| **Caprilli 1994** | pl: 17/55 5-ASA: 11/55 | pl: 36/55 5-ASA: 22/55 | pl: 0/55 5-ASA: 2/55 | Not reported | pl: 0/55 5-ASA: 2/55 |
| **Chermesh 2007** | Reported as continuous outcome | Reported as continuous outcome | pl: 7/10 syn: 7/20 | Not reported | Not reported |
| **De Bruyn 2021** | pl: 20/71 VitD: 22/72 | pl: 61/71 VitD: 64/72 | pl: 8/71 VitD: 7/72 | pl: 23/71 VitD: 15/72 | pl: 53/71 VitD: 59/72 |
| **D'Haens 2008** | pl: 19/41 PA: 11/40 | pl: 32/41 PA: 22/40 | pl: 4/41 PA: 3/40 | Reported as number of events | pl: 32/41 PA: 22/40 |
| **D'Haens 2025** | pl: 8/37 vedo: 9/43 | pl: 23/37 vedo: 10/43 | pl: 1/37 vedo: 1/43 | pl: 2/37 vedo: 3/43 | Reported as number of events |
| **Duan 2024** | PA+EEN: 7/42 PA: 7/42 | PA: 29/42 PA+EEN: 22/42 | PA: 6/42 PA+EEN: 9/42 | PA+EEN: 8/40 PA: 6/40 | PA+EEN: 36/40 PA: 37/40 |
| **Ewe 1999** | pl: 19/40 bude: 14/43 | No extractable quantitative data provided | pl: 5/40 bude: 5/43 | Pl: 2/40  bude: 2/43 | pl: 7/40 bude: 13/43 |
| **Fukushima 2018** | pl: 21/22 inf: 9/21 | pl: 16/22 inf: 12/21 | pl: 0/22 inf: 3/21 | Not reported | pl: 0/22 inf: 3/21 |
| **Hanauer 2004** | pl: 35/40 5-ASA: 33/44  PA: 32/47 | No extractable quantitative data provided | pl: 4/40 5-ASA: 6/44 | pl: 2/40 5-ASA: 0/44 | pl: 4/40 5-ASA: 6/44 |
| **Hellers 1999** | Reported as continuous outcome | Reported as continuous outcome | pl: 5/67 bud: 5/63 | pl: 9/66 bud: 10/63 | pl: 51/66 bud: 44/63 |
| **Herfarth 2006** | PA: 33/42 5-ASA: 27/37 | Not reported | PA: 7/42 5-ASA: 4/37 | Not reported | PA: 29/42 5-ASA: 26/37 |
| **Herfarth 2013** | pl: 8/16 anti: 10/17 | pl: 11/16 anti: 11/17 | pl: 4/16 anti: 1/17 | Not reported | pl: 6/16 anti: 1/17 |
| **Hirsch 2023** | PA: 4/20 ada: 2/21 | PA: 12/16 ada: 9/19 | PA: 0/20 ada: 0/21 | PA: 4/16 ada: 1/19 | PA: 9/16 ada: 8/19 |
| **Lochs 2000** | pl: 59/170 5-ASA: 47/154 | pl: 134/170 5-ASA: 133/154 | Not reported | pl: 9/170 5-ASA: 8/154 | pl: 144/170 5-ASA: 135/154 |
| **Lopez Sanroman 2017** | PA: 14/39 ada: 7/45 | PA: 23/39 ada: 19/45 | PA: 1/39 ada: 9/45 | PA: 4/39 ada: 9/45 | PA: 18/39 ada: 20/45 |
| **Marteau 2006** | pl: 6/50 pro: 9/48 | pl: 26/50 pro: 33/48 | pl: 0/50 pro: 0/48 | Not reported | pl: 6/50 pro: 9/48 |
| **McLeod 1995** | pl: 44/81 5-ASA: 35/88 | Reported as endosscopic or radiology relapse; no extractable quantitative data provided | Not reported | pl: 0/81 5-ASA: 1/88 | pl: 10/81 5-ASA: 7/88 |
| **Mowat 2016** | pl: 70/112 PA: 66/128 | pl: 83/112 PA: 90/128 | pl: 41/112 PA: 39/128 | pl: 2/112 PA: 3/128 | Reported as number of events |
| **Orlando 2020** | 5-ASA: 5/24 PA: 0/22 | 5-ASA: 22/24 PA: 16/22 | 5-ASA: 0/24 PA: 3/22 | 5-ASA: 0/24 PA: 0/22 | Not reported |
| **Prantera 2002** | pl: 8/22 pro: 5/23 | pl: 11/22 pro: 17/23 | pl: 0/22 pro: 0/23 | Not reported | pl: 6/22 pro: 2/23 |
| **Regueiro 2009** | pl: 5/13 inf: 1/11 | pl: 11/13 inf: 1/11 | pl: 1/13 inf: 2/11 | Reported as number of events | pl: 6/13 inf: 7/11 |
| **Regueiro 2016** | pl: 57/150 inf: 62/147 | pl: 101/150 inf: 70/147 | pl: 19/150 inf: 32/147 | Data at the end of the intervention were not reported | Data at the end of the intervention were not reported |
| **Reinisch 2010** | PA: 22/37 5-ASA: 25/41 | PA: 10/37 5-ASA: 1/41 | PA: 10/37 5-ASA: 1/41 | PA: 0/37 5-ASA: 10/41 | PA: 32/37 5-ASA: 34/41 |
| **Rutgeerts 2005** | pl: 15/40 anti: 5/40 | pl: 33/40 anti: 27/40 | pl: 5/40 anti: 12/40 | Not reported | pl: 12/40 anti: 26/38 |
| **Savarino 2013** | ada: 2/16 5-ASA: 9/18  PA: 11/17 | ada: 1/16 5-ASA: 15/18  PA:11/17 | ada: 1/16 5-ASA: 2/18 | Not reported | ada: 11/16 5-ASA: 16/18 |
| **Sutherland 1997** | pl: 8/35 5-ASA: 3/31 | Not reported | Can't distinguish the adverse event related to surgery and non-surgery groups | Can't distinguish the adverse event related to surgery and non-surgery groups | Can't distinguish the adverse event related to surgery and non-surgery groups |
| **Tursi 2014** | Ada: 1/10 inf: 1/10 | Ada: 1/10 inf: 2/10 | Ada: 0/10 inf: 0/10 | Ada: 0/10 inf: 0/10 | Ada: 0/10 inf: 0/10 |
| **Wenckert 1978** | pl: 24/34 sulf: 23/32 | Not reported | pl: 1/34 sulf: 0/32 | Not reported | Not reported |
| **Yoshida 2012** | 5-ASA: 4/16 inf+5-ASA: 3/15 | 5-ASA: 13/16 inf+5-ASA: 4/15 | Not reported | 5-ASA: 0/16 inf+5-ASA: 0/15 | 5-ASA: 0/16 inf+5-ASA: 0/15 |

PA: purine analogues, 5-ASA: 5-Aminosalicylic Acid, inf: infliximab, pl: placebo, cur: curcumin, syn: sunbiotics, VitD: Vitamin D, vedo: vedolizumab, EEN: external nutrition, bude: budesonide, anti: antibiotics, ada: adalimumab, pro: probiotics, sulf: sulfasalazi

# **eTable 5**. Excluded studies and reasons for exclusion.

| **Study** | **Reason for exclusion** |
| --- | --- |
| **Angelberger 2013^35^** | Not an RCT; a post hoc analysis of a subset of participants of an RCT |
| **Buisson 2021^36^** | Not an RCT |
| **De Cruz 2012^37^** | Wrong study design;partially randomised |
| **De Cruz 2013a^38^** | Wrong study design;partially randomised |
| **De Cruz 2013b^39^** | Wrong study design;partially randomised |
| **De Cruz 2013c^40^** | Wrong study design;partially randomised |
| **De Cruz 2015a^41^** | Wrong study design;partially randomised |
| **Ewe 1980^42^** | Not an RCT |
| **Ewe 1981^43^** | Not an RCT |
| **Ferrante 2014^44^** | Wrong intervention; AZA vs AZA |
| **Kamm 2014^45^** | Wrong study design; partially randomised |
| **Kennedy 2015^46^** | Not an RCT |
| **Liao 2009^47^** | Wrong intervention; herb |
| **McLeod 1997^48^** | Not an RCT;non-randomised follow-up of McLeod 1995 |
| **NCT00074542^49^** | Wrong intervention;nutritional supplements |
| **NCT01696942^50^** | Terminated trial |
| **NCT02247258^51^** | Trial was terminated due to slow recruitment |
| **NCT02255370^52^** | Wrong intervention;nutritional supplements |
| **NCT02997059^53^** | Terminated trial |
| **Papamichael 2012^54^** | Not an RCT |
| **Regueiro 2013^55^** | Not an RCT;follow-up of the control group Regueiro 2009 |
| **Regueiro 2014^56^** | Not an RCT;follow-up of the control group Regueiro 2009 |
| **Ren 2013^57^** | Wrong intervention;herb |
| **Steinhart 1992^58^** | Not an RCT |
| **Tao 2009^59^** | Wrong intervention;herb |
| **Wright 2014^60^** | Wrong intervention;colonoscopy vs no colonoscopy |
| **Wright 2015** | Wrong intervention;colonoscopy vs no colonoscopy |
| **Yamamoto 2009^61^** | Not an RCT |
| **Zhu 2015^62^** | Wrong intervention;herb |

# **eTable 6.** Predefined Magnitude Effect Thresholds.

|  | **Trivial to Small** | **Small to Moderate** | **Moderate to Large** |
| --- | --- | --- | --- |
| **Clinical Remission** | 10% | 20% | 31% |
|  |  |  |  |
| **Clinical Response** | 13% | 23% | 35% |
|  |  |  |  |
| **Endoscopic relapse** | 9% | 17% | 28% |
|  |  |  |  |
| **Withdrawals due to adverse events** | 7% | 14% | 23% |
|  |  |  |  |
| **Serious Adverse Events** | 6% | 11% | 17% |
|  |  |  |  |
| **Total Adverse Events** | 9% | 16% | 24% |
|  |  |  |  |

# **eTables 7**. SUMMARY OF FINDINGS TABLES AND GRADE DECISIONS

**Summary of Findings Tables and GRADE decisions (Clinical Relapse)**

| **Clinical relapse** | | | | | | | |
| --- | --- | --- | --- | --- | --- | --- | --- |
| Patient or population: people with Crohn's disease post-surgery | | | | | | | |
| Settings: hospital setting | | | | | | | |
| Intervention: advanced therapies/purine analogues/ Purine analogues + enteral nutrition/5-ASA/sulfasalazine/ antibiotics/probiotics/synbiotics/ Curcumin + Purine analogues/vitamin D/ budesonide/ Sulfasalazine + Prednisolone | | | | | | | |
| Comparison: placebo | | | | | | | |
| **Treatment**  **(Timepoint of outcome measurement)** | **Network evidence** | | **Anticipated absolute effects for network estimate** | | | **NNT (95% CI)** | **Notes** |
|  | **RR** | **Certainty** | **Risk with Placebo^a^** | **Risk with Agent^b^ (95% CI)** | **% Risk Difference with Agent^c^ (95% CI)** |  |  |
|  | **(95% CI)** |  |  |  |  |  |  |
| **Adalimumab**  **(12-24 months)** | **0.31 (0.16 to 0.6)** | **Moderate** | **424 per 1,000** | **131 per 1,000 (68 to 254)** | **29.1% less (35.5% less to 16.8%less)** | **3 (3 to 6)** | **Probably moderate effect better than placebo (small to large)** |
|  |  |  |  |  |  |  |  |
|  |  | ⊕⊕⊕⊖ |  |  |  |  |  |
| Curcumin + Purine analogues  (6 months) | 0.68 (0.35 to 1.34) | Very low | 424 per 1,000 | 288 per 1,000 (148 to 568) | 13.5% less (27.7% less to 14.4% more) | NA | The data is very uncertain |
|  |  | ⊕⊖⊖⊖ |  |  |  |  |  |
|  |  |  |  |  |  |  |  |
| Infliximab+5ASA  (36 months) | 0.63 (0.16 to 2.46) | Very Low | 424 per 1,000 | 267 per 1,000 (68 to 1000) | 15.6% less (35.5% less to 57.6% more) | NA | The data is very uncertain |
|  |  | ⊕⊖⊖⊖ |  |  |  |  |  |
|  |  |  |  |  |  |  |  |
| Budesonide  (12 months) | 0.69 (0.37 to 1.26) | Very low | 424 per 1,000 | 293 per 1,000 (157 to 534) | 13.3% less (26.5% less to 10.8% more) | NA | The data is very uncertain |
|  |  | ⊕⊖⊖⊖ |  |  |  |  |  |
|  |  |  |  |  |  |  |  |
| Infliximab  (12-24 months) | 0.79 (0.57 to 1.07) | Very low | 424 per 1,000 | 335 per 1,000 (242 to 454) | 9.1% less (18.1% less to 3.1% more) | NA | The data is very uncertain |
|  |  | ⊕⊖⊖⊖ |  |  |  |  |  |
|  |  |  |  |  |  |  |  |
| **5-ASA**  **(12-72 months)** | **0.79 (0.66 to 0.94)** | **Low** | **424 per 1,000** | **335 per 1,000 (280 to 399)** | **8.9% less (14.4% less to 2.4%less)** | **11 (7 to 40)** | **Maybe trivial effect better than placebo (trivial to small)** |
|  |  |  |  |  |  |  |  |
|  |  | **⊕⊕⊖⊖** |  |  |  |  |  |
| **Purine analogues**  **(6-36 months)** | **0.79 (0.66 to 0.96)** | **Low** | **424 per 1,000** | **335 per 1,000 (280 to 407)** | **8.7% less (14.6% less to 1.6%less)** | **11 (7 to 59)** | **Maybe trivial effect better than placebo (trivial to small)** |
|  |  | **⊕⊕⊖⊖** |  |  |  |  |  |
|  |  |  |  |  |  |  |  |
| Antibiotics  (6-12 months) | 0.76 (0.44 to 1.33) | Very low | 424 per 1,000 | 322 per 1,000 (187 to 564) | 10% less (23.9% less to 14.1% more) | NA | The data is very uncertain |
|  |  | ⊕⊖⊖⊖ |  |  |  |  |  |
|  |  |  |  |  |  |  |  |
| Purine analogues + enteral nutrition  (12 months) | 0.79 (0.29 to 2.19) | Very Low | 424 per 1,000 | 335 per 1,000 (123 to 929) | 8.7% less (30.2% less to 50.4% more) | NA | The data is very uncertain |
|  |  | ⊕⊖⊖⊖ |  |  |  |  |  |
|  |  |  |  |  |  |  |  |
| Vedolizumab  (6 months) | 0.97 (0.4 to 2.35) | Very Low | 424 per 1,000 | 411 per 1,000 (170 to 996) | 1.4% less (25.5% less to 57.4% more) | NA | The data is very uncertain |
|  |  | ⊕⊖⊖⊖ |  |  |  |  |  |
|  |  |  |  |  |  |  |  |
| Probiotics  (6-12 months) | 0.97 (0.48 to 1.95) | Very Low | 424 per 1,000 | 411 per 1,000 (204 to 827) | 1.5% less (22.1% less to 40.2% more) | NA | The data is very uncertain |
|  |  | ⊕⊖⊖⊖ |  |  |  |  |  |
|  |  |  |  |  |  |  |  |
| Sulfasalazine  (18 months) | 1.02 (0.68 to 1.54) | Very low | 424 per 1,000 | 432 per 1,000 (288 to 653) | 0.8% more (13.8% less to 22.7% more) | NA | The data is very uncertain |
|  |  | ⊕⊕⊖⊖ |  |  |  |  |  |
|  |  |  |  |  |  |  |  |
| Vitamin D  (6 months) | 1.08 (0.61 to 1.93) | Very Low | 424 per 1,000 | 458 per 1,000 (259 to 818) | 3.6% more (16.6% less to 39.6% more) | NA | The data is very uncertain |
|  |  | ⊕⊖⊖⊖ |  |  |  |  |  |
|  |  |  |  |  |  |  |  |
|  |  |  |  |  |  |  |  |
|  |  |  |  |  |  |  |  |
| Sulfasalazine + Prednisolone  (36 months) | 1.12 (0.63 to 1.99) | Very low | 424 per 1,000 | 475 per 1,000 (267 to 844) | 5.2% more (15.5% less to 42% more) | NA | The data is very uncertain |
|  |  | ⊕⊖⊖⊖ |  |  |  |  |  |
|  |  |  |  |  |  |  |  |
| **GRADE Working Group grades of evidence** | | | | | | | |
| **High certainty**: we are very confident that the true effect lies close to that of the estimate of the effect. | | | | | | | |
| **Moderate certainty**: we are moderately confident in the effect estimate; the true effect is likely to be close to the estimate of the effect, but there is a possibility that it is substantially different. | | | | | | | |
| **Low certainty**: our confidence in the effect estimate is limited; the true effect may be substantially different from the estimate of the effect. | | | | | | | |
| **Very low certainty**: we have very little confidence in the effect estimate; the true effect is likely to be substantially different from the estimate of effect. | | | | | | | |
|  | | | | | | | |
| CI: confidence interval; RR: risk ratio | | | | | | | |
|  | | | | | | | |
| ^a^ The risk with placebo has been calculated based on the cumulative placebo rates of all studies with a placebo arm.  ^b^The risk with treatment has been calculated by multiplying the risk with control with the RR (95% CI). If the calculation results in more than 1000 per 1000 people the number has been capped to 1000. Numbers have been rounded up to the closest whole number.  ^c^ The % risk difference has been calculated by subtracting the risk with control from the risk with treatment(95% CI) and dividing by 10. If the calculation results in more than 100% the number has been capped to 100%. Numbers have been rounded up to the closest whole number.  * Interventions in bold indicate treatments whose confidence intervals did not cross the line of no effect. | | | | | | | |

| **Sucra** | **Intervention (n=15)** | **network estimate RR** | **lower 95% CI** | **higher 95% CI** | **Number of direct studies** | **Direct GRADE** | **Reasons for direct downgrade** | **Indirect GRADE** | **Reasons for indirect downgrade** | **Network GRADE** | **Reasons for network downgrade** |
| --- | --- | --- | --- | --- | --- | --- | --- | --- | --- | --- | --- |
| **1** | **Adalimumab** | **0.31** | **0.16** | **0.6** | **0** | **x** | **x** | **moderate** | **once due to rob in the strongest loop** | **moderate** | **none** |
| 2 | Curcumin + Purine analogues | 0.68 | 0.35 | 1.34 | 0 | x | x | moderate | once due to rob in the strongest loop | very low | twice due to imprecision |
| 3 | Infliximab + 5ASA | 0.63 | 0.16 | 2.46 | 0 | x | x | moderate | once due to rob in the strongest loop | very low | twice due to imprecision |
| 4 | Budesonide | 0.69 | 0.37 | 1.26 | 1 | moderate | once due to rob | x | x | very low | twice due to imprecision |
| 5 | Infliximab | 0.79 | 0.57 | 1.07 | 3 | low | once due to rob, once due to inconsistency | low | twice due to rob in the strongest loop | very low | twice due to imprecision |
| **6** | **5-ASA** | **0.79** | **0.66** | **0.94** | **6** | **moderate** | **once due to rob** | **moderate** | **once due to rob in the strongest loop** | **low** | **once due to imprecision** |
| **7** | **Purine analogues** | **0.79** | **0.66** | **0.96** | **3** | **moderate** | **once due to rob** | **moderate** | **once due to rob in the strongest loop** | **low** | **once due to imprecision** |
| 8 | Antibiotics | 0.76 | 0.44 | 1.33 | 2 | low | once due to rob, once due to inconsistency | x | **x** | very low | twice due to imprecision |
| 9 | Purine analogues + enteral nutrition | 0.79 | 0.29 | 2.19 | 0 | x | x | moderate | once due to rob in the strongest loop | very low | twice due to imprecision |
| 10 | Vedolizumab | 0.97 | 0.4 | 2.35 | 1 | moderate | once due to rob | x | **x** | very low | twice due to imprecision |
| 11 | Probiotics | 0.97 | 0.48 | 1.95 | 2 | moderate | once due to rob | x | **x** | very low | twice due to imprecision |
| 12 | Sulfasalazine | 1.02 | 0.68 | 1.54 | 1 | moderate | once due to rob | x | **x** | very low | twice due to imprecision |
| 13 | Vitamin D | 1.08 | 0.61 | 1.93 | 1 | moderate | once due to rob | x | **x** | very low | twice due to imprecision |
| 14 | Placebo | 1 | 1 | 1 |  |  |  |  |  |  |  |
| 15 | Sulfasalazine + Prednisolone | 1.12 | 0.63 | 1.99 | 1 | low | twice due to rob | x | **x** | very low | twice due to imprecision |

**Summary of Findings Tables and GRADE decisions (Endoscopic Relapse)**

| **Endoscopic relapse** | | | | | | | |
| --- | --- | --- | --- | --- | --- | --- | --- |
| Patient or population: people with Crohn's disease post-surgery | | | | | | | |
| Settings: hospital setting | | | | | | | |
| Intervention: advanced therapies/purine analogues/ Purine analogues + enteral nutrition /5-ASA / antibiotics/probiotics/ Curcumin + Purine analogues/vitamin D | | | | | | | |
| Comparison: placebo | | | | | | | |
| **Treatment**  **(Timepoint of outcome measurement)** | **Network evidence** | | **Anticipated absolute effects for network estimate** | | | **NNT (95% CI)** | **Notes** |
|  | **RR** | **Certainty** | **Risk with Placebo^a^** | **Risk with Agent^b^ (95% CI)** | **% Risk Difference with Agent^c^ (95% CI)** |  |  |
|  | **(95% CI)** |  |  |  |  |  |  |
| **Infliximab + 5-ASA**  **(12 months)** | **0.28 (0.1 to 0.78)** | **Very low** | **721 per 1,000** | **202 per 1,000 (72 to 562)** | **52.2% less (65% less to 16.1%less)** | **2 (2 to 6)** | **The evidence is very uncertain** |
|  |  |  |  |  |  |  |  |
|  |  | **⊕⊖⊖⊖** |  |  |  |  |  |
| **Vedolizumab**  **(6 months)** | **0.37 (0.17 to 0.8)** | **Moderate** | **721 per 1,000** | **267 per 1,000 (123 to 577)** | **45.1% less (59.5% less to 14.1%less)** | **2 (2 to 7)** | **Maybe large effect better than placebo (small to large)** |
|  |  |  |  |  |  |  |  |
|  |  | **⊕⊕⊕⊖** |  |  |  |  |  |
| **Adalimumab**  **(12-24 months)** | **0.47 (0.27 to 0.8)** | **Low** | **721 per 1,000** | **339 per 1,000 (195 to 577)** | **38.5% less (52.5% less to 14.6%less)** | **3 (2 to 7)** | **Maybe large effect better than placebo (small to large)** |
|  |  |  |  |  |  |  |  |
|  |  | **⊕⊕⊖⊖** |  |  |  |  |  |
| Purine analogues + enteral nutrition  (12 months) | 0.6 (0.31 to 1.16) | Very low | 721 per 1,000 | 433 per 1,000 (224 to 836) | 29.2% less (50% less to 11.3%more) | NA | The evidence is very uncertain |
|  |  |  |  |  |  |  |  |
|  |  | ⊕⊖⊖⊖ |  |  |  |  |  |
| **Infliximab**  **(12-24 months)** | **0.65 (0.44 to 0.96)** | **Very low** | **721 per 1,000** | **469 per 1,000 (317 to 692)** | **25.2% less (40.2% less to 3.2%less)** | **4 (2 to 34)** | **The evidence is very uncertain** |
|  |  |  |  |  |  |  |  |
|  |  | **⊕⊖⊖⊖** |  |  |  |  |  |
| Purine analogues  (6-36 months) | 0.78 (0.58 to 1.06) | Very low | 721 per 1,000 | 562 per 1,000 (418 to 764) | 15.5% less (30.2% less to 4.3%more) | NA | The evidence is very uncertain |
|  |  |  |  |  |  |  |  |
|  |  | ⊕⊖⊖⊖ |  |  |  |  |  |
| Antibiotics  (6-12 months) | 0.86 (0.57 to 1.32) | Very low | 721 per 1,000 | 620 per 1,000 (411 to 952) | 9.8% less (31.3% less to 23%more) | NA | The evidence is very uncertain |
|  |  |  |  |  |  |  |  |
|  |  | ⊕⊖⊖⊖ |  |  |  |  |  |
| 5-ASA  (12-24 months) | 0.84 (0.64 to 1.12) | Very low | 721 per 1,000 | 606 per 1,000 (461 to 808) | 11.5% less (26% less to 8.7%more) | NA | The evidence is very uncertain |
|  |  |  |  |  |  |  |  |
|  |  | ⊕⊖⊖⊖ |  |  |  |  |  |
| Curcumin + Purine analogues  (6 months) | 0.92 (0.46 to 1.81) | Very low | 721 per 1,000 | 663 per 1,000 (332 to 1000) | 6.1% less (38.8% less to 27.9%more) | NA | The evidence is very uncertain |
|  |  |  |  |  |  |  |  |
|  |  | ⊕⊖⊖⊖ |  |  |  |  |  |
| Vitamin D  (6 months) | 1.03 (0.63 to 1.69) | Very low | 721 per 1,000 | 743 per 1,000 (454 to 1000) | 2.5% more (26.5% less to 27.9% more) | NA | The evidence is very uncertain |
|  |  |  |  |  |  |  |  |
|  |  | ⊕⊖⊖⊖ |  |  |  |  |  |
| Probiotics  (6-12 months) | 1.39 (0.89 to 2.15) | Very low | 721 per 1,000 | 1000 per 1,000 (642 to 1000) | 27.8% more (7.8% less to 27.9% more) | NA | The evidence is very uncertain |
|  |  |  |  |  |  |  |  |
|  |  | ⊕⊖⊖⊖ |  |  |  |  |  |
| **GRADE Working Group grades of evidence** | | | | | | | |
| **High certainty**: we are very confident that the true effect lies close to that of the estimate of the effect. | | | | | |  |  |
| **Moderate certainty**: we are moderately confident in the effect estimate; the true effect is likely to be close to the estimate of the effect, but there is a possibility that it is substantially different. | | | | | | | |
| **Low certainty**: our confidence in the effect estimate is limited; the true effect may be substantially different from the estimate of the effect. | | | | | | | |
| **Very low certainty**: we have very little confidence in the effect estimate; the true effect is likely to be substantially different from the estimate of effect. | | | | | | | |
|  | | | | | | | |
| CI: confidence interval; RR: risk ratio | | | | | | | |
|  | | | | | | | |
| ^a^ The risk with placebo has been calculated based on the cumulative placebo rates of all studies with a placebo arm.  ^b^The risk with treatment has been calculated by multiplying the risk with control with the RR (95% CI). If the calculation results in more than 1000 per 1000 people, the number has been capped to 1000. Numbers have been rounded up to the closest whole number.  ^c^ The % risk difference has been calculated by subtracting the risk with control from the risk with treatment(95% CI) and dividing by 10. If the calculation results in more than 100% the number has been capped to 100%. Numbers have been rounded up to the closest whole number.  * Interventions in bold indicate treatments whose confidence intervals did not cross the line of no effect. | | | | | | | |

| **Sucra** | **Intervention (n=12)** | **network estimate RR** | **lower 95% CI** | **higher 95% CI** | **Number of direct studies** | **Direct GRADE** | **Reasons for direct downgrade** | **Indirect GRADE** | **Reasons for indirect downgrade** | **Network GRADE** | **Reasons for network downgrade** |
| --- | --- | --- | --- | --- | --- | --- | --- | --- | --- | --- | --- |
| **1** | **Infliximab+ 5-ASA** | **0.28** | **0.1** | **0.78** | **0** | **x** | **x** | **very low** | **once due to rob, twice due to inconsistency in the strongest loop** | **very low** | **none** |
| **2** | **Vedolizumab** | **0.37** | **0.17** | **0.8** | **1** | **moderate** | **once due to rob** | **x** | **x** | **moderate** | **none** |
| **3** | **Adalimumab** | **0.47** | **0.27** | **0.8** | **0** | **x** | **x** | **low** | **once due to rob, once due to inconsistency in the strongest loop** | **low** | **none** |
| 4 | Purine analogues + enteral nutrition | 0.6 | 0.31 | 1.16 | 0 | x | x | moderate | once due to rob in the strongest loop | very low | twice due to imprecision |
| **5** | **Infliximab** | **0.65** | **0.44** | **0.96** | **3** | **low** | **once due to rob, once due to inconsistency** | **low** | **once due to rob, once due to inconsistency in the strongest loop** | **very low** | **once due to imprecision** |
| 6 | Purine analogues | 0.78 | 0.58 | 1.06 | 2 | low | once due to rob, once due to inconsistency | moderate | once due to rob in the strongest loop | very low | twice due to imprecision |
| 7 | Antibiotics | 0.86 | 0.57 | 1.32 | 2 | moderate | once due to rob | x | x | very low | twice due to imprecision |
| 8 | 5-ASA | 0.84 | 0.64 | 1.12 | 3 | low | once due to rob, once due to inconsistency | x | x | very low | twice due to imprecision |
| 9 | Curcumin+ Purine analogues | 0.92 | 0.46 | 1.81 | 0 | x | x | moderate | once due to rob in the strongest loop | very low | twice due to imprecision |
| 10 | vitamin D | 1.03 | 0.63 | 1.69 | 1 | moderate | once due to rob | x | **x** | very low | twice due to imprecision |
| 11 | Placebo | 1 |  |  |  |  |  |  |  |  |  |
| 12 | Probiotics | 1.39 | 0.89 | 2.15 | 2 | moderate | once due to rob | x | x | very low | twice due to imprecision |

**Summary of Findings Tables and GRADE decisions (Withdrawal due to adverse events)**

| Withdrawals due to adverse events | | | | | | | |
| --- | --- | --- | --- | --- | --- | --- | --- |
| Patient or population: people with Crohn's disease post-surgery | | | | | | | |
| Settings: hospital setting | | | | | | | |
| Intervention: advanced therapies/purine analogues/ Purine analogues + enteral nutrition /5-ASA / antibiotics/probiotics/ Curcumin + Purine analogues/vitamin D/synbiotics/budesonide/sulfasalazine | | | | | | | |
| Comparison: placebo | | | | | | | |
| **Treatment**  **(Timepoint of outcome measurement)** | **Network evidence** | | **Anticipated absolute effects for network estimate** | | | **NNT (95% CI)** | **Notes** |
|  | **RR** | **Certainty** | **Risk with Placebo^a^** | **Risk with Agent^b^ (95% CI)** | **% Risk Difference with Agent^c^ (95% CI)** |  |  |
|  | **(95% CI)** |  |  |  |  |  |  |
| Synbiotics  (24 months) | 0.5 (0.15 to 1.63) | Very low | 137 per 1,000 | 69 per 1,000 (21 to 223) | 6.8% less (11.6% less to 8.6%more) | NA | The evidence is very uncertain |
|  |  | ⊕⊖⊖⊖ |  |  |  |  |  |
| Sulfasalazine  (18 months) | 0.35 (0.01 to 9.6) | Very low | 137 per 1,000 | 48 per 1,000 (1 to 1000) | 8.9% less (13.6% less to 86.3%more) | NA | The evidence is very uncertain |
|  |  | ⊕⊖⊖⊖ |  |  |  |  |  |
| 5-ASA  (12-36 months) | 0.88 (0.41 to 1.92) | Very low | 137 per 1,000 | 121 per 1,000 (56 to 263) | 1.6% less (8.1% less to 12.6%more) | NA | The evidence is very uncertain |
|  |  | ⊕⊖⊖⊖ |  |  |  |  |  |
| Vedolizumab  (6 months) | 0.86 (0.05 to 15.53) | Very low | 137 per 1,000 | 118 per 1,000 (7 to 1000) | 1.9% less (13% less to 86.3%more) | NA | The evidence is very uncertain |
|  |  | ⊕⊖⊖⊖ |  |  |  |  |  |
| Vitamin D  (6 months) | 0.86 (0.23 to 3.33) | Very low | 137 per 1,000 | 118 per 1,000 (32 to 452) | 1.9% less (10.5% less to 31.5%more) | NA | The evidence is very uncertain |
|  |  | ⊕⊖⊖⊖ |  |  |  |  |  |
| Budesonide  (12 months) | 0.99 (0.34 to 2.88) | Very low | 137 per 1,000 | 136 per 1,000 (47 to 395) | 0.1% less (9% less to 25.8%more) | NA | The evidence is very uncertain |
|  |  | ⊕⊖⊖⊖ |  |  |  |  |  |
| Antibiotics  (6-12 months) | 1.33 (0.42 to 4.21) | Very low | 137 per 1,000 | 182 per 1,000 (58 to 577) | 4.5% more (7.9% less to 44%more) | NA | The evidence is very uncertain |
|  |  | ⊕⊖⊖⊖ |  |  |  |  |  |
| Curcumin + Purine analogues  (6 months) | 1.42 (0.25 to 7.99) | Very low | 137 per 1,000 | 195 per 1,000 (34 to 1000) | 5.8% more (10.3% less to 86.3%more) | NA | The evidence is very uncertain |
|  |  | ⊕⊖⊖⊖ |  |  |  |  |  |
| Purine analogues  (6-36 months) | 1.42 (0.73 to 2.75) | Very low | 137 per 1,000 | 195 per 1,000 (100 to 377) | 5.8% more (3.7% less to 24%more) | NA | The evidence is very uncertain |
|  |  | ⊕⊖⊖⊖ |  |  |  |  |  |
| Infliximab  (12-24 months) | 1.84 (0.75 to 4.53) | Very low | 137 per 1,000 | 252 per 1,000 (103 to 621) | 11.5% more (3.4% less to 48.4%more) | NA | The evidence is very uncertain |
|  |  | ⊕⊖⊖⊖ |  |  |  |  |  |
| Purine analogues + enteral nutrition  (12 months) | 2.12 (0.48 to 9.38) | Very low | 137 per 1,000 | 290 per 1,000 (66 to 1000) | 15.3% more (7.1% less to 86.3%more) | NA | The evidence is very uncertain |
|  |  | ⊕⊖⊖⊖ |  |  |  |  |  |
| Adalimumab  (12-24 months) | 2.65 (0.48 to 14.68) | Very low | 137 per 1,000 | 363 per 1,000 (66 to 2011) | 22.6% more (7.1% less to 187.4%more) | NA | The evidence is very uncertain |
|  |  | ⊕⊖⊖⊖ |  |  |  |  |  |
| GRADE Working Group grades of evidence | | | | | | | |
| **High certainty:** we are very confident that the true effect lies close to that of the estimate of the effect. | | | | | | | |
| **Moderate certainty:** we are moderately confident in the effect estimate; the true effect is likely to be close to the estimate of the effect, but there is a possibility that it is substantially different. | | | | | | | |
| **Low certainty:** our confidence in the effect estimate is limited; the true effect may be substantially different from the estimate of the effect. | | | | | | | |
| **Very low certainty:** we have very little confidence in the effect estimate; the true effect is likely to be substantially different from the estimate of effect. | | | | | | | |
|  | | | | | | | |
| CI: confidence interval; RR: risk ratio | | | | | | | |
|  | | | | | | | |
| ^a^ The risk with placebo has been calculated based on the cumulative placebo rates of all studies with a placebo arm.  ^b^The risk with treatment has been calculated by multiplying the risk with control with the RR (95% CI). If the calculation results in more than 1000 per 1000 people the number has been capped to 1000. Numbers have been rounded up to the closest whole number.  ^c^ The % risk difference has been calculated by subtracting the risk with control from the risk with treatment(95% CI) and dividing by 10. If the calculation results in more than 100% the number has been capped to 100%. Numbers have been rounded up to the closest whole number. | | | | | | | |

| Sucra | Intervention (n=13) | network estimate RR | lower 95%CI | higher 95% CI | Number of direct studies | Direct GRADE | Reasons for direct downgrade | Indirect GRADE | Reasons for indirect downgrade | Network GRADE |
| --- | --- | --- | --- | --- | --- | --- | --- | --- | --- | --- |
| 1 | Synbiotics | 0.50 | 0.15 | 1.63 | 1 | low | twice due to rob | x | x | very low |
| 2 | Sulfasalazine | 0.35 | 0.01 | 9.6 | 1 | moderate | once due to rob | x | x | very low |
| 3 | 5-ASA | 0.88 | 0.41 | 1.92 | 3 | moderate | once due to rob | moderate | once due to rob in the strongest loop | very low |
| 4 | Vedolizumab | 0.86 | 0.05 | 15.53 | 1 | moderate | once due to rob | x | x | very low |
| 5 | Vitamin D | 0.86 | 0.23 | 3.3 | 1 | moderate | once due to rob | x | x | very low |
| 6 | Placebo |  |  |  |  |  |  |  |  |  |
| 7 | Budesonide | 0.99 | 0.34 | 2.88 | 2 | low | twice due to rob | x | x | very low |
| 8 | Antibiotics | 1.33 | 0.42 | 4.21 | 2 | low | once due to rob, once due to inconsistency | x | x | very low |
| 9 | Curcumin+ Purine analogues | 1.42 | 0.25 | 7.99 | 0 | x | x | moderate | once due to rob in the strongest loop | very low |
| 10 | Purine analogues | 1.42 | 0.73 | 2.75 | 3 | moderate | once due to rob | moderate | once due to rob in the strongest loop | very low |
| 11 | Infliximab | 1.84 | 0.75 | 4.53 | 3 | moderate | once due to rob | moderate | once due to rob in the strongest loop | very low |
| 12 | Purine analogues +enteral nutrition | 2.12 | 0.48 | 9.38 | 0 | x | x | moderate | once due to rob in the strongest loop | very low |
| 13 | Adalimumab | 2.65 | 0.48 | 14.68 | 0 | x | x | moderate | once due to rob in the strongest loop | very low |

**Summary of Findings Tables and GRADE decisions (Serious adverse events)**

| Serious adverse events | | | | | | | |
| --- | --- | --- | --- | --- | --- | --- | --- |
| Patient or population: people with Crohn's disease post-surgery | | | | | | | |
| Settings: hospital setting | | | | | | | |
| Intervention: Intervention: advanced therapies/purine analogues/ purine analogues + enteral nutrition /5-ASA /curcumin + purine analogues/vitamin D/budesonide | | | | | | | |
| Comparison: placebo | | | | | | | |
| **Treatment**  **(Timepoint of outcome measurement)** | **Network evidence** | | **Anticipated absolute effects for network estimate** | | | **NNT (95% CI)** | **Notes** |
|  | **RR** | **Certainty** | **Risk with Placebo^a^** | **Risk with Agent^b^ (95% CI)** | **% Risk Difference with Agent^c^ (95% CI)** |  |  |
|  | **(95% CI)** |  |  |  |  |  |  |
| Vitamin D  (6 months) | 0.67 (0.15 to 3.08) | Very low | 78 per 1,000 | 52 per 1,000 (12 to 240) | 2.6% less (6.6% less to 16.2% more) | NA | The evidence is very uncertain |
|  |  | ⊕⊖⊖⊖ |  |  |  |  |  |
| Adalimumab  (12-13 months) | 1.04 (0.15 to 7.17) | Very low | 78 per 1,000 | 80 per 1,000 (12 to 552) | 0.3% more (6.6% less to 48.1% more) | NA | The evidence is very uncertain |
|  |  | ⊕⊖⊖⊖ |  |  |  |  |  |
| 5-ASA  (12-72 months) | 0.94 (0.29 to 3.11) | Very low | 78 per 1,000 | 72 per 1,000 (22 to 239) | 0.5% less (5.5% less to 16.5% more) | NA | The evidence is very uncertain |
|  |  | ⊕⊖⊖⊖ |  |  |  |  |  |
| Purine analogues  (6-36 months) | 1.12 (0.32 to 3.99) | Very low | 78 per 1,000 | 86 per 1,000 (25 to 307) | 0.9% more (5.3% less to 23.3% more) | NA | The evidence is very uncertain |
|  |  | ⊕⊖⊖⊖ |  |  |  |  |  |
| Budesonide  (12 months) | 1.08 (0.28 to 4.17) | Very low | 78 per 1,000 | 83 per 1,000 (22 to 321) | 0.6% more (5.6% less to 24.7% more) | NA | The evidence is very uncertain |
|  |  | ⊕⊖⊖⊖ |  |  |  |  |  |
| Purine analogue + enteral nutrition  (12 months) | 1.5 (0.18 to 12.57) | Very low | 78 per 1,000 | 116 per 1,000 (14 to 968) | 3.9% more (6.4% less to 90.2% more) | NA | The evidence is very uncertain |
|  |  | ⊕⊖⊖⊖ |  |  |  |  |  |
| Vedolizumab  (6 months) | 1.29 (0.14 to 12.08) | Very low | 78 per 1,000 | 99 per 1,000 (11 to 930) | 2.3% more (6.7% less to 86.4% more) | NA | The evidence is very uncertain |
|  |  | ⊕⊖⊖⊖ |  |  |  |  |  |
| Curcumin + Purine analogues  (6 months) | 2.81 (0.24 to 32.78) | Very low | 78 per 1,000 | 216 per 1,000 (18 to 1000) | 14.1% more (5.9% less to 92.2% more) | NA | The evidence is very uncertain |
|  |  | ⊕⊖⊖⊖ |  |  |  |  |  |
| GRADE Working Group grades of evidence | | | | | | | |
| **High certainty:** we are very confident that the true effect lies close to that of the estimate of the effect. | | | | | | | |
| **Moderate certainty:** we are moderately confident in the effect estimate; the true effect is likely to be close to the estimate of the effect, but there is a possibility that it is substantially different. | | | | | | | |
| **Low certainty:** our confidence in the effect estimate is limited; the true effect may be substantially different from the estimate of the effect. | | | | | | | |
| **Very low certainty:** we have very little confidence in the effect estimate; the true effect is likely to be substantially different from the estimate of effect. | | | | | | | |
|  | | | | | | | |
| CI: confidence interval; RR: risk ratio | | | | | | | |
|  | | | | | | | |
| ^a^ The risk with placebo has been calculated based on the cumulative placebo rates of all studies with a placebo arm.  ^b^The risk with treatment has been calculated by multiplying the risk with control with the RR (95% CI). If the calculation results in more than 1000 per 1000 people the number has been capped to 1000. Numbers have been rounded up to the closest whole number.  ^c^ The % risk difference has been calculated by subtracting the risk with control from the risk with treatment(95% CI) and dividing by 10. If the calculation results in more than 100% the number has been capped to 100%. Numbers have been rounded up to the closest whole number. | | | | | | | |

| **Sucra** | **Intervention (n=9)** | **network estimate RR** | **lower 95%CI** | **higher 95% CI** | **Number of direct studies** | **Direct GRADE** | **Reasons for direct downgrade** | **Indirect GRADE** | **Reasons for indirect downgrade** | **Network GRADE** | **Reasons for network downgrade** |
| --- | --- | --- | --- | --- | --- | --- | --- | --- | --- | --- | --- |
| **1** | Vitamin D | 0.67 | 0.15 | 3.08 | 1 | moderate | once due to rob | x | x | very low | twice due to imprecision |
| **2** | Adalimumab | 1.04 | 0.15 | 7.17 | 0 | x | x | moderate | once due to rob | very low | twice due to imprecision |
| **3** | Placebo |  |  |  |  |  |  |  |  |  |  |
| **4** | 5-ASA | 0.94 | 0.29 | 3.11 | 3 | moderate | once due to rob | moderate | once due to rob | very low | twice due to imprecision |
| **5** | Purine analogues | 1.12 | 0.32 | 3.99 | 2 | moderate | once due to rob | moderate | once due to rob | very low | twice due to imprecision |
| **6** | budesonide | 1.08 | 0.28 | 4.17 | 2 | low | twice due to rob | x | x | very low | twice due to imprecision |
| **7** | Purine analogues + enteral nutrition | 1.50 | 0.18 | 12.57 | 0 | x | x | moderate | once due to rob | very low | twice due to imprecision |
| **8** | Vedolizumab | 1.29 | 0.14 | 12.08 | 1 | moderate | once due to rob | x | x | very low | twice due to imprecision |
| **9** | Curcumin+purine analogues | 2.81 | 0.24 | 32.78 | 0 | x | x | moderate | once due to rob | very low | twice due to imprecision |

**Summary of Findings Tables and GRADE decisions (Total adverse events**)

| **Total adverse events** | | | | | | | |
| --- | --- | --- | --- | --- | --- | --- | --- |
| Patient or population: people with Crohn's disease post-surgery | | | | | | | |
| Settings: hospital setting | | | | | | | |
| Intervention: advanced therapies/purine analogues/ Purine analogues + enteral nutrition /5-ASA / antibiotics/ Curcumin + Purine analogues/vitamin D/budesonide | | | | | | | |
| Comparison: placebo | | | | | | | |
| **Treatment** | **Network evidence** | | **Anticipated absolute effects for network estimate** | | | **NNT (95% CI)** | **Notes** |
|  | **RR** | **Certainty** | **Risk with Placebo^a^** | **Risk with Agent^b^ (95% CI)** | **% Risk Difference with Agent^c^ (95% CI)** |  |  |
|  | **(95% CI)** |  |  |  |  |  |  |
| Adalimumab  (12-24 months) | 0.75 (0.49 to 1.16) | Very low | 441 per 1,000 | 331 per 1,000 (216 to 512) | 11% less (22.5% less to 7.1% more) | NA | The evidence is very uncertain |
|  |  |  |  |  |  |  |  |
|  |  | ⊕⊖⊖⊖ |  |  |  |  |  |
| Purine analogues + enteral nutrition  (12 months) | 0.87 (0.54 to 1.41) | Very low | 441 per 1,000 | 384 per 1,000 (238 to 622) | 1.3% more (12.8% less to 21.2% more) | NA | The evidence is very uncertain |
|  |  |  |  |  |  |  |  |
|  |  | ⊕⊖⊖⊖ |  |  |  |  |  |
| Purine analogues  (12-24 months) | 0.9 (0.67 to 1.2) | Very low | 441 per 1,000 | 397 per 1,000 (295 to 529) | 4.4% less (14.6% less to 8.8% more) | NA | The evidence is very uncertain |
|  |  | ⊕⊕⊖⊖ |  |  |  |  |  |
|  |  |  |  |  |  |  |  |
| Probiotics  (6-12 months) | 0.96 (0.41 to 2.24) | Very low | 441 per 1,000 | 423 per 1,000 (181 to 988) | 1.8% less (26% less to 54.7% more) | NA | The evidence is very uncertain |
|  |  |  |  |  |  |  |  |
|  |  | ⊕⊖⊖⊖ |  |  |  |  |  |
| 5-ASA  (12-72 months) | 0.97 (0.74 to 1.26) | Very low | 441 per 1,000 | 428 per 1,000 (326 to 556) | 1.3% less (11.5% less to 11.5% more) | NA | The evidence is very uncertain |
|  |  | ⊕⊖⊖⊖ |  |  |  |  |  |
|  |  |  |  |  |  |  |  |
| Budesonide  (12 months) | 1.03 (0.71 to 1.48) | Very low | 441 per 1,000 | 454 per 1,000 (313 to 653) | 1.3% more (12.8% less to 21.2% more) | NA | The evidence is very uncertain |
|  |  |  |  |  |  |  |  |
|  |  | ⊕⊖⊖⊖ |  |  |  |  |  |
| Vitamin D  (6 months) | 1.1 (0.74 to 1.62) | Very low | 441 per 1,000 | 485 per 1,000 (326 to 714) | 4.4% more (11.5% less to 27.3% more) | NA | The evidence is very uncertain |
|  |  |  |  |  |  |  |  |
|  |  | ⊕⊖⊖⊖ |  |  |  |  |  |
| Infliximab  (12-24 months) | 1.56 (0.71 to 3.41) | Very low | 441 per 1,000 | 688 per 1,000 (313 to 1000) | 24.7% more (12.8% less to 55.9% more) | NA | The evidence is very uncertain |
|  |  |  |  |  |  |  |  |
|  |  | ⊕⊖⊖⊖ |  |  |  |  |  |
| Antibiotics  (6-12 months) | 1.81 (0.99 to 3.29) | Very Low | 441 per 1,000 | 798 per 1,000 (437 to 1000) | 35.7% more (0.4% less to 55.9% more) | NA | The evidence is very uncertain |
|  |  | ⊕⊖⊖⊖ |  |  |  |  |  |
|  |  |  |  |  |  |  |  |
|  |  |  |  |  |  |  |  |
|  |  |  |  |  |  |  |  |
| **GRADE Working Group grades of evidence** | | | | | | | |
| **High certainty**: we are very confident that the true effect lies close to that of the estimate of the effect. | | | | | | | |
| **Moderate certainty**: we are moderately confident in the effect estimate; the true effect is likely to be close to the estimate of the effect, but there is a possibility that it is substantially different. | | | | | | | |
| **Low certainty**: our confidence in the effect estimate is limited; the true effect may be substantially different from the estimate of the effect. | | | | | | | |
| **Very low certainty**: we have very little confidence in the effect estimate; the true effect is likely to be substantially different from the estimate of effect. | | | | | | | |
|  | | | | | | | |
| **CI: confidence interval; RR: risk ratio** | | | | | | | |
|  | | | | | | | |
| ^a^ The risk with placebo has been calculated based on the cumulative placebo rates of all studies with a placebo arm.  ^b^The risk with treatment has been calculated by multiplying the risk with control with the RR (95% CI). If the calculation results in more than 1000 per 1000 people the number has been capped to 1000. Numbers have been rounded up to the closest whole number.  ^c^ The % risk difference has been calculated by subtracting the risk with control from the risk with treatment(95% CI) and dividing by 10. If the calculation results in more than 100% the number has been capped to 100%. Numbers have been rounded up to the closest whole number. | | | | | | | |

| **Sucra** | **Intervention (n=10)** | **network estimate RR** | **lower 95%CI** | **higher 95% CI** | **Number of direct studies** | **Direct GRADE** | **Reasons for direct downgrade** | **Indirect GRADE** | **Reasons for indirect downgrade** | **Network GRADE** | **Reasons for network downgrade** |
| --- | --- | --- | --- | --- | --- | --- | --- | --- | --- | --- | --- |
| 1 | Adalimumab | 0.75 | 0.49 | 1.16 | 0 | x | x | moderate | once due to rob in the strongest loop | very low | twice due to imprecision |
| 2 | Purine analogues + enteral nutrition | 0.87 | 0.54 | 1.41 | 0 | x | x | x | x | very low | once due to imprecision |
| 3 | Purine analogues | 0.9 | 0.67 | 1.2 | 2 | low | once due to rob, once due to inconsistency | moderate | once due to rob in the strongest loop | very low | twice due to imprecision |
| 4 | Probiotics | 0.96 | 0.41 | 2.24 | 2 | low | once due to rob, once due to inconsistency | x | x | very low | twice due to imprecision |
| 5 | 5-ASA | 0.97 | 0.74 | 1.26 | 5 | moderate | once due to rob | moderate | once due to rob in the strongest loop | very low | twice due to imprecision |
| 6 | Budesonide | 1.03 | 0.71 | 1.48 | 2 | low | once due to rob, once due to inconsistency | x | x | very low | twice due to imprecision, once due to incoherence |
| 7 | placebo |  |  |  |  |  |  |  |  |  |  |
| 8 | Vitamin D | 1.1 | 0.74 | 1.62 | 1 | moderate | once due to rob | moderate | once due to rob in the strongest loop | very low | twice due to imprecision, once due to incoherence |
| 9 | Infliximab | 1.56 | 0.71 | 3.41 | 2 | moderate | once due to rob | x | x |  |  |
| 10 | Antibiotics | 1.81 | 0.99 | 3.29 | 2 | low | once due to rob, once due to inconsistency | x | x | very low | twice due to imprecision |

# **eFigures1**. Network plots

**Network plot for clinical relapse**

**
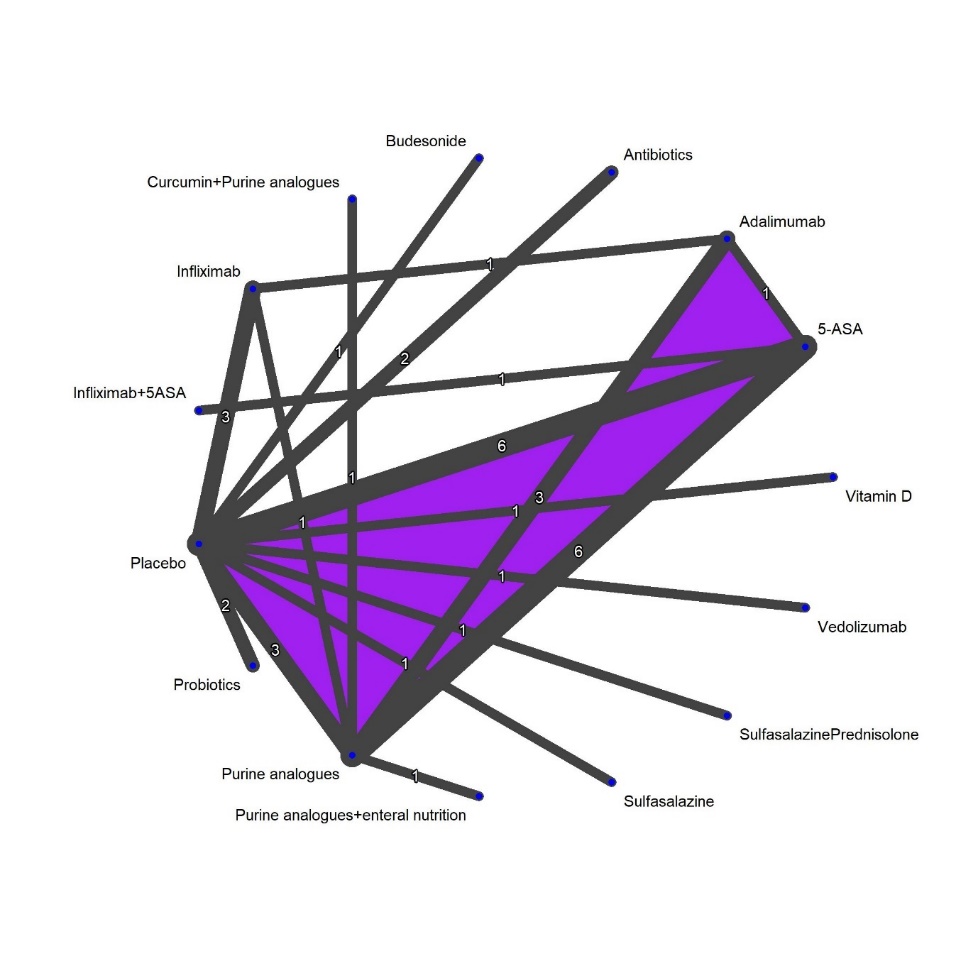
**

**Network plot for induction of Endoscopic relapse.**

**
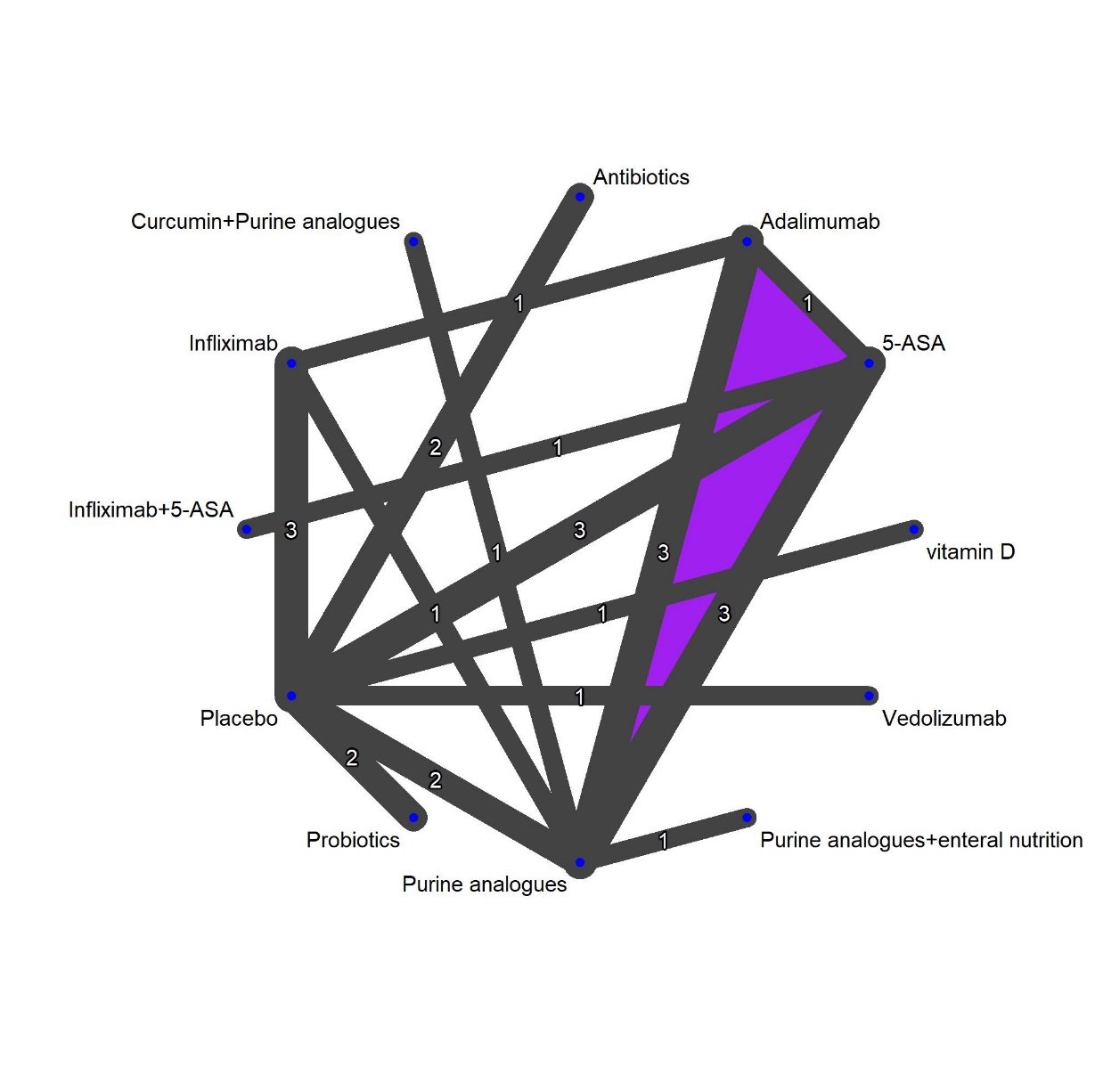
**

**Network plot for withdrawals due to adverse events**


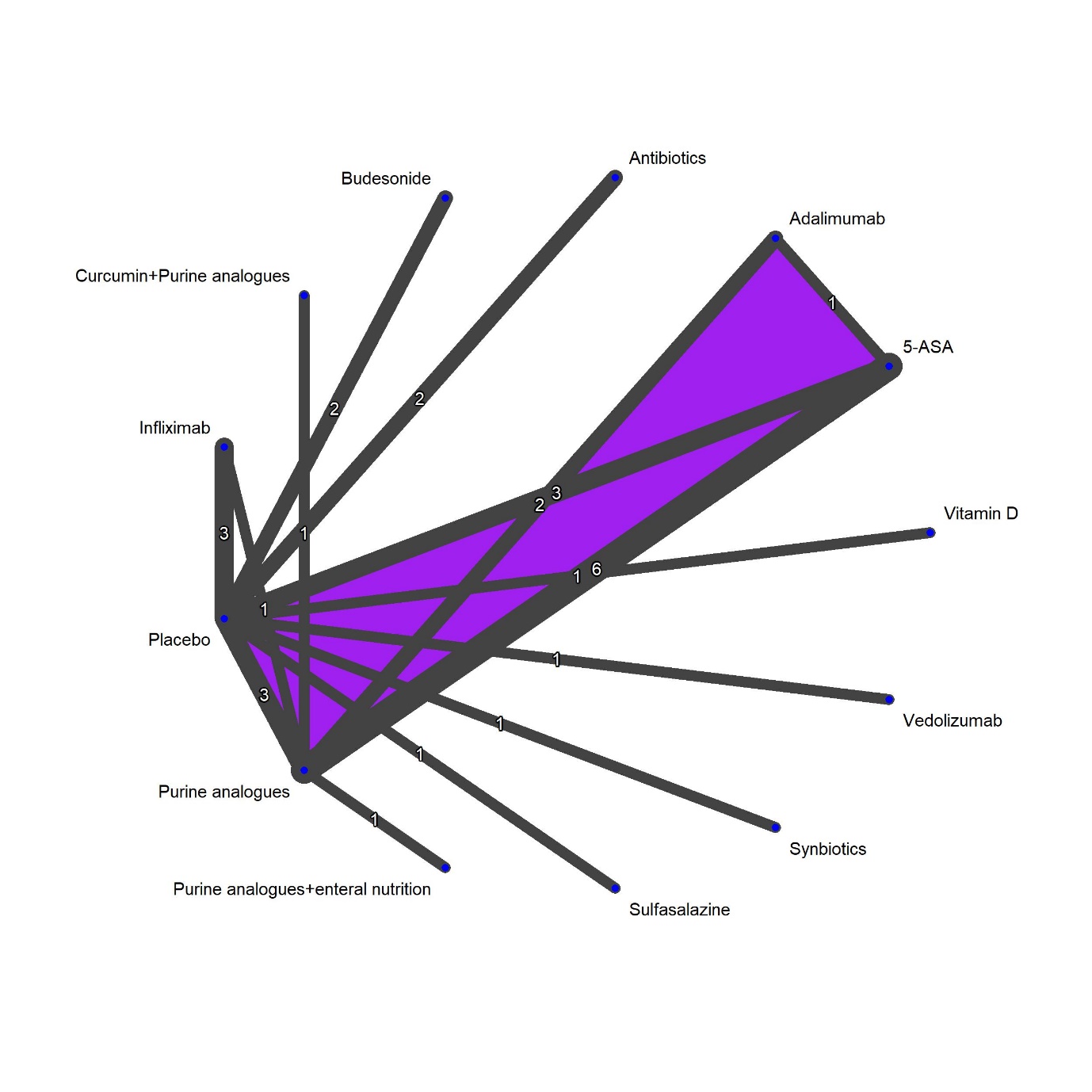


**Network plot for serious adverse events**


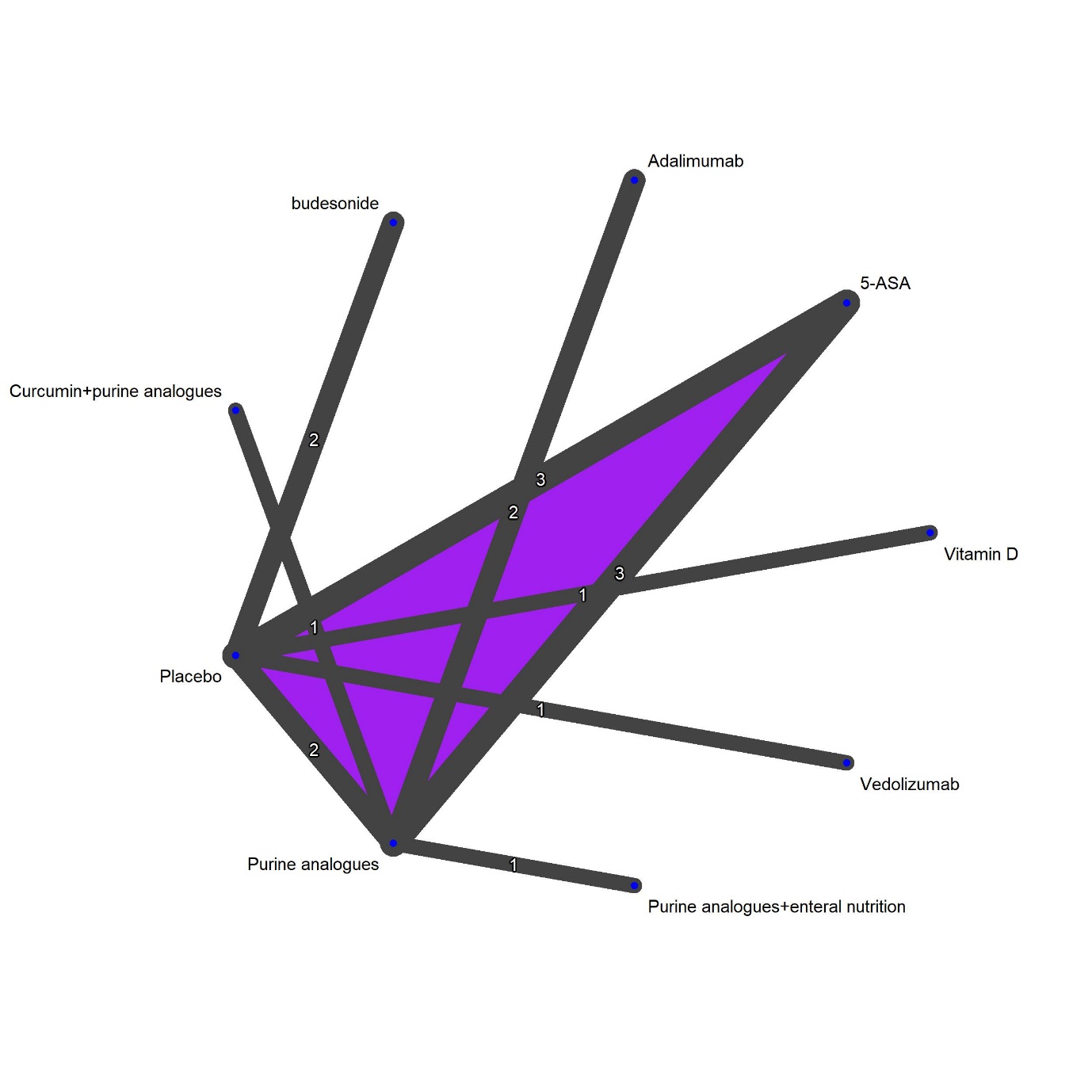


**Network plot for induction of total adverse events**


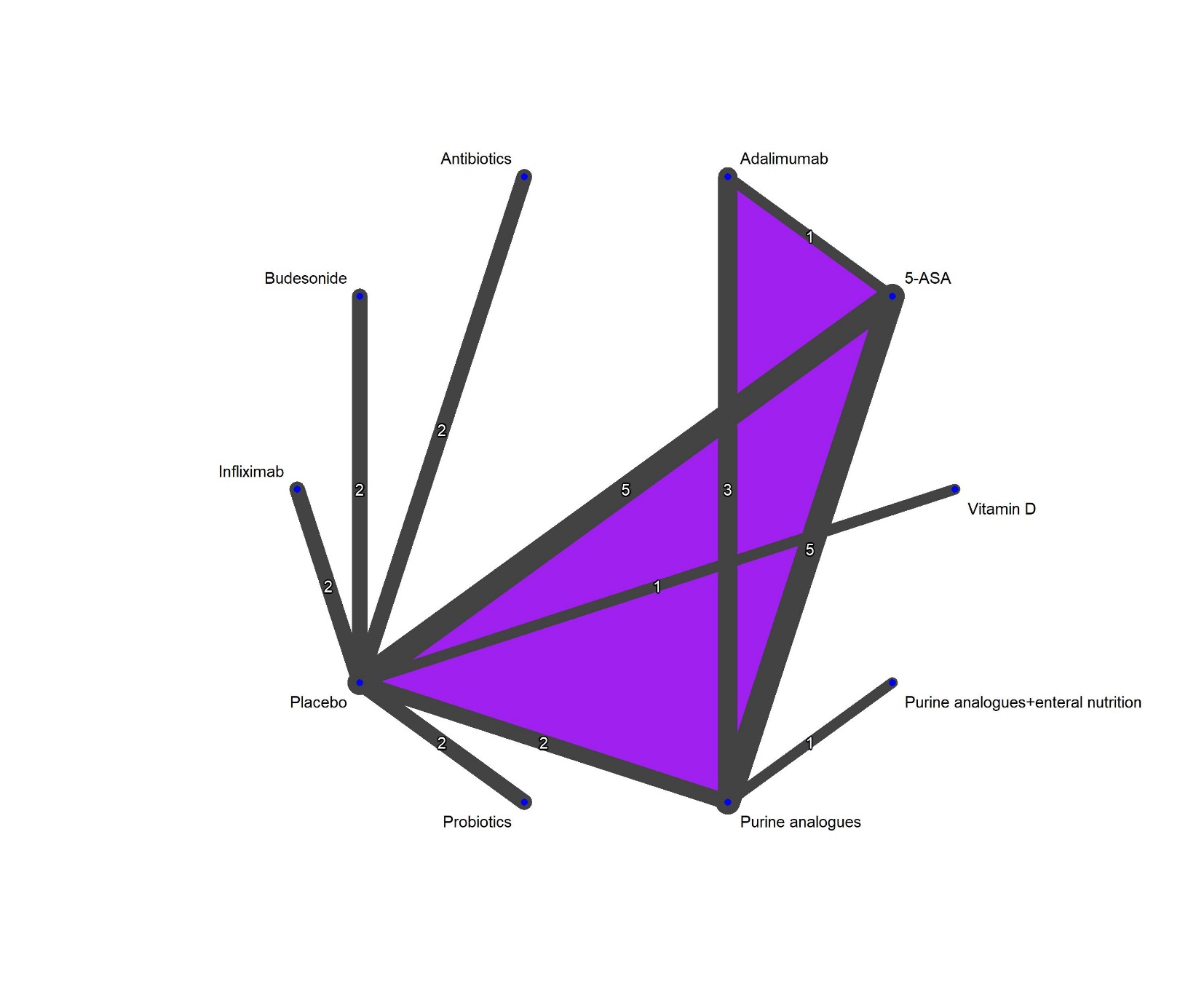


# **eFigures** 2. NETWORK FOREST PLOTS, SUCRA PROBABILITIES, AND DIRECT/INDIRECT/NETWORK ESTIMATES FOREST PLOTS

**Network forest plots Clinical Relapse**


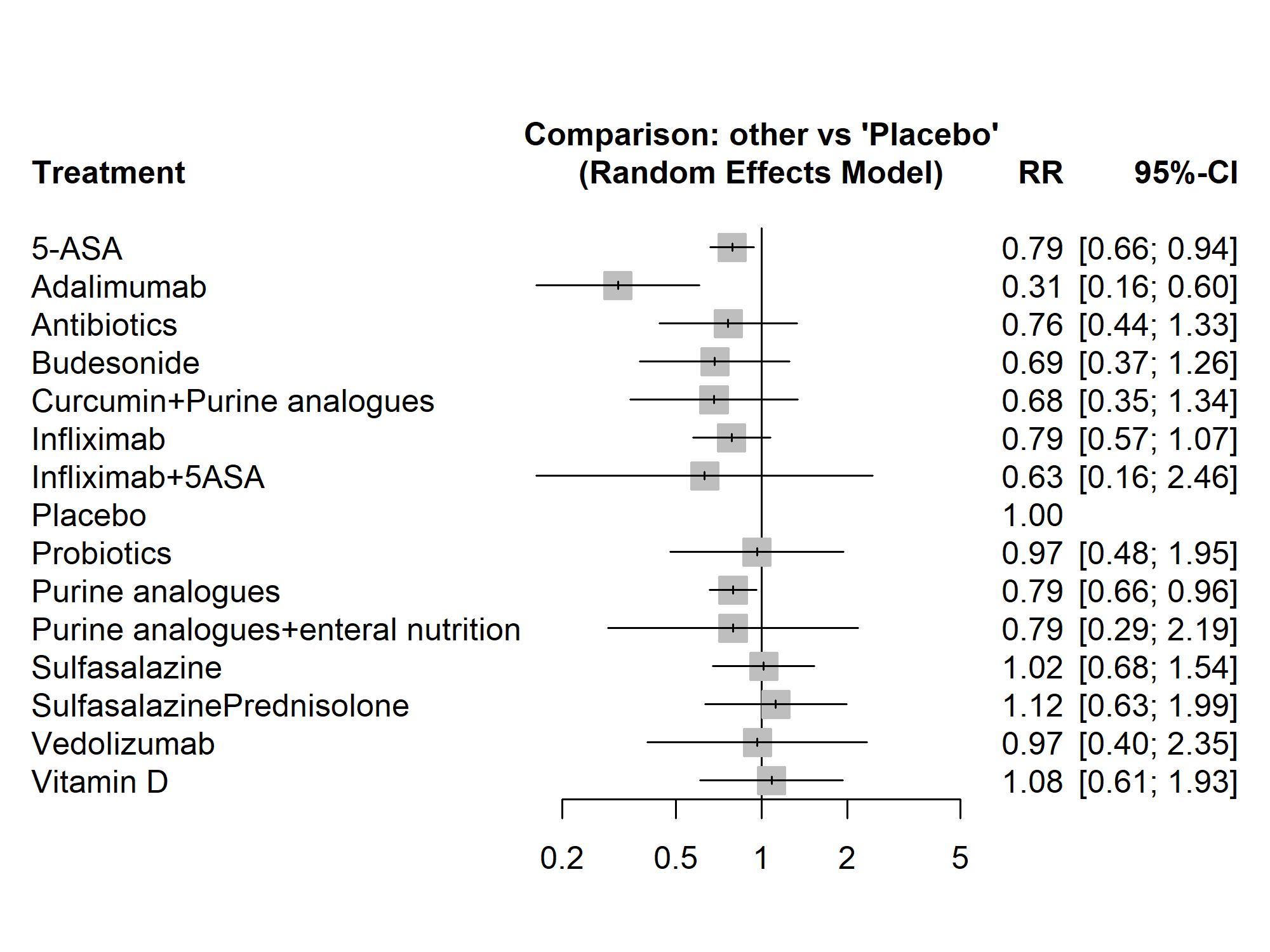


**SUCRA probabilities Clinical Relapse**


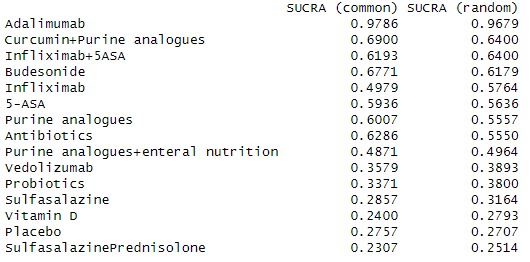


**Direct/indirect/network estimates forest plots Clinical Relapse**

**
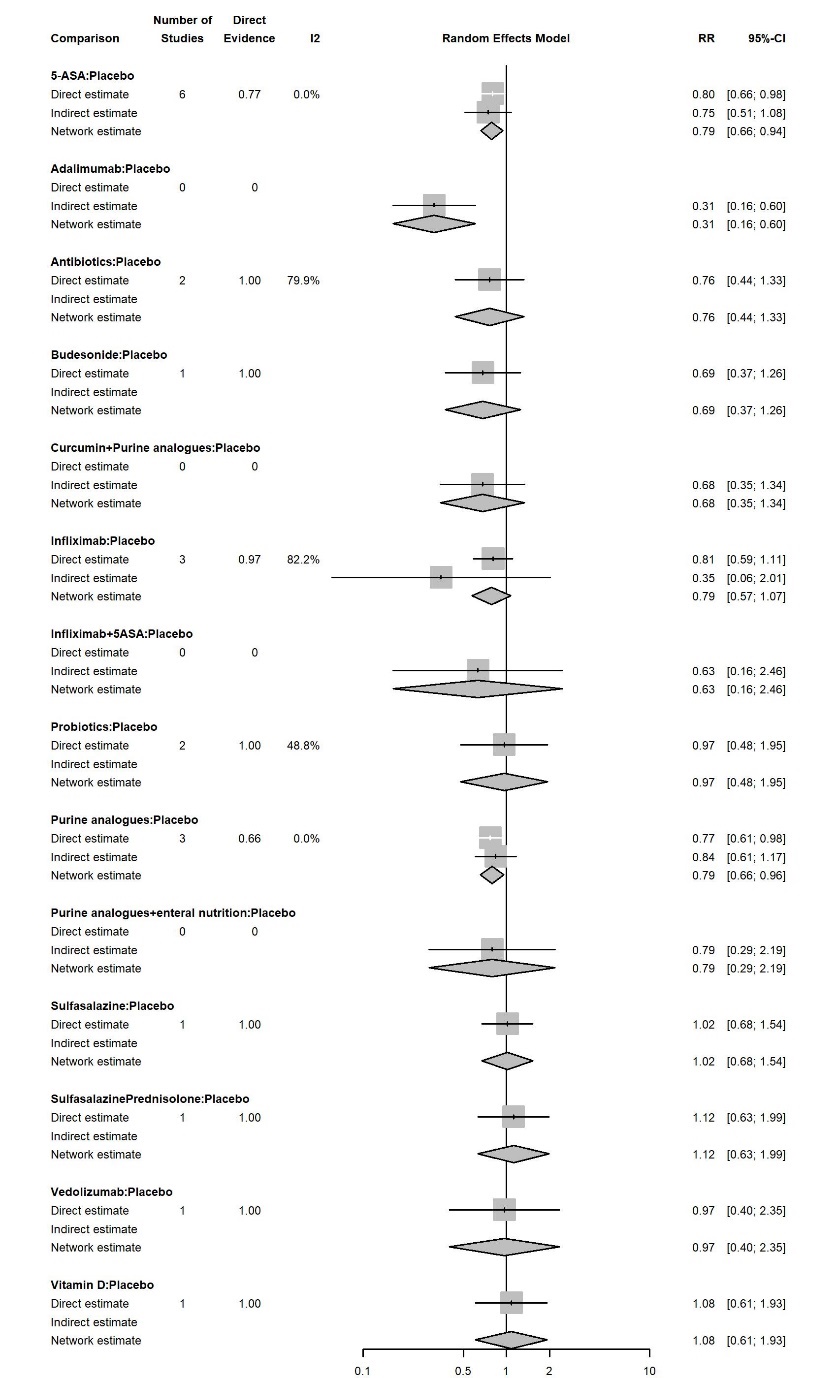
**

**Network forest plots Endoscopic Relapse**

**
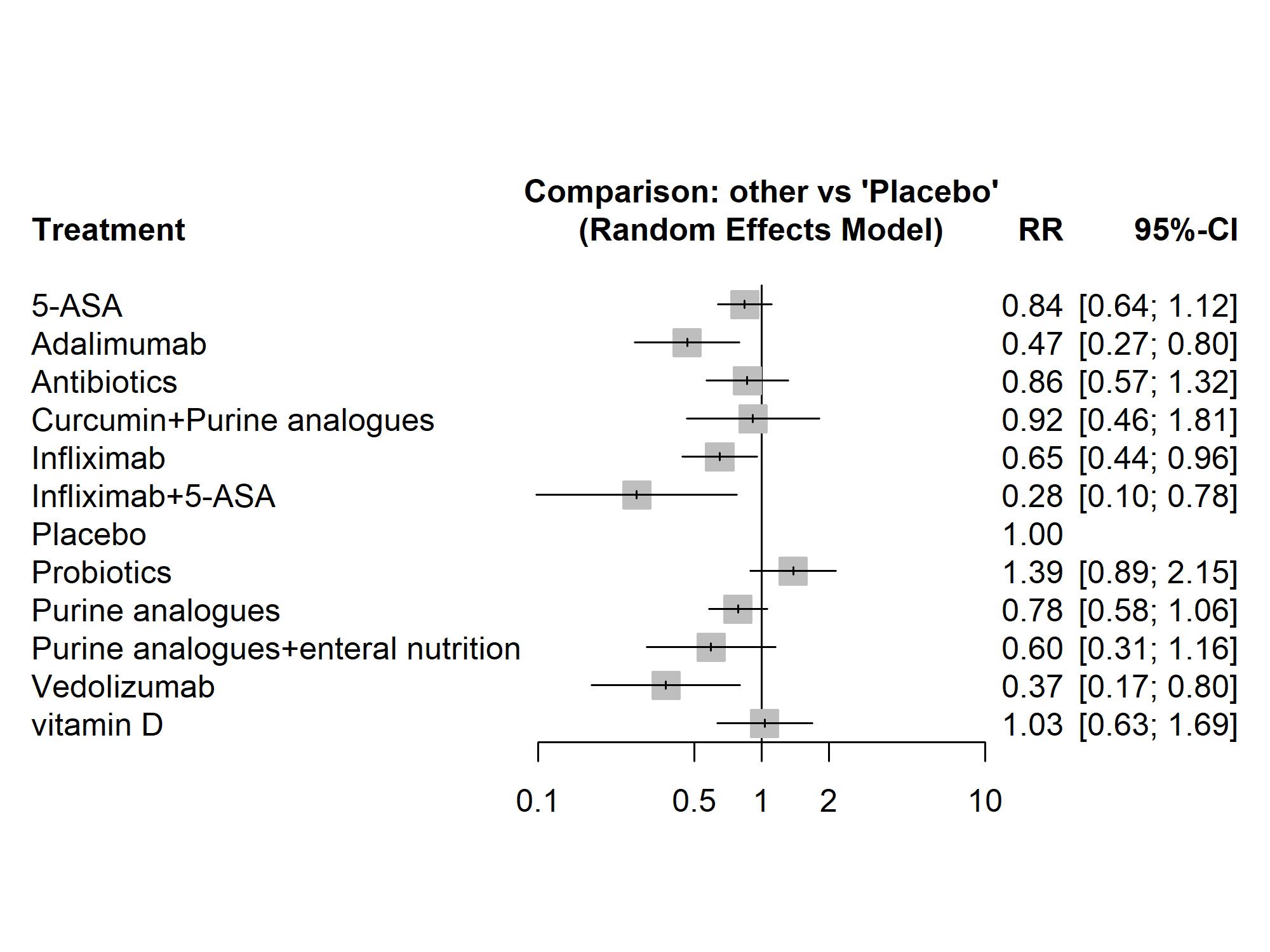
**

**SUCRA probabilities Endoscopic Relapse**

**
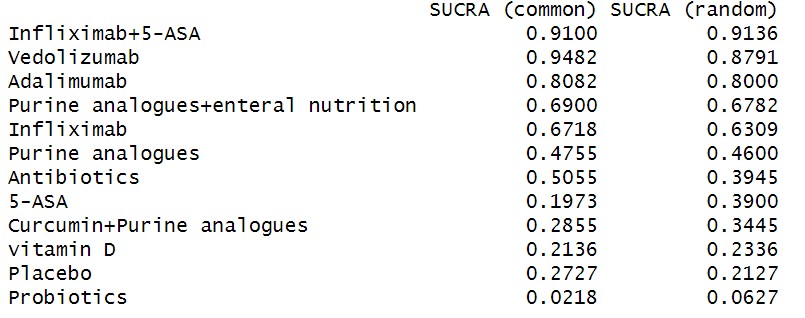
**

**Direct/indirect/network estimates forest plots Endoscopic Relapse**

**
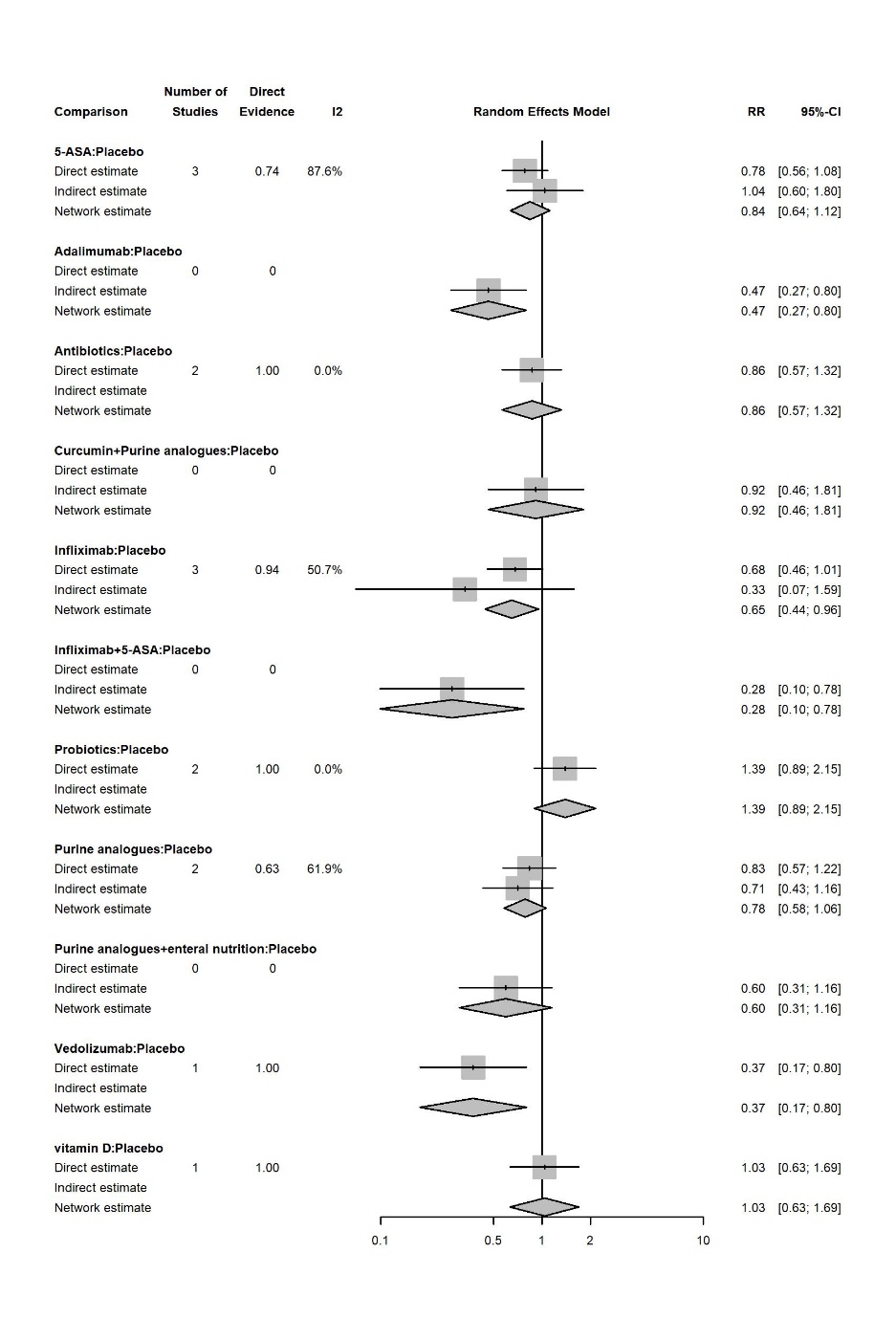
**

**Network forest plots Withdrawals due to adverse events**


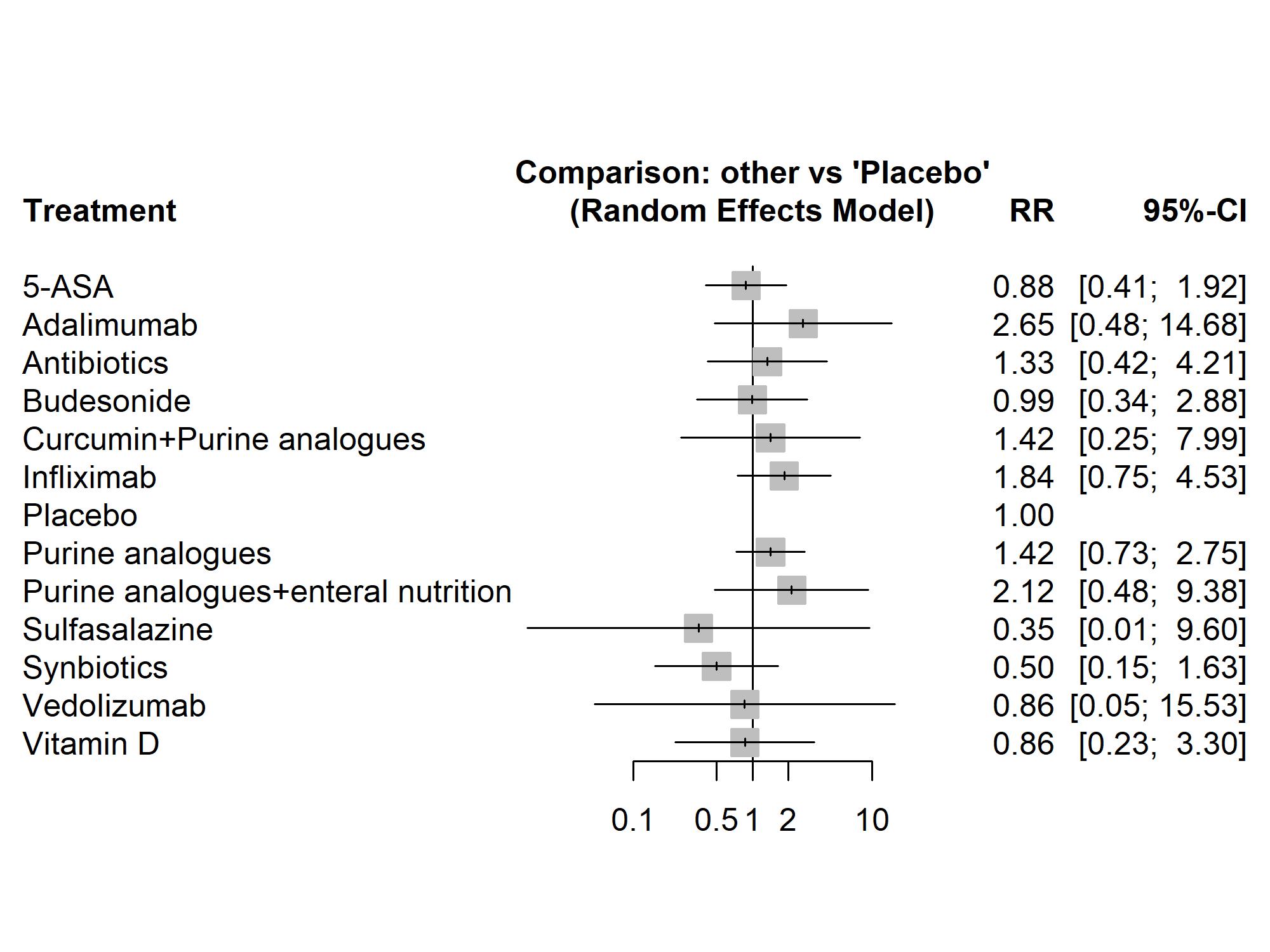


**SUCRA probabilities Withdrawals due to adverse events**


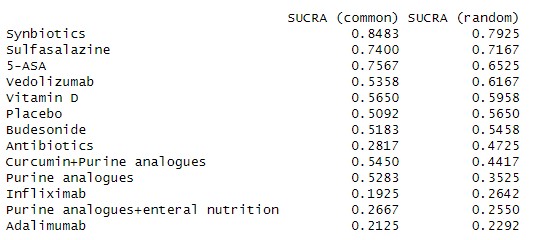


**Direct/indirect/network estimates forest plots WAE**


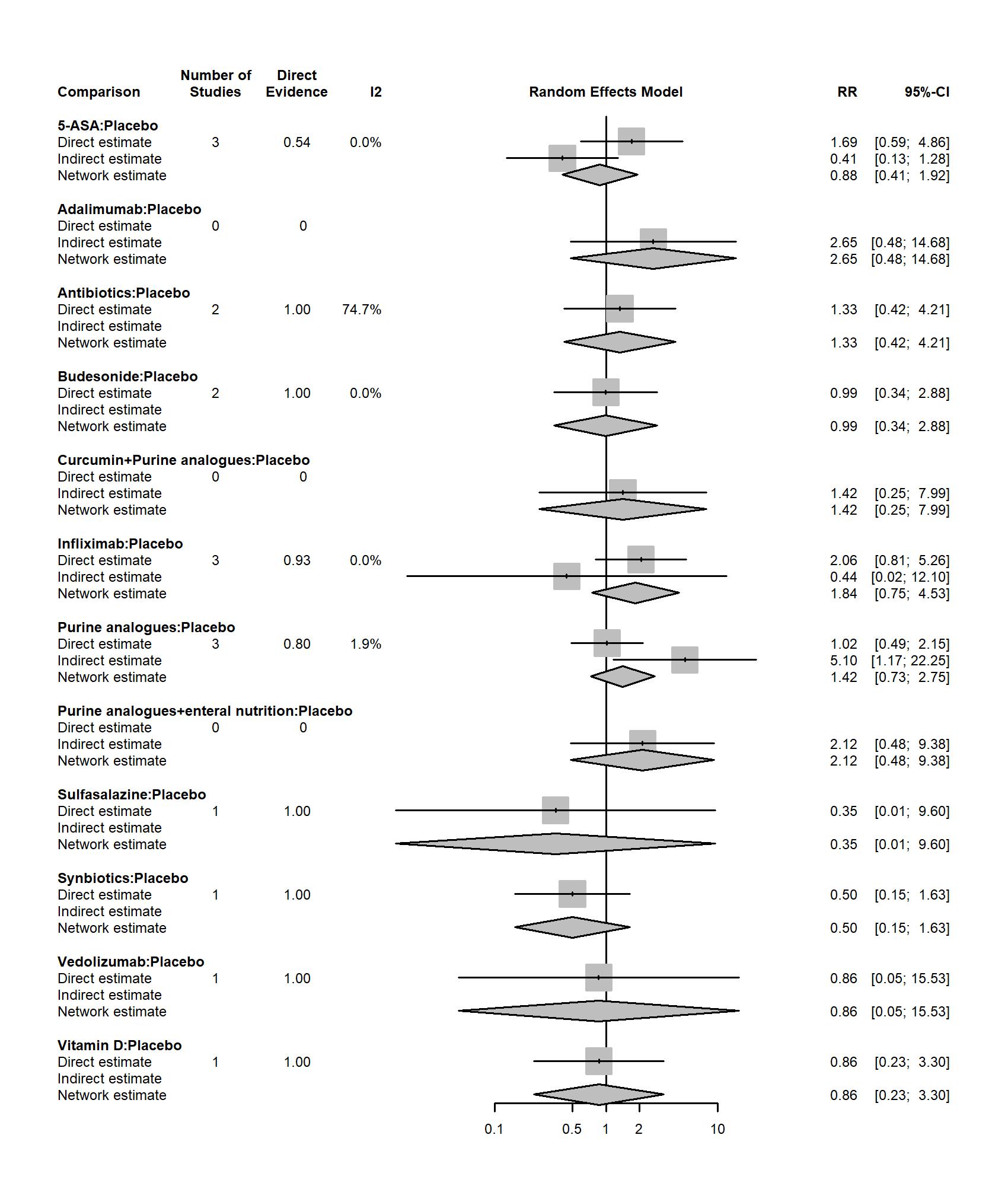


**Network forest plots for Serious Adverse Events**


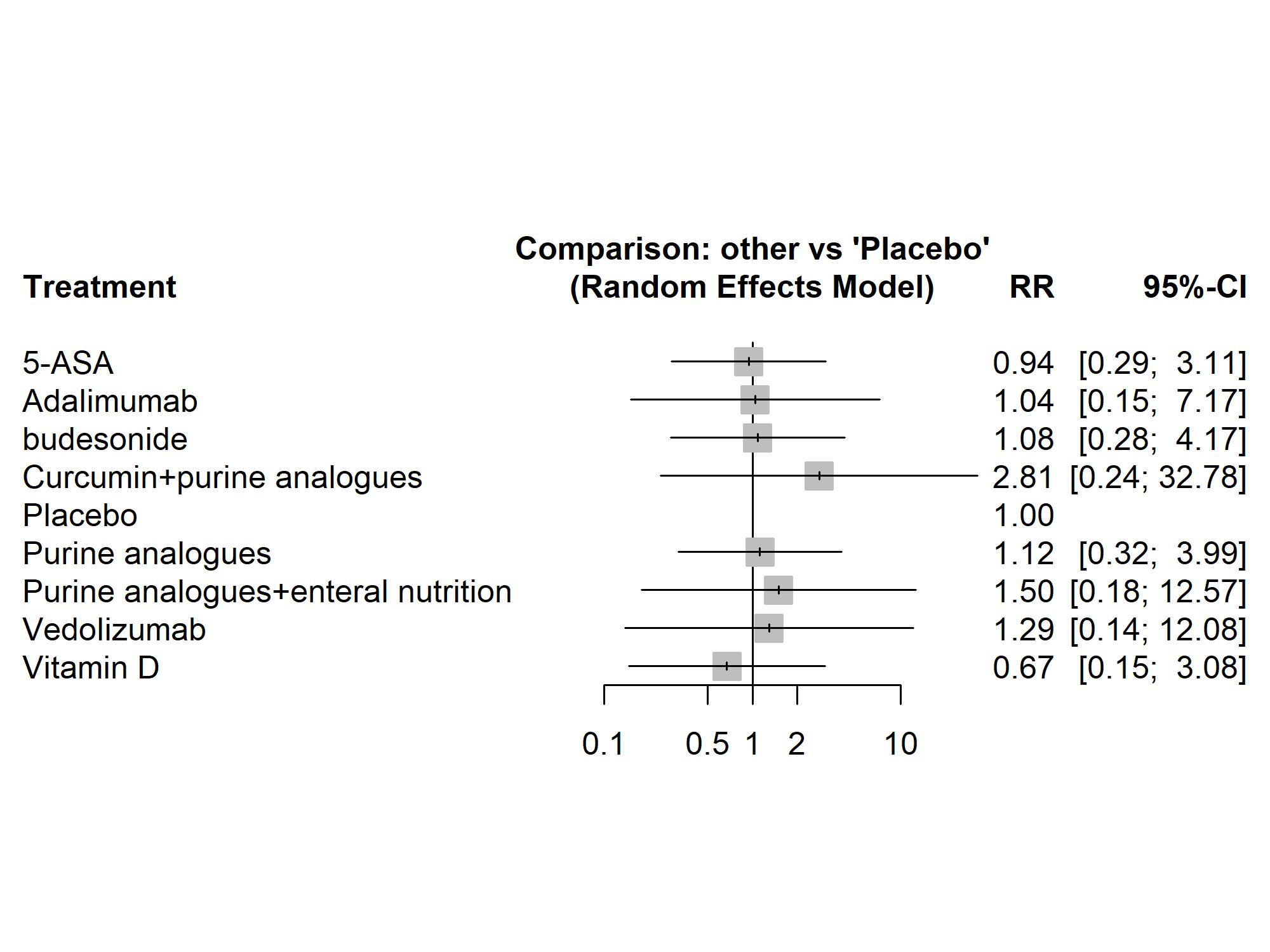


**SUCRA probabilities for Serious Adverse Events**


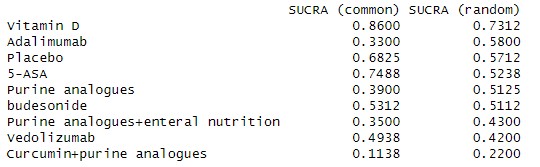


**Direct/indirect/network estimates forest plots for serious Adverse Events**


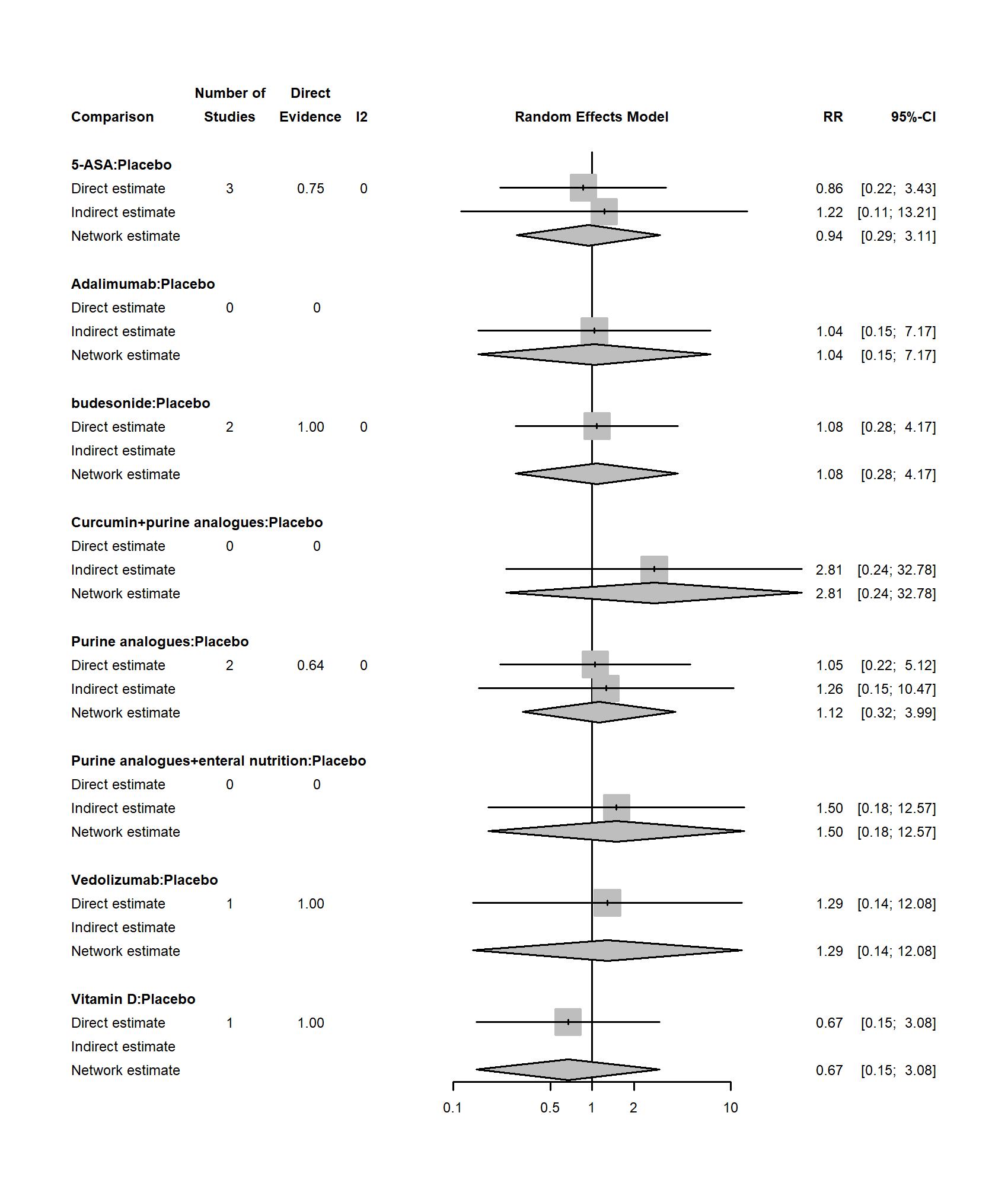


**Network forest plots Total Adverse Events**


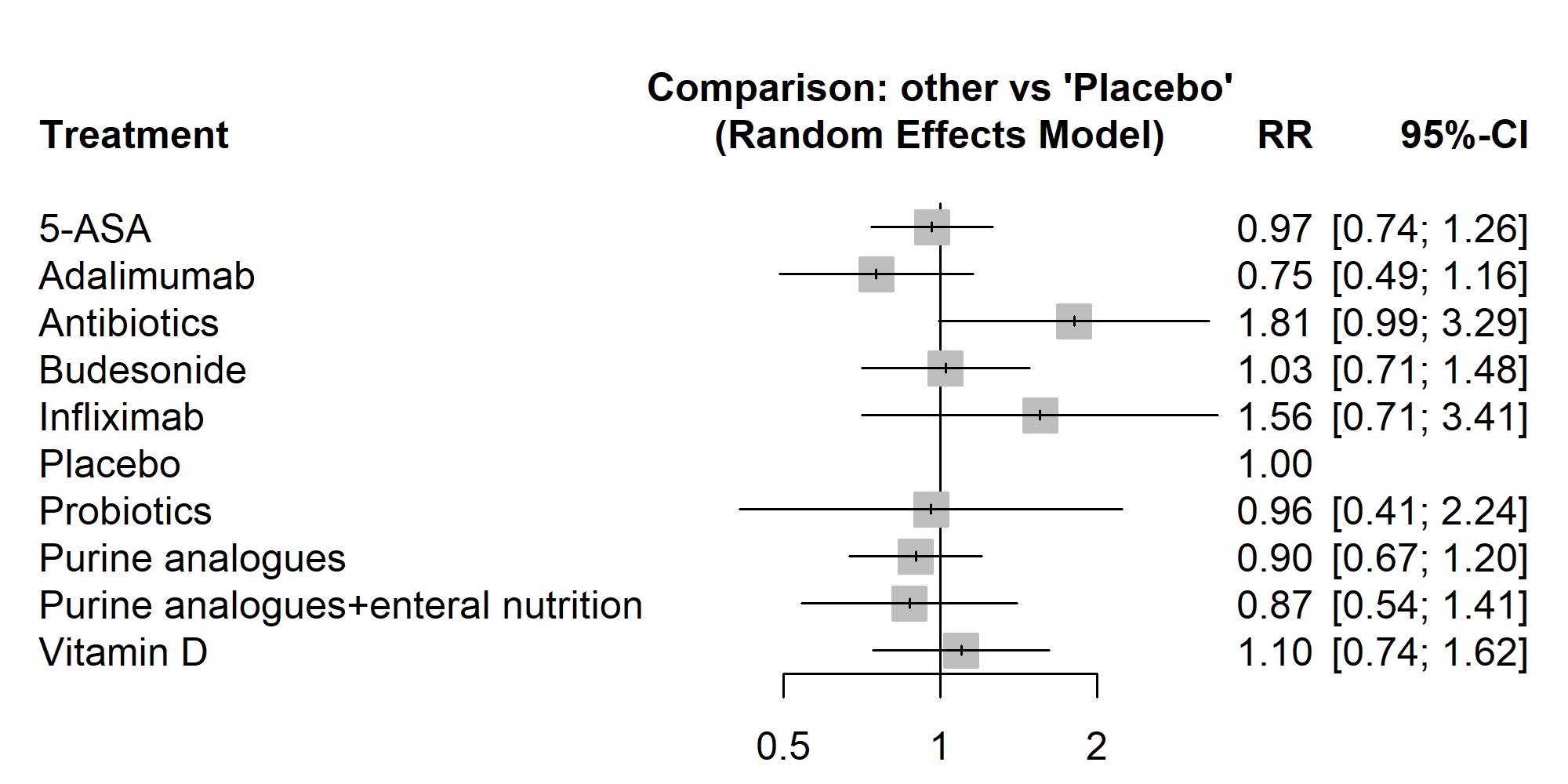


**SUCRA probabilities Total Adverse Events**


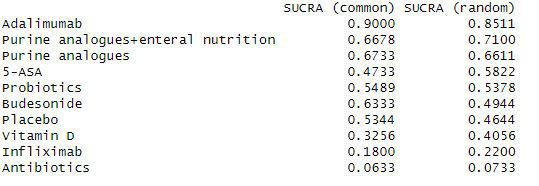


**Direct/indirect/network estimates forest plots Total Adverse Events**


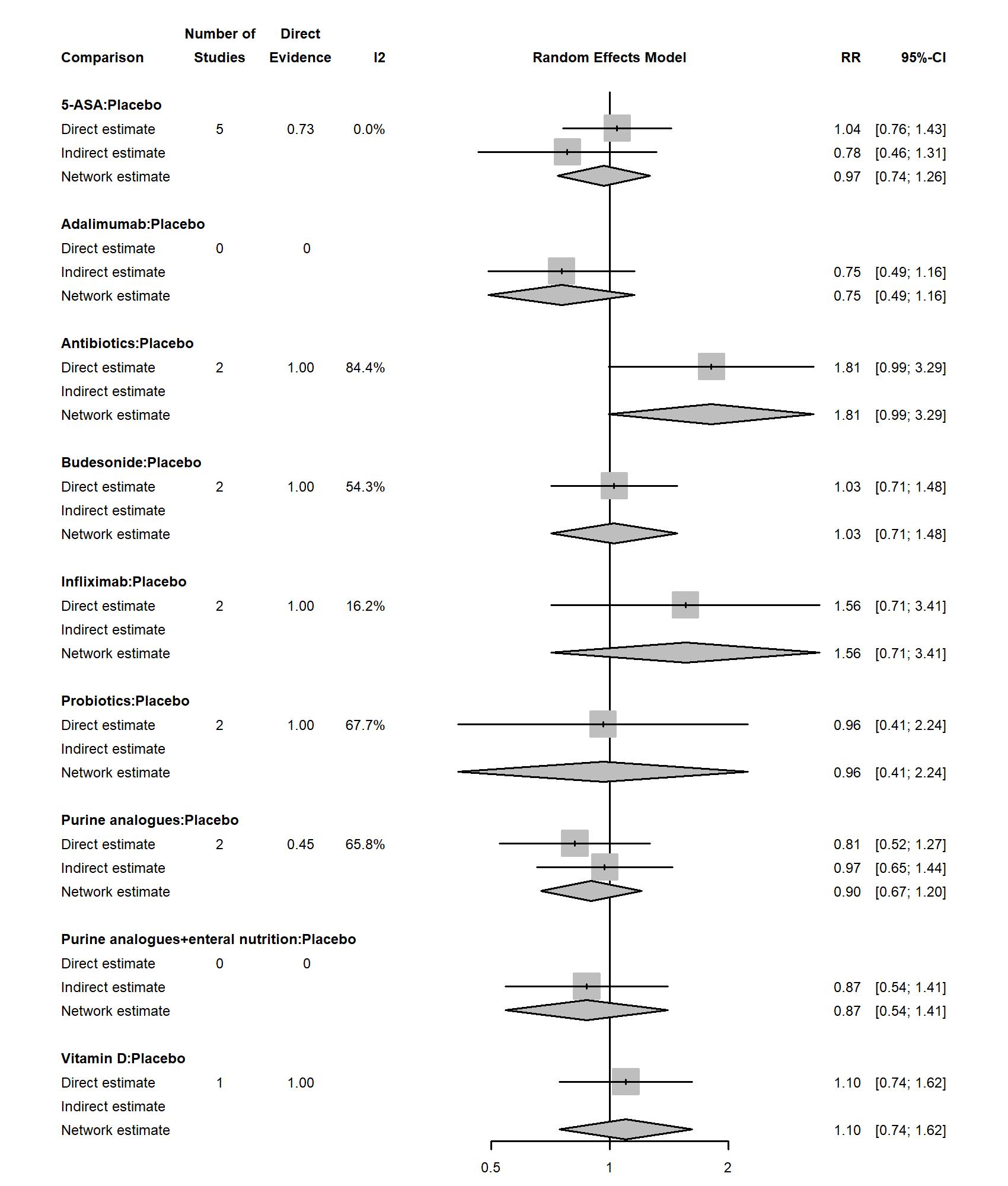


# **eFigures 3**. SUBGROUP AND SENSITIVITY ANALYSES

**CLINICAL RELAPSE**

**Subgroup analysis for studies with follow-up period ≤ 12 months**


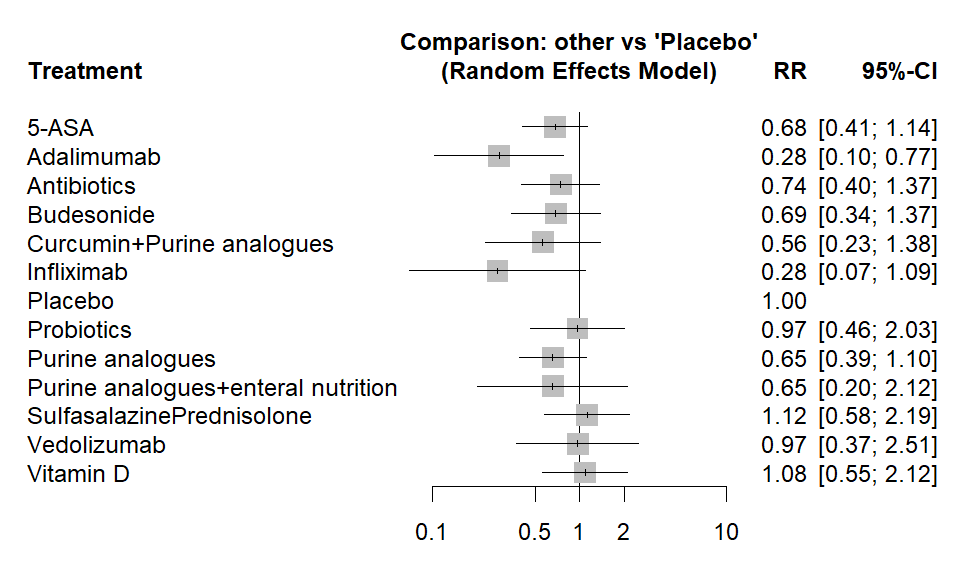


**Subgroup analysis for studies with follow-up period > 12 months**


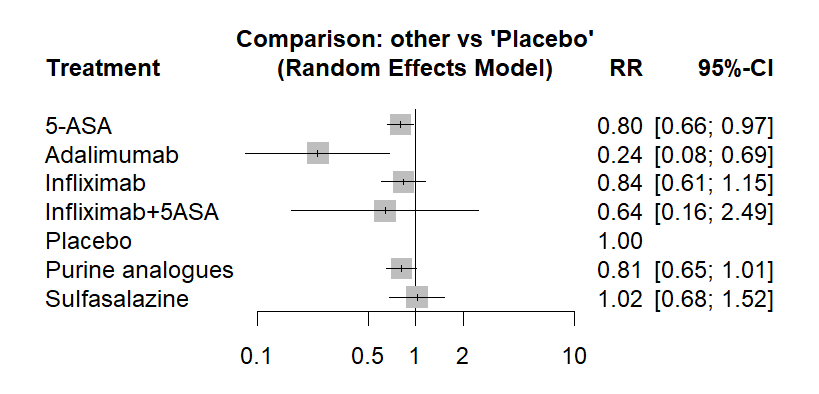


**Sensitivity analysis excluding studies with only high-risk patients**


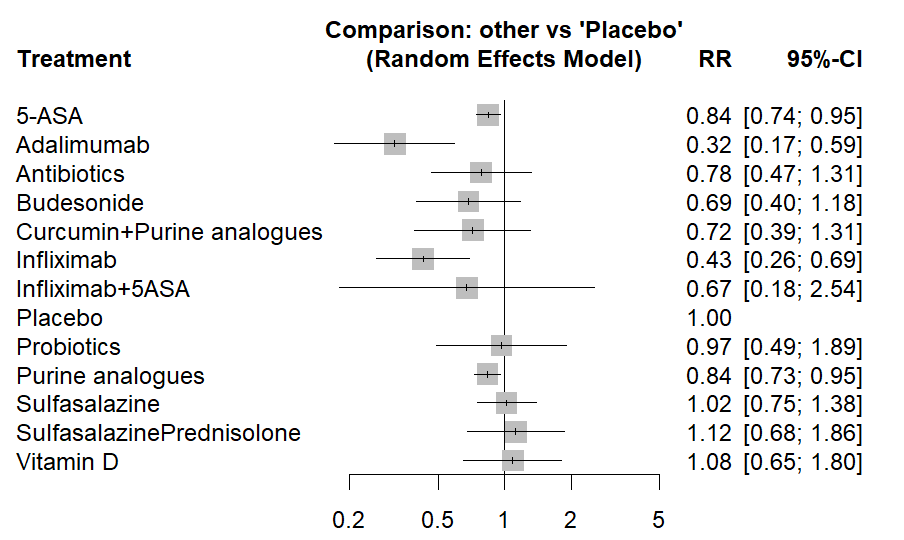


**Sensitivity analysis for studies using CDAI as definition of clinical relapse**


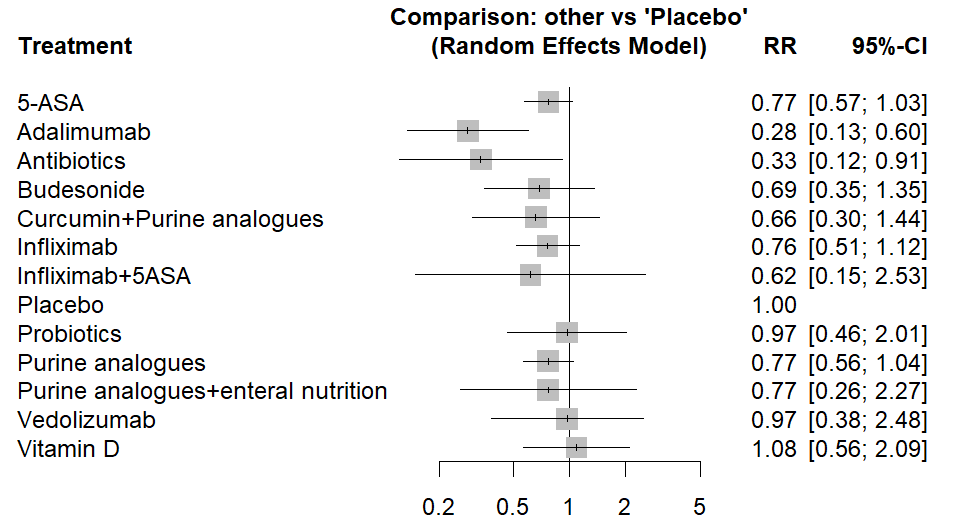


**Sensitivity analysis excluding studies that enrolled patients with clinical relapse at baseline**


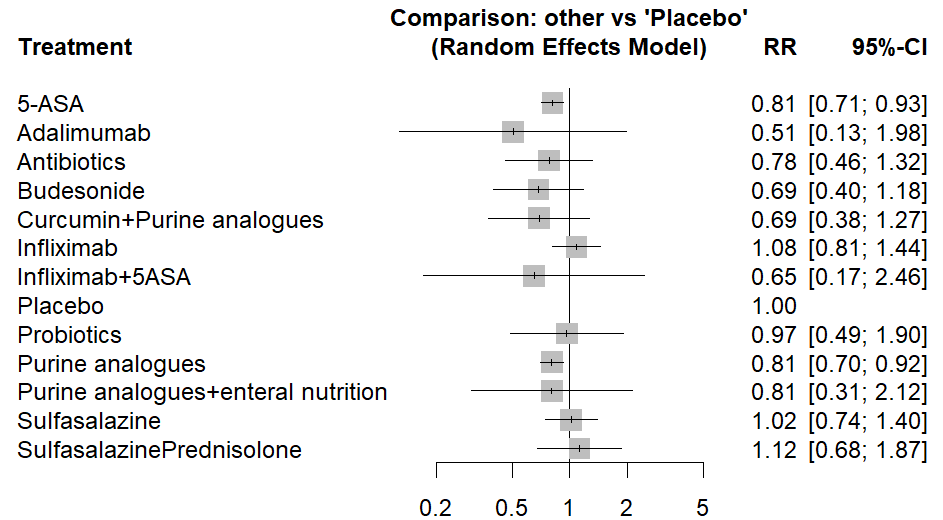


**Sensitivity analysis excluding studies with concurrent antibiotic use**


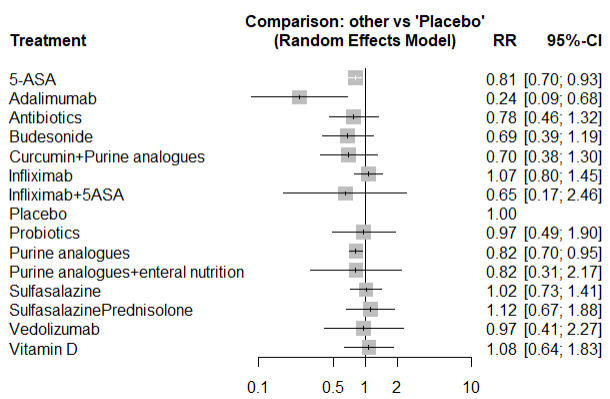


**SUBGROUP AND SENSITIVITY ANALYSIS ENDOSCOPIC RELAPSE**

**Subgroup analysis for studies with follow-up period ≤ 12 months on endoscopic relapse**


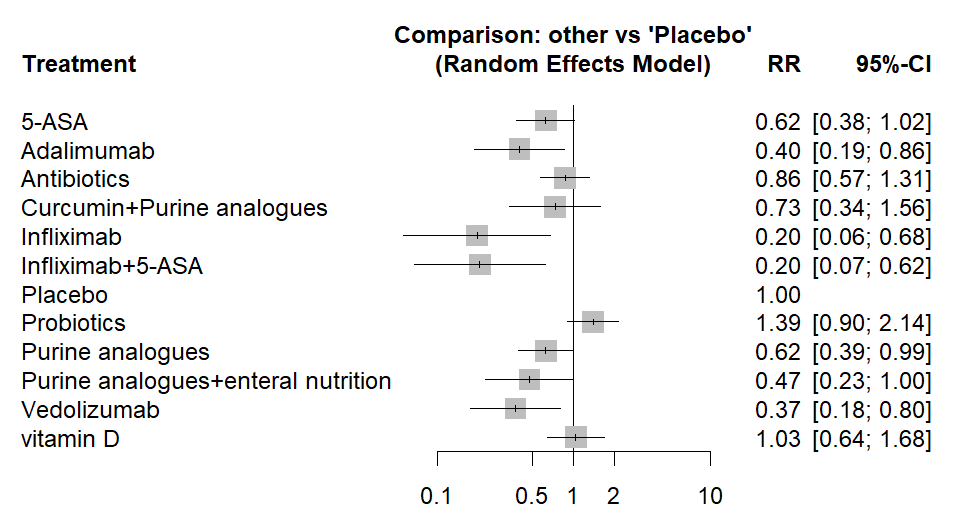


**Subgroup analysis for studies with follow-up period > 12 months on endoscopic relapse**


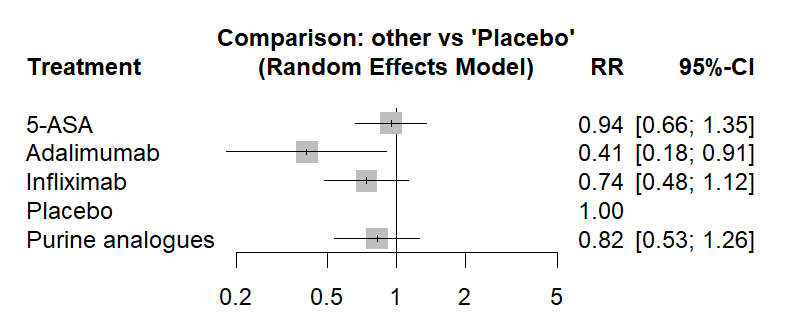


**Sensitivity analysis excluding studies with only high-risk patients**


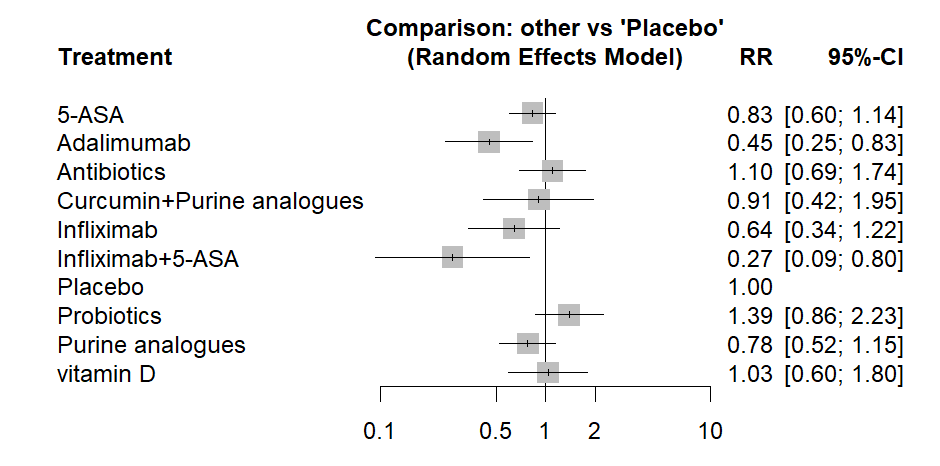


**Sensitivity analysis excluding studies that enrolled patients with endoscopic relapse at baseline**


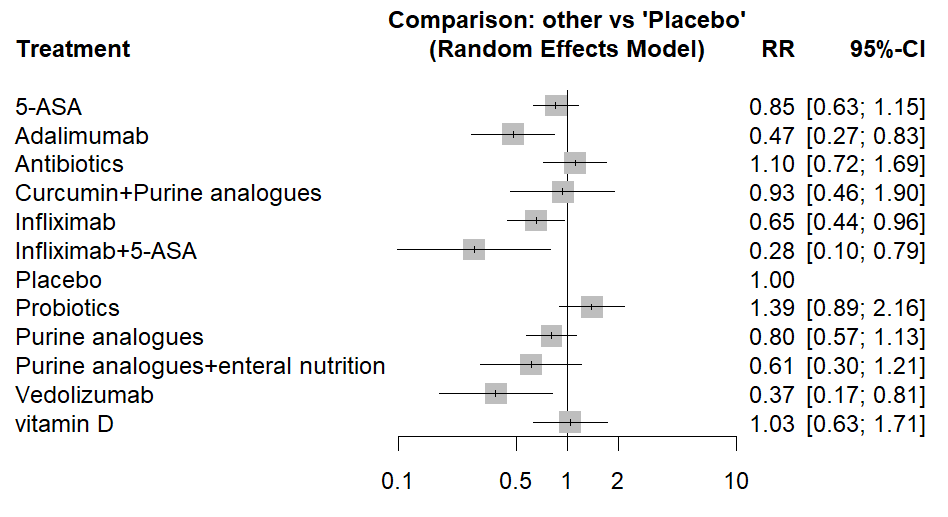


**Sensitivity analysis excluding studies with concurrent antibiotic use**


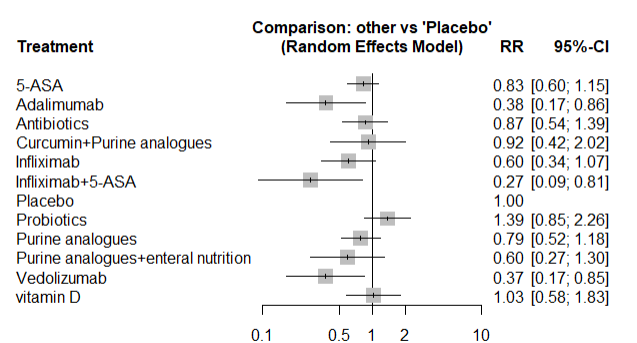


# **eFigure 4.** Risk of bias summary


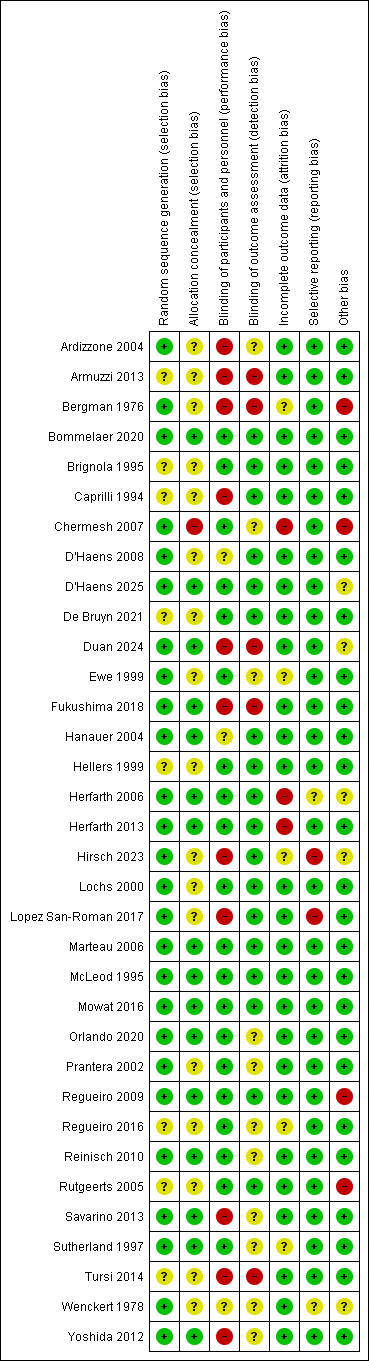


# **eAppendix 1**. Supporting Judgements for Risk of Bias Assessment

| Ardizzone 2004 | | |
| --- | --- | --- |
| Bias | Authors' judgement | Support for judgement |
| Random sequence generation (selection bias) | Low risk | Quote: “After surgery, participants who met the inclusion criteria and who agreed to enter the study were randomised to receive mesalamine or AZA by a computer-generated list” and “Randomization was performed in blocks of 10”. Comment: computer-generated block randomisation. |
| Allocation concealment (selection bias) | Unclear risk | Insufficient information to make judgement. |
| Blinding of participants and personnel (performance bias) – All outcomes | High risk | Comment: the study is open-label and blinding is not performed. |
| Blinding of outcome assessment (detection bias) – All outcomes | Unclear risk | Insufficient information to make judgement, however it is unlikely |
| Incomplete outcome data (attrition bias) | Low risk | Quotes: “In the intention-to-treat analysis, all randomised participants who received at least one dose of the study drug and were subjected to the baseline evaluation were considered for the analysis.” and “Outcome measures were analysed in all randomised participants who had taken at least one dose of the study medication (intention-to-treat population)…”. Comment: withdrawals were low and balanced across groups. |
| Selective reporting (reporting bias) | Low risk | Trial registration not available; however, all outcomes stated in the method section were assessed and reported. |
| Other bias | Low risk | Quote: “No significant differences were observed between the 2 treatment groups regarding age, sex, duration of disease, location of disease, fistula and abscess at surgery, surgical procedure, previous operations, and CD therapy during the previous 6 months”. Comment: baseline characteristics well balanced across groups. |

| Armuzzi 2013 | | |
| --- | --- | --- |
| Bias | Authors' judgement | Support for judgement |
| Random sequence generation (selection bias) | Unclear risk | Quote: “Subjects were randomised with a simple unblinded 1:1 allocation ratio to receive…”. Comment: simple randomisation performed, however insufficient information on the method of randomisation used. |
| Allocation concealment (selection bias) | Unclear risk | Not stated. |
| Blinding of participants and personnel (performance bias) – All outcomes | High risk | Open-label study. |
| Blinding of outcome assessment (detection bias) – All outcomes | High risk | Quote: “One unblinded endoscopist (AP) did all the examinations and calculated scores. Two further unblinded endoscopists (IDV and GA) separately reviewed videos and in case of discordance a consensus agreement was reached among the three operators.” Comment: blinding of outcome assessors not performed. |
| Incomplete outcome data (attrition bias) – All outcomes | Low risk | Quote: “One patient did not tolerate azathioprine because of severe nausea with epigastric pain and withdrew from the study after 5 weeks of treatment”. Comment: only one participant withdrew from the study and reason described. |
| Selective reporting (reporting bias) | Low risk | All outcome data stated in the method section were reported. |
| Other bias | Low risk | Groups well balanced at baseline and no other apparent sources of bias detected. |

| Bergman 1976 | | |
| --- | --- | --- |
| Bias | Authors' judgement | Support for judgement |
| Random sequence generation (selection bias) | Low risk | Quote: “...groups assigned by drawing a lot”. Comment: simple randomisation. |
| Allocation concealment (selection bias) | Unclear risk | Insufficient information to make judgement. |
| Blinding of participants and personnel (performance bias) – All outcomes | High risk | Insufficient information provided, but blinding very unlikely. |
| Blinding of outcome assessment (detection bias) – All outcomes | High risk | Quote: “All the X-rays were scrutinised by a radiologist at the University Hospital in Uppsala. The repeated examinations performed during the postoperative observation years made it easier to diagnose a recurrence”. Comment: insufficient information provided, however it is unlikely. |
| Incomplete outcome data (attrition bias) – All outcomes | Unclear risk | Attrition rates were low and balanced across groups, however reasons were not reported. |
| Selective reporting (reporting bias) | Low risk | All outcomes stated in the methods section were reported. |
| Other bias | High risk | Baseline characteristics not provided. Imbalance in numbers randomised (40 vs 57). |

| Bommelaer 2020 | | |
| --- | --- | --- |
| Bias | Author’s judgement | Support for judgement |
| Random sequence generation (selection bias) | Low | permuted-block randomization (ie, random block sizes) was conducted using a computer-generated random allocation (Stata software version 13, StataCorp, College Station, TX), with a 1:1 ratio allocation. The randomization was performed centrally and stratified by centers. |
| Allocation concealment (selection bias) | Low | The investigated drugs (curcumin or placebo) were delivered within each center according to the central randomization. |
| Blinding of participants and personnel (performance bias) | Low | All participating physicians and patients were blinded from treatment assignment throughout the study. Identical placebo capsules |
| Blinding of outcome assessment (detection bias) | Low | Colonoscopic examinations at M6 were recorded to be scored by independent central readers (G.B. and A.B.) from the coordinating center (Clermont-Ferrand) |
| Incomplete outcome data (attrition bias) | Low | Similar number and reasons of withdrawals in each group. |
| Selective reporting (reporting bias) | Low | All outcomes reported properly. |
| Other bias | High | Differences in gender and perianal lesions at baseline. |

| Brignola 1995 | | |
| --- | --- | --- |
| bias | Authors' judgement | Support for judgement |
| Random sequence generation (selection bias) | Unclear risk | Quote: “Each center received material for at least 4 cases labelled with a patient code number according to a randomisation made in balanced blocks”  Comment: block random sequence generation, but method not described |
| Allocation concealment (selection bias) | Unclear risk | Quote: “Each center received material for at least 4 cases labelled with a patient code number according to a randomisation made in balanced blocks”  Comment: unclear whether drug containers were identical. Insufficient information to make judgement |
| Blinding of participants and personnel (performance bias) – All outcomes | Low risk | Quote: “The treatment binding code was broken in September 1993 when all the assessments were finished; no serious adverse event necessitated breaking of the code beforehand”  Comment: double-blind trial, participants received placebo tablets identical to the study intervention |
| Blinding of outcome assessment (detection bias) – All outcomes | Low risk | Quote: “Endoscopists, unaware of the treatment that the patient had received, recorded on a standardized form a description of endoscopic lesions by type… At the end of the trial, two investigators not previously involved in the patients’ follow-up and unaware of which treatment the patients had received and also of the overall assessments provided by each center, independently evaluated all standardized forms… The treatment blinding was maintained until after assessments were finished.” |
| Incomplete outcome data (attrition bias) – All outcomes | Low risk | Attrition rates were low and balanced across groups with reasons reported. |
| Selective reporting (reporting bias) | Low risk | Trial registration not available, however all outcomes stated in the methods section were reported. |
| Other bias | Low risk | Quote: “Clinical characteristics that were considered in our trial were well balanced between the mesalamine group and the placebo.”  Comment: groups well balanced at baseline. No other apparent biases. |

| Caprilli 1994 | | |
| --- | --- | --- |
| Bias | Authors’ judgement | Support for judgement |
| Random sequence generation (selection bias) | Unclear risk | Quote: “Eligible patients were randomly allocated to receive 2.4 g/day of Eudragit-S coated mesalazine (Asacol, Bracco SPA, Italy) or no treatment at all.” Comment: insufficient information on random sequence generation |
| Allocation concealment (selection bias) | Unclear risk | Insufficient information to make judgement |
| Blinding of participants and personnel (performance bias) – All outcomes | High risk | Quote: “This multicentre study was not blind.” Comment: open-label study design |
| Blinding of outcome assessment (detection bias) – All outcomes | Low risk | Quote: “On the first occasion, the endoscopist was unaware of the treatment; on the second, the tapes were shown with a different sequence and the endoscopist was informed of treatment… The variability sources of the recurrence classification were evaluated… However, the results of the reliability study suggest that lack of blindness in the endoscopists collaborating on the trial was not relevant. In fact, we found that the endoscopists were not in disagreement in the assessment of recurrence nor was the diagnosis of recurrence affected by endoscopists' awareness of the kind of treatment.” Comment: there was some form of blind outcome assessment, and the reliability study comparing blind vs unblind assessment showed that lack of blinding had no effect on outcome assessment |
| Incomplete outcome data (attrition bias) – All outcomes | Low risk | Quote: “The cumulative proportions of symptomatic recurrence and asymptomatic recurrence were estimated by the life-table method on the intention-to-treat principle.” Comment: attrition rate was low and balanced across groups |
| Selective reporting (reporting bias) | Low risk | All outcome data stated in the methods section were reported. |
| Other bias | Low risk | Quote: “The groups were homogenous for age, duration of the disease, site and extent of the lesions, clinical course perforating or non-perforating, previous treatment, indication and type of surgery, and CDAI score at operation. Males more common in MEZ group.” Comment: groups well balanced at baseline. No other apparent sources of bias detected. |

| Chermesh 2007 | | |
| --- | --- | --- |
| Bias | Authors’ judgement | Support for judgement |
| Random sequence generation (selection bias) | Low risk | Quote: “Patients were randomized to active treatment or placebo in a 2:1 ratio.” Comment: insufficient information provided, however authors contacted on 3 August 2018 and indicated that randomisation was done manually at the medical centre. |
| Allocation concealment (selection bias) | High risk | Insufficient information provided, however authors contacted and confirmed that “predefined notes with allocation were prepared, and for each patient a note with the treatment group allocation was drawn”. We do not consider this sufficient to prevent bias. |
| Blinding of participants and personnel (performance bias) – All outcomes | Low risk | The study was placebo-blinded. |
| Blinding of outcome assessment (detection bias) – All outcomes | Unclear risk | The study was referred to as double-blinded, however there is insufficient information to permit judgement. |
| Incomplete outcome data (attrition bias) – All outcomes | Low risk | Half of the randomised participants dropped out of the trial. This early discontinuation of the study was due to an interim analysis that found no benefit of the active treatment. We do not consider this a source of bias. |
| Selective reporting (reporting bias) | High risk | Trial registration was not available. CDAI and Rutgeerts mean score were reported for the control group, but not for the active treatment group; instead they merely reported as “NS” (not significant). |
| Other bias | Low risk | Quote: “No differences were found between the 2 treatment groups regarding gender, age at diagnosis, age at surgery, weight, smoking status, type of disease, length of resected segment, or medical treatment prior to surgery.” Comment: groups balanced at baseline. No other apparent sources of bias detected. |

| D'Haens 2008 | | |
| --- | --- | --- |
| Bias | Authors’ judgement | Support for judgement |
| Random sequence generation (selection bias) | Low risk | Quote: “The random allocation sequence was delivered by a randomization program written in Visual Basic version 6.” Comment: computer-generated randomisation. |
| Allocation concealment (selection bias) | Unclear risk | Quote: “Randomization took place in the pharmacy of the Leuven University Hospitals within 2 weeks after surgery.” Comment: insufficient information to make judgement. |
| Blinding of participants and personnel (performance bias) – All outcomes | Unclear risk | Dummy tablets used; study was single-blinded. It is unclear whether personnel were blinded or not. |
| Blinding of outcome assessment (detection bias) – All outcomes | Low risk | Quote: “At week 12 and 52, an ileocolonoscopy was performed with determination of Rutgeerts’ score for ileal recurrence of CD by an endoscopist who was unaware of treatment assignment.” Comment: probably done. |
| Incomplete outcome data (attrition bias) – All outcomes | Low risk | Quote: “Both intention-to-treat and per-protocol analyses were performed.” Comment: ITT analysis applied, and attrition rates were similarly low across groups. |
| Selective reporting (reporting bias) | Low risk | Trial registration not available; however all outcomes stated in the methods section were adequately reported. |
| Other bias | Low risk | Quote: “The characteristics of the study populations in the AZA and placebo group were comparable.” Comment: groups well balanced at baseline; no other apparent sources of bias detected. |

| D'Haens 2025 | | |
| --- | --- | --- |
| Bias | Author’s judgement | Support for judgement |
| Random sequence generation (selection bias) | Low | Randomisation (1:1) was performed centrally by the project manager with the use of a computer-generated validated variable block model |
| Allocation concealment (selection bias) | Low | The randomisation outcome was communicated to local trial pharmacies via email. Patients were enrolled by the study team at each institution. |
| Blinding of participants and personnel (performance bias) | Low | Patients and all trial personnel, except for the study pharmacist, were unaware of treatment assignment. Masking was achieved as follows: the solution bag was covered with a blinding sleeve, a fake needle prick was applied at the injection site or port of the infusion bag to imitate injected fluid (in the placebo group), and a blinded sticker was applied on the solution bag and the blinding sleeve |
| Blinding of outcome assessment (detection bias) | Low | “All readers assessing endoscopic and histological inflammation were unaware of any clinical information and randomisation.” |
| Incomplete outcome data (attrition bias) | Low | Low and balanced number and reasons of withdraws |
| Selective reporting (reporting bias) | Low | Protocol provided, data reported properly |
| Other bias | Unclear | Most characteristics were balanced at the baseline except for the maximum CDAI values between the two groups differ. |

| De Bruyn 2021 | | |
| --- | --- | --- |
| Bias | Authors’ judgement | Support for judgement |
| Random sequence generation (selection bias) | Unclear risk | Insufficient information to make judgement |
| Allocation concealment (selection bias) | Unclear risk | Insufficient information to make judgement |
| Blinding of participants and personnel (performance bias) – All outcomes | Low risk | Described as a double-blind study. |
| Blinding of outcome assessment (detection bias) – All outcomes | Low risk | Primary outcome assessed blinded centrally read endoscopic recurrence |
| Incomplete outcome data (attrition bias) – All outcomes | Low risk | Balanced attrition and reasons provided. |
| Selective reporting (reporting bias) | Low risk | Outcomes reported appropriately as per trial registration NCT02010762. |
| Other bias | Low risk | No major baseline differences between groups. |

| Duan 2024 | | |
| --- | --- | --- |
| Bias | Author’s judgement | Support for judgement |
| Random sequence generation (selection bias) | Low | fixed block randomization; computer-generated [SAS Institute, version 9·2] |
| Allocation concealment (selection bias) | Low | use of sealed, sequentially numbered, opaque envelopes. This was done by a statistician who did not directly participate in trial recruitment or enrolment. |
| Blinding of participants and personnel (performance bias) | High | investigators, surgical team, and patients were not blinded to the group assignment, the data analyser and the endoscopist remained blinded and were not involved in patient care |
| Blinding of outcome assessment (detection bias) | High | Open label study. investigators, surgical team, and patients were not blinded to the group assignment, the data analyser and the endoscopist remained blinded and were not involved in patient care |
| Incomplete outcome data (attrition bias) | Low | Balanced reason in each group |
| Selective reporting (reporting bias) | Low | Protocol provided, data reported properly |
| Other bias | Unclear | Balanced baseline characteristics but no baseline CDAI |

| Ewe 1999 | | |
| --- | --- | --- |
| Bias | Authors’ judgement | Support for judgement |
| Random sequence generation (selection bias) | Low risk | Quote: “83 patients were randomized according to a computer-generated list”  Comment: computer random number generator |
| Allocation concealment (selection bias) | Unclear risk | Insufficient information to make judgement |
| Blinding of participants and personnel (performance bias) – All outcomes | Low risk | Quote: “This study is a double-blind placebo-controlled clinical trial involving three university-based medical centres […] Placebo medication was indistinguishable from budesonide”  Comment: placebo-controlled |
| Blinding of outcome assessment (detection bias) – All outcomes | Unclear risk | Quote: “All biopsies were evaluated independently by the pathologists at the three study centres and uncertain diagnoses were discussed at a joint meeting”  Comment: the study was reportedly double-blinded, however there is insufficient information to determine whether the pathologists were aware of the interventions to which participants were allocated |
| Incomplete outcome data  (attrition bias)  All outcomes | Unclear risk | Quote: "Calculations were performed based on all patients with Crohn's disease who had been operated on as outlined above and had taken the study  medication for at least 1 day (intention-to-treat)”  Comment: ITT was performed. However, over 20% of participants were withdrawn from the study, and there is insufficient information to determine how  this compares with the event risk. |
| Selective reporting (reporting bias) | Low risk | Trial registration not available, however all outcomes stated in the methods  section were reported |
| Other bias | Low risk | Quote: "Both groups were comparable with regard to their demographic and disease characteristics”  Comment: both groups well balanced at baseline |

| Fukushima 2018 | | |
| --- | --- | --- |
| Bias | Authors' judgement | Support for judgement |
| Random sequence generation (selection bias) | Low risk | Quote: “Eligible and consenting patients were assigned randomly to be treated with or without infliximab (IFX) by Keio University Hospital, Clinical and Translational Research Center, within 4 weeks of resection”  Comment: insufficient information to make judgement. However, authors contacted, response as follows (quote): “In practice, when patients agreed with the study, we sent a fax to the Keio University Hospital, Clinical and Translational Research Center, where randomization was carried out using random number. Then Keio University Hospital, Clinical and Translational Research Center sent back the decision (Infliximab or without infliximab). Random number generated by computer”. Comment: computer-generated random sequence. |
| Allocation concealment (selection bias) | Low risk | Appears to have been centrally allocated based on the information above. |
| Blinding of participants and personnel (performance bias) – All outcomes | High risk | Open-label pilot study. |
| Blinding of outcome assessment (detection bias) – All outcomes | High risk | Highly unlikely, open-label pilot study. |
| Incomplete outcome data (attrition bias) – All outcomes | Low risk | Quote: “Patients who dropped out of follow-up, did not undergo endoscopy at 24 months, or had adverse effects leading to withdrawal from the study were treated as recurrent cases”  Comment: ITT analysis applied, however there was about 25% attrition rate which was considered insufficient to introduce bias. |
| Selective reporting (reporting bias) | Low risk | Trial registration available (UMIN000002604), and all proposed outcomes were reported. |
| Other bias | Low risk | Quote: “There were no statistical differences between the two groups in history of IFX therapy, smoking behavior, surgical indication, site of disease, or type of anastomosis”  Comment: groups balanced at baseline. No other apparent sources of bias detected. |

| Hanauer 2004 | | |
| --- | --- | --- |
| Bias | Authors' judgement | Support for judgement |
| Random sequence generation (selection bias) | Low risk | Quotes: “Patients were randomized by a central computer by permuted blocks of 6 (unknown to investigators) per center to receive mesalamine (Pentasa; Marion Merrill Dow, Kansas City, MO) 3 g daily, 6-MP (Purienthol; Burroughs Wellcome, Research Triangle Park, NC) 50 mg daily, or placebo”  Comment: computer-generated random sequence |
| Allocation concealment (selection bias) | Low risk | Quotes: “Medications were prepared and dispensed by an assigned pharmacist at each site's investigational pharmacy who was not directly involved in the care of the patients”  Comment: treatment controlled by pharmacies at each centre |
| Blinding of participants and personnel (performance bias) – All outcomes | Unclear risk | Quotes: “Medications were prepared and dispensed by an assigned pharmacist at each site’s investigational pharmacy who was not directly involved in the care of the patients” and “An evaluating (treating) physician followed up each patient and was blinded as to the study drug and laboratory results”  Comment: placebo-controlled, double-blind RCT. However, it is unclear whether both study drugs were sufficiently identical to the placebo to blind study participants |
| Blinding of outcome assessment (detection bias) – All outcomes | Low risk | Quotes: “Patient evaluation consisted of assessments of clinical, endoscopic, and radiographic disease activity at each study site by the blinded physician” and “Colonoscopic examinations with endoscopic descriptions and photography of the anastomosis and preanastomotic ileum were performed by the blinded investigators (all gastroenterologists) at months 6, 12, and 24” and “Radiographic interpretations were performed by the blinded inflammatory bowel disease radiologist at each institution”  Comment: assessors blinded to treatment |
| Incomplete outcome data (attrition bias) – All outcomes | Low risk | Quotes: “The clinical recurrence rates were determined using ITT”  Comment: ITT analysis applied, attrition was similar, low, and balanced across groups |
| Selective reporting (reporting bias) | Low risk | Comment: all outcomes stated in the methods section were reported |
| Other bias | Low risk | Quote: “There were no statistical differences in patient age, sex, disease duration, indications for surgical resection, or preoperative disease activity among the 3 groups”  Comment: groups well balanced at baseline. No other apparent sources of bias detected |

| Hellers 1999 | | |
| --- | --- | --- |
| Bias | Authors' judgement | Support for judgement |
| Random sequence generation (selection bias) | Unclear risk | Quote: "Patients were then randomized to treatment with either budesonide CIR, 6 mg/day[…] The randomization code was not broken until each patient’s file was complete and approved for statistical analysis and adverse event evaluation"  Comment: it is unclear how the randomisation codes were generated |
| Allocation concealment (selection bias) | Unclear risk | Insufficient data to make judgement |
| Blinding of participants and personnel (performance bias) – All outcomes | Low risk | Quote: "The randomization code was not broken until each patient’s file was complete and approved for statistical analysis and adverse event evaluation"  Comment: study is placebo controlled, and blinding appeared to have remained unbroken until all outcomes were collected |
| Blinding of outcome assessment (detection bias) – All outcomes | Low risk | Quote: "The randomization code was not broken until each patient’s file was complete and approved for statistical analysis and adverse event evaluation"  Comment: not explicitly stated, however blinding appeared to have remained unbroken until all outcomes were collected |
| Incomplete outcome data (attrition bias) – All outcomes | Low risk | Attrition rates and reasons were similar and balanced across groups. |
| Selective reporting (reporting bias) | Low risk | Trial registration not available, however all outcomes stated in the methods section were reported |
| Other bias | Low risk | Quote: "The two groups were similar in terms of characteristics and disease history"  Comment: baseline characteristics were balanced across groups, and there were no other apparent biases |

| Herfarth 2006 | | |
| --- | --- | --- |
| Bias | Authors’ judgement | Support for judgement |
| Random sequence generation (selection bias) | Low risk | "Patients in the present study were assigned to one of the two treatment groups (5-ASA or azathioprine) at random |
| Allocation concealment (selection bias) | Low risk | "The randomization code was prepared and stored by a statistician from a CRO, who was not involved in the conduct nor in the analysis of the study. The Qualified Person of the Sponsor and the contract manufacturer responsible for the preparation of the double-dummy patients sets received a copy of the randomization list, which was safely stored at both sites, without allowing access by other people. Neither the investigator nor the study team from the clinical operation from the sponsor nor the CRO had access to the random list" |
| Blinding of participants and personnel (performance bias) – All outcomes | Low risk | "This was a double-blind, double-dummy study. Patients randomized to administer 5-ASA had to take 5-ASA VERUM tablets AND azathioprine PLACEBO tablets. Patients randomized to receive azathioprine had to administer azathioprine VERUM tablets AND 5-ASA PLACEBO tablets |
| Blinding of outcome assessment (detection bias) – All outcomes | Low risk | "This was a double-blind, double-dummy study. Patients randomized to administer 5-ASA had to take 5-ASA VERUM tablets AND azathioprine PLACEBO tablets. Patients randomized to receive azathioprine had to administer azathioprine VERUM tablets AND 5-ASA PLACEBO tablets |
| Incomplete outcome data (attrition bias) – All outcomes | High risk | Quote: "The study was stopped prematurely after an interim-analysis due to a high therapy failure rate. 38 patients (AZA 18 pat.; 5-ASA 20 pat.) completed the study and could be evaluated regarding the primary endpoint therapy failure. The other pat. terminated the trial prematurely due to the study stop, but were also evaluated for adverse events (AE) and adverse drug reactions (ADR)" |
| Selective reporting (reporting bias) | Unclear risk | Insufficient information as trial registration was not available and study was published as abstract |
| Other bias | Unclear risk | Insufficient information as study was published as abstract |

| Herfarth 2013 | | |
| --- | --- | --- |
| Bias | Authors' judgement | Support for judgement |
| Random sequence generation (selection bias) | Low risk | Quote: "Patients were randomized in a 1:1 ratio to oral treatment with ciprofloxacin 500 mg or identical appearing placebo twice daily for 6 months. Randomization took place at the trial central pharmacy at the University of North Carolina. Randomization was performed by permuted block randomization with a block size of 4 per site"  Comment: block random sequence generation |
| Allocation concealment (selection bias) | Low risk | Centralised allocation by the pharmacy |
| Blinding of participants and personnel (performance bias) – All outcomes | Low risk | Placebo-controlled, double-blind trial, however no information regarding the blinding of personnel provided |
| Blinding of outcome assessment (detection bias) – All outcomes | Low risk | Quote: "Also photo-documentation of the anastomosis and neoterminal ileum of each patient was reviewed in a blinded fashion by two of the investigators (H.H., K.I.). All scores of this second evaluation were in agreement with the initial evaluation"  Comment: outcome assessors blinded to treatmen |
| Incomplete outcome data (attrition bias) – All outcomes | High risk | Quote: "For the ITT analysis patients without ileocolonoscopy and clinical evaluation at the 6-months visit were considered to have endoscopic and clinical recurrence of CD"  Comment: ITT analysis applied; however, overall attrition rate of over 30%when compared to event risk of 24% raises concerns about bias |
| Selective reporting (reporting bias) | Low risk | Trial registration is available (NCT00609973). All proposed outcomes were reported. |
| Other bias | Low risk | Baseline characteristics balanced. No other apparent sources of bias detected. |

| Hirsch 2023 | | |
| --- | --- | --- |
| Bias | Author’s judgement | Support for judgement |
| Random sequence generation (selection bias) | Low | Patients were randomized using a predetermined randomization log. Based on a predetermined 1:1 ratio, patients were assigned to either therapy in blocks of 10. Within each block, patients were assigned to each treatment arm in a randomized fashion. |
| Allocation concealment (selection bias) | Unclear | No information on allocation. |
| Blinding of participants and personnel (performance bias) | High | Open label |
| Blinding of outcome assessment (detection bias) | Low | Two physicians who were blinded to the patients’ study group performed the endoscopies and graded the anastomotic appearance. |
| Incomplete outcome data (attrition bias) | Unclear | All 35 patients underwent ileocolonoscopy. However, not sure about the withdrawn at week 58.  Patients were excluded for early postoperative complications (n = 1), CD involving proximal small bowel (n = 1) and withdrawal of consent (n = 4). – Is this before or after randomization? |
| Selective reporting (reporting bias) | High | In trial registration, primary outcome was planned to measure at week 52, but actually at week 58. The secondary outcome “clinical remission at 12 months assessed by the CDAI” was not reported. |
| Other bias | Unclear | Differences in Preop therapy and SF36. |

| Lochs 2000 | | |
| --- | --- | --- |
| Bias | Authors' judgement | Support for judgement |
| Random sequence generation (selection bias) | Low risk | Quote: "A computer-generated randomization scheme was provided by the Institut für Medizinische Dokumentation und Statistik at the University of Köln at the beginning of the trial and forwarded to the Department of Galenics at Ferring A/S, Denmark. Randomization was performed in blocks of 10 for each of the participating centers." Comment: computer-generated random sequence. |
| Allocation concealment (selection bias) | Unclear risk | Quote: "In addition, each center retained sealed opaque envelopes containing patient numbers and treatment allocations, which were only allowed to be opened in case of a serious adverse event that necessitated disclosure of the type of treatment." Comment: unclear whether envelops were sequentially numbered. |
| Blinding of participants and personnel (performance bias) – All outcomes | Low risk | Quote: "Placebo tablets of identical appearance and consistency contained additional microcrystalline cellulose to compensate for the mesalamine microgranules… All patients and investigators were blinded regarding treatment allocation." Comment: placebo blinded. |
| Blinding of outcome assessment (detection bias) – All outcomes | Low risk | Quote: "Randomization was performed in blocks of 10 for each of the participating centers. This information was kept confidential at the Department of Quality Assessment at Ferring and the statistical center in Cologne and was only available to the Department of Galenics […] An Endpoint Committee consisting of 2 physicians and 1 surgeon, not participating in the trial, made a final decision about questionable cases of protocol violations and relapses." Comment: probably done. |
| Incomplete outcome data (attrition bias) – All outcomes | Low risk | Quotes: "Outcome measures were analysed in all randomized patients who had taken at least 1 dose of study medication (intention-to-treat population)." Comment: attrition rates and reasons were balanced across groups. |
| Selective reporting (reporting bias) | Low risk | Trial registration not available, however all outcomes stated in the methods section were reported. |
| Other bias | Low risk | Quote: "No significant differences were detected between the 2 treatment groups for any of the parameters investigated." Comment: groups well balanced at baseline. No additional sources of bias detected. |

| Lopez Sanroman 2017 | | |
| --- | --- | --- |
| Bias | Authors' judgement | Support for judgement |
| Random sequence generation (selection bias) | Low risk | Quote: “Central randomisation was based on a pregenerated block randomisation list stratified by centre.” and “Patients were assigned [1:1] to…”  Comment: central randomisation |
| Allocation concealment (selection bias) | Unclear risk | Quote: “Allocation was concealed by means of a computer-generated randomisation schedule without stratification or block allocation”  Comment: insufficient description |
| Blinding of participants and personnel (performance bias) – All outcomes | High risk | Quote: “Neither patients nor investigators were blinded to the administered treatment”  Comment: no blinding of personnel and participants performed |
| Blinding of outcome assessment (detection bias) – All outcomes | Low risk | Quote: “A video recording of the last 15 cm of the neo-terminal ileum was evaluated by an endoscopist blinded to treatment allocation and experienced in application of the Rutgeerts score [VP]” and “…MRE, which was evaluated centrally by an experienced blinded reader [JR]”  Comment: outcome assessors were blinded to treatment |
| Incomplete outcome data (attrition bias) – All outcomes | Low risk | Quote: “We defined the following populations: 1) the intention-to-treat [ITT] population, which included all consenting patients who were randomised and received at least one dose of the study medications”  Comment: ITT analysis applied, reasons for withdrawal reported, and attrition rates were balanced across groups |
| Selective reporting (reporting bias) | High risk | Trial registration was available (NCT01564823), and all prespecified outcomes were reported in the study except for health-related quality of life, which was only reported as a P value in an abstract. |
| Other bias | Low risk | Quote: “The groups were similar regarding baseline characteristics, including smoking status, previous resections, CD phenotype, previous perianal disease, and previous drug exposure”  Comment: groups well balanced at baseline. No other apparent sources of bias detected. |

| Marteau 2006 | | |
| --- | --- | --- |
| Bias | Authors' judgement | Support for judgement |
| Random sequence generation (selection bias) | Low risk | Quote: “… randomisation was performed by this centre within each stratum per centre, using permutation tables of size 2 or 4, according to expected enrolment within each centre, each centre being blinded to the size of its blocks”  Comment: block randomisation |
| Allocation concealment (selection bias) | Low risk | Quote: “Treatment number was the first free number with the corresponding treatment in a randomised list with treatment numbers and their corresponding treatment prepared by the biostatistics centre before trial initiation” and “The same information and allocated treatment were sent to the service in charge of drug delivery, allowing the service to check that treatment was in agreement with the predefined list. Treatment was sent by this service to the pharmacy of the centre with protocol identification and the patient’s identification”  Comment: central allocation |
| Blinding of participants and personnel (performance bias) – All outcomes | Low risk | Placebo-blinded study |
| Blinding of outcome assessment (detection bias) – All outcomes | Low risk | The study was double-blinded and “unblinding, if necessary, was made by a request to the biostatistics centre with a specific form”.  Comment: probably done |
| Incomplete outcome data (attrition bias) – All outcomes | Low risk | Quote: “The primary efficacy analysis was based on the ITT population, which included all patients in whom the primary endpoint was assessable”  Comment: attrition rates and reasons were balanced across groups |
| Selective reporting (reporting bias) | Low risk | Comment: all outcomes stated in the methods section were reported. |
| Other bias | Low risk | Quote: “The two treatment groups were well matched, except for a higher proportion of patients who underwent ileal and colon resection in the placebo group, and a higher median CRP level in the LAI group”  Comment: groups well balanced at baseline; no other apparent sources of bias detected. |

| McLeod 1995 | | |
| --- | --- | --- |
| Bias | Authors' judgement | Support for judgement |
| Random sequence generation (selection bias) | Low risk | Quote: "The randomization scheme was computer generated by the Clinical Research Support Unit, University of Toronto, and maintained by the pharmacies at the Toronto Hospital, General Division, and St. Mary's Hospital, Rochester." Comment: computer-generated random sequence. |
| Allocation concealment (selection bias) | Low risk | Quote: "All investigators and patients were blinded with respect to treatment allocation." Comment: no further details provided, however the authors confirmed on 27 November 2009 that a central allocation was done by pharmacy. |
| Blinding of participants and personnel (performance bias) – All outcomes | Low risk | Quote: "Subjects in the control group took six identical-looking placebo tablets twice daily." Comment: participants and investigators were blinded to treatment. |
| Blinding of outcome assessment (detection bias) – All outcomes | Low risk | Quote: "All patient records were reviewed by an adjudication committee of five investigators (R.S.M., B.G.W., A.H.S., P.W.C., and K.C.) blinded to patient treatment allocation." "The charts of patients who were noncompliant were reviewed by two blinded gastroenterologists (A.H.S. and P.W.C.), who determined whether noncompliance was secondary to adverse effects potentially related to the medication." Comment: blinding of assessors performed. |
| Incomplete outcome data (attrition bias) – All outcomes | Low risk | Attrition rates were low, and reasons for withdrawal were balanced across groups. |
| Selective reporting (reporting bias) | Low risk | Trial registration not available, however all outcomes stated in the methods section were reported. |
| Other bias | Low risk | Quote: "The characteristics of the two groups, which are listed in Table 1, were similar." Comment: groups balanced at baseline. No other apparent sources of bias detected. |

| Mowat 2016 | | |
| --- | --- | --- |
| Bias | Authors' judgement | Support for judgement |
| Random sequence generation (selection bias) | Low risk | Quote: "Patients were randomly assigned (1:1) to mercaptopurine or identical matched placebo using a computer-generated web-based randomisation system managed by the Edinburgh Clinical Trials Unit (University of Edinburgh, Edinburgh, UK)".  Comment: computer-generated web-based random sequence. |
| Allocation concealment (selection bias) | Low risk | Quote: "Patients’ details were entered into the randomisation system before random allocation and were concealed at randomisation".  Comment: web-based central allocation. |
| Blinding of participants and personnel (performance bias) – All outcomes | Low risk | Quote: "Patients and their carers and physicians were masked to the treatment allocation".  Comment: the study is placebo-controlled. |
| Blinding of outcome assessment (detection bias) – All outcomes | Low risk | Quote: "Blood monitoring results were reviewed by an independent central clinician masked to treatment allocation and to mean corpuscular volume results. To protect masking, investigators were informed that sham dose reductions were planned for patients on placebo. However, on the advice of the data monitoring committee, sham dose reductions did not occur; the investigators were not informed of this".  Comment: outcome assessors were blinded to treatment. |
| Incomplete outcome data (attrition bias) – All outcomes | Low risk | Quote: "Analyses were by intention to treat". Comment: attrition rate of 23% when compared with the event risk (30%) was not considered sufficient to lead to bias. |
| Selective reporting (reporting bias) | Low risk | Trial registration available (ISRCTN89489788), and all outcomes stated in the methods section were reported. |
| Other bias | Low risk | Quote: "Baseline characteristics were similar between study groups".  Comment: groups well balanced at baseline; no other apparent sources of bias detected. |

| Orlando 2020 | | |
| --- | --- | --- |
| Bias | Authors' judgement | Support for judgement |
| Random sequence generation (selection bias) | Low risk | Central randomization was performed via computer-generated randomization lists with medication distributed to each center accordingly. |
| Allocation concealment (selection bias) | Low risk | Central randomization was performed via computer-generated randomization lists with medication distributed to each center accordingly. |
| Blinding of participants and personnel (performance bias) – All outcomes | Low risk | Comment: trial was reportedly double-blinded and placebo controlled; probably done. |
| Blinding of outcome assessment (detection bias) – All outcomes | Unclear risk | Insufficient information to make judgement. |
| Incomplete outcome data (attrition bias) – All outcomes | Low risk | No attrition. |
| Selective reporting (reporting bias) | Low risk | Outcomes appropriately reported per trial registration EUDRACT 2006-001315-30. |
| Other bias | Low risk | groups balanced at baseline, except for one characteristic. No other apparent sources of bias detected. |

| Prantera 2002 | | |
| --- | --- | --- |
| Bias | Authors' judgement | Support for judgement |
| Random sequence generation (selection bias) | Low risk | Quote: "Using computerised randomisation in blocks of two, patients were allocated to receive bags of either Dicoflor 60 or placebo". Comment: probably done. |
| Allocation concealment (selection bias) | Unclear risk | Insufficient information provided. |
| Blinding of participants and personnel (performance bias) – All outcomes | Low risk | Quote: "The placebo consisted of bags of identical appearance to the probiotic […] The taste and smell of the active substance and placebo were the same". Comment: trial was reportedly double-blinded and placebo controlled; probably done. |
| Blinding of outcome assessment (detection bias) – All outcomes | Unclear risk | Insufficient information to make judgement. |
| Incomplete outcome data (attrition bias) – All outcomes | Low risk | Attrition balanced, all participants accounted for, withdrawals and reasons reported. |
| Selective reporting (reporting bias) | Low risk | All outcomes stated in the methods section were reported. |
| Other bias | Low risk | Quote: "Demographic and disease characteristics did not differ significantly between the two groups but a higher percentage of patients treated with LGG were smokers". Comment: groups balanced at baseline, except for one characteristic. No other apparent sources of bias detected. |

| Regueiro 2009 | | |
| --- | --- | --- |
| Bias | Authors' judgement | Support for judgement |
| Random sequence generation (selection bias) | Low risk | Quote: "All 24 patients underwent ileocolonic resection with primary anastomosis and were then randomized to placebo or infliximab". Comment: insufficient information to make judgement. However, email received from authors on 2 August 2018 stating: "The allocation was done by the central (university) pharmacy. The randomization was blocked". Comment: block randomisation performed by a pharmacy. |
| Allocation concealment (selection bias) | Low risk | Quote: "The allocation was done by the central (university) pharmacy. The randomization was blocked". Comment: central allocation. Email received from authors on 2 August 2018 indicated that "Pharmacy maintained the blind". |
| Blinding of participants and personnel (performance bias) – All outcomes | Low risk | Insufficient information to make judgement. However email received from authors on 2 August 2018 stating: "The study drug (infliximab or placebo) was delivered from the pharmacist to the research nurse and was blinded. The only unblinded person was the central pharmacist who did the block allocation for randomization. The study drug was unidentified and all study personnel were not aware of the treatment allocation". Comment: participants and personnel blinded to treatment. |
| Blinding of outcome assessment (detection bias) – All outcomes | Low risk | Quote: "A blinded investigator (L.B.) reviewed each patient's video recorded procedure and provided a separate endoscopic score. The colonoscopic video recordings were placed on compact discs that were devoid of patient identifiers (i.e., blinded). At the conclusion of the study, the principal investigator (M.R.) rescored each patient by re-reviewing the video recordings in a random and blinded fashion." "By using standard biopsy forceps, 6–8 biopsy specimens were taken from the neoterminal ileum and assessed blindly by a gastrointestinal pathologist (A.R.S.)". Comment: all outcome assessors blinded to treatment. |
| Incomplete outcome data (attrition bias) – All outcomes | Low risk | Comment: low attrition rates, which were balanced across groups (2/11 vs 1/13). |
| Selective reporting (reporting bias) | Low risk | Trial registration was available (NCT00688636), and all proposed outcomes were reported. |
| Other bias | High risk | Quote: "In the infliximab group, there were significantly more active smokers (45.5% vs 7.7%; P = .06), and a trend for less concomitant immunomodulators use (36.4 vs 53.8%; P = .44) or mesalamine use (9.1% vs 30.8%; P = .33). In addition, the median baseline ESR was significantly higher in the infliximab group (40 vs 11; P = .04), as was the median CRP concentrations (0.5 vs 0.1; P = .05)". Comment: several significant differences between groups at baseline. There are indications that allocation may not have been truly random. |

| Regueiro 2016 | | |
| --- | --- | --- |
| Bias | Authors' judgement | Support for judgement |
| Random sequence generation (selection bias) | Unclear risk | Quote: "Patients were randomized equally to receive infliximab (Remicade; Janssen Biotech, Inc., Horsham Township, PA) 5 mg/kg or placebo every 8 weeks. Randomization was stratified by the number of risk factors for recurrence (1 or >1) and current use of an immunosuppressive (yes/no)". Comment: stratified randomisation. |
| Allocation concealment (selection bias) | Unclear risk | Insufficient information on allocation concealment. |
| Blinding of participants and personnel (performance bias) – All outcomes | Low risk | Quote: "Placebo and infliximab infusions were administered in a blinded manner". Comment: double-blind, placebo-controlled study. |
| Blinding of outcome assessment (detection bias) – All outcomes | Unclear risk | Insufficient information to make judgement on blinding of personnel. |
| Incomplete outcome data (attrition bias) – All outcomes | Unclear risk | Quote: "All randomized patients were included in efficacy analyses according to assigned treatment, regardless of actual treatment received". Comment: however, over 25% of randomized participants withdrew for reasons other than relapse or adverse event; given that the event risk is 21%, it is unclear whether this is sufficient to cause bias. |
| Selective reporting (reporting bias) | Low risk | Trial registration was available (NCT01190839), and all proposed outcomes were reported. |
| Other bias | Low risk | Quote: "Demographics, qualifying characteristics, and risk factors of the 297 randomized patients were similar between treatment groups". Comment: groups were reportedly well balanced at baseline; no other apparent sources of bias detected. |

| Reinisch 2010 | | |
| --- | --- | --- |
| Bias | Authors' judgement | Support for judgement |
| Random sequence generation (selection bias) | Low risk | Quote: "…a central randomisation was performed via five computer-generated randomisation lists (using the program 'Rancode +' (version 3.6) of IDV, Gauting, Germany), which were generated for the five body weight classes (40–50 kg, 51–60 kg, 61–75 kg, 76–100 kg and 101–128 kg), each in blocks of four, with medication distributed to each centre according to this list". Comment: centralised randomisation in blocks of 4. |
| Allocation concealment (selection bias) | Low risk | Central randomisation. |
| Blinding of participants and personnel (performance bias) – All outcomes | Low risk | Quote: "To maintain investigator and patient blinding, patients randomised to azathioprine received verum azathioprine tablets and placebo mesalazine tablets; those randomised to mesalazine received verum mesalazine tablets and placebo azathioprine tablets". Comment: a double-blind, double-dummy RCT. |
| Blinding of outcome assessment (detection bias) – All outcomes | Unclear risk | Insufficient information to make judgement. |
| Incomplete outcome data (attrition bias) – All outcomes | Low risk | Quote: "The intention-to-treat (ITT) population was defined as all randomised patients who received 1 dose of study medication". Comment: the ITT population was defined as all randomised participants who had received 1 dose of study medication. |
| Selective reporting (reporting bias) | Low risk | Trial registration available (NCT00946946), and all prespecified outcomes were reported. |
| Other bias | Low risk | Quote: "Baseline characteristics were similar between treatment groups apart from a lower mean CDAI value in the azathioprine cohort (70 vs 102 in the mesalazine arm) and a higher proportion of azathioprine patients with a penetrating disease behaviour (66% vs 43%)". Comment: some differences at baseline; study supported by Falk Pharma but conflict of interest declared. No other apparent sources of bias detected. |

| Rutgeerts 2005 | | |
| --- | --- | --- |
| Bias | Authors' judgement | Support for judgement |
| Random sequence generation (selection bias) | Unclear risk | Quote: "This randomized double-blind placebo-controlled trial was conducted at the inflammatory bowel disease centers of the University Hospital and 1 large teaching hospital". Comment: insufficient information to make judgement. |
| Allocation concealment (selection bias) | Unclear risk | Insufficient information to make judgement. |
| Blinding of participants and personnel (performance bias) – All outcomes | Low risk | Quote: "The patients received either ornidazole (Tiberal; Roche, Basel, Switzerland) 500 mg twice daily or an identical placebo daily for 54 weeks". Comment: study is placebo controlled and reported as being double-blind. |
| Blinding of outcome assessment (detection bias) – All outcomes | Low risk | Quote: "Biopsy samples of the neoterminal ileum were taken and assessed blindly by 2 pathologists (G.D.H. and K.G.)". Comment: samples taken and assessed by assessors blinded to treatment. |
| Incomplete outcome data (attrition bias) – All outcomes | Low risk | Quote: "Intention-to-treat analysis was performed that included all patients who started the medication". Comment: low attrition rates, which were balanced across groups. |
| Selective reporting (reporting bias) | Low risk | Trial registration not available, however all outcomes stated in the methods section were reported. |
| Other bias | High risk | Quote: "There was a significantly longer duration of disease in the ornidazole group than in the placebo group". Comment: one observed imbalance at baseline, no other apparent sources of bias detected. |

| Savarino 2013 | | |
| --- | --- | --- |
| Bias | Authors' judgement | Support for judgement |
| Random sequence generation (selection bias) | Low risk | Quote: "Eligible and consenting patients were assigned randomly using a computer-generated sequence (www.randomizer.org) to a regimen of…" Comment: computer-generated random sequence. |
| Allocation concealment (selection bias) | Low risk | Quote: "Patient allocation was concealed and performed by an independent nurse not involved with the trial" Comment: probably done. |
| Blinding of participants and personnel (performance bias) – All outcomes | High risk | Study is open-label design. |
| Blinding of outcome assessment (detection bias) – All outcomes | Unclear risk | Quote: "A blinded investigator (P.D.) reviewed each patient’s video-recorded procedure and provided a separate endoscopic score" and "At the conclusion of the study, the principal investigator (E.S.) rescored each patient by re-reviewing the video recordings in a random and blinded manner" Comment: assessors were blinded for endoscopic assessments only; however, no information on clinical assessment of relapse. |
| Incomplete outcome data (attrition bias) – All outcomes | Low risk | Quote: "Statistical analysis was conducted according to the intention-to-treat principle." Comment: the trial had a low attrition rate. Withdrawals and reasons for withdrawals were balanced across groups (1/16 vs 2/17 vs 2/18). |
| Selective reporting (reporting bias) | Low risk | Trial registration not available; however, all outcomes stated in the methods section were reported. |
| Other bias | Low risk | Quote: "Characteristics were similar for sex, age, smoking, duration of CD, disease behavior, disease location, prior medication exposure, including IFX, and prior surgical resection." Comment: groups well balanced at baseline; no other apparent sources of bias detected. |

| Sutherland 1997 | | |
| --- | --- | --- |
| Bias | Authors' judgement | Support for judgement |
| Random sequence generation (selection bias) | Low risk | Quote: "Randomisation was performed according to a computer generated randomisation scheme by the study sponsor." Comment: computer-generated randomisation scheme. |
| Allocation concealment (selection bias) | Low risk | Quote: "For each patient, the identity of the study medication was concealed in an individual sealed envelope sent with the drug supplies" and "Medication was packaged by the sponsor and dispensed to each centre on coded identical-appearing boxes." Comment: sequentially numbered, identically appearing drug packages. |
| Blinding of participants and personnel (performance bias) – All outcomes | Low risk | Quote: "Medication was packaged by the sponsor and dispensed to each centre on coded identical-appearing boxes." Comment: double-blinded, placebo-controlled trial; probably done. |
| Blinding of outcome assessment (detection bias) – All outcomes | Unclear risk | Insufficient information to make judgement. |
| Incomplete outcome data (attrition bias) – All outcomes | Unclear risk | Attrition rates were not reported specifically for the subpopulation of interest in our review (surgical group). |
| Selective reporting (reporting bias) | Low risk | Although adverse event data were not available for the subpopulation of interest in our review (surgical group), all expected outcomes appear to have been reported for the entire population. |
| Other bias | Low risk | Quote: "The demographic characteristics and disease milestones for participants are shown in Table 2. There were no significant differences between the MES- and placebo-treated groups." Comment: supported by a grant by Marion Merrell Dow; author confirmed company had no part in the design, analysis, or write-up of the study. |

| Tursi 2014 | | |
| --- | --- | --- |
| Bias | Authors' judgement | Support for judgement |
| Random sequence generation (selection bias) | Unclear risk | Quote: "Patients were randomized with a simple unblinded 1:1 allocation ratio..." Comment: insufficient information to make judgement. |
| Allocation concealment (selection bias) | Unclear risk | Insufficient information to make judgement. |
| Blinding of participants and personnel (performance bias) – All outcomes | High risk | This is an open-label pilot study, and blinding was not performed. |
| Blinding of outcome assessment (detection bias) – All outcomes | High risk | Quote: "Five unblinded endoscopists (AT, CZ, GP, RF, and GB) did all the examinations and calculated scores. Two further unblinded endoscopists (WE and MP) separately reviewed videos and in case of discordance a consensus agreement was reached among the two operators." Comment: blinding of outcome assessors was not performed. |
| Incomplete outcome data (attrition bias) – All outcomes | Low risk | No missing data; all participants completed the trial. |
| Selective reporting (reporting bias) | Low risk | All outcomes stated in the methods section were reported. |
| Other bias | Low risk | Quote: "There were no differences between baseline characteristics of the two groups: age, duration of disease, active smokers, previous surgery, disease behavior and location, perianal disease at diagnosis, extra intestinal manifestations." Comment: groups well balanced at baseline, and no other apparent sources of bias detected. |

| Wenckert 1978 | | |
| --- | --- | --- |
| Bias | Authors' judgement | Support for judgement |
| Random sequence generation (selection bias) | Low risk | Quote: “The experimental design was double blind multicentre trial with block-randomisation, and no cross-over.” Comment: insufficient data to make judgement. However, author contacted and confirmed that block randomisation described was carried out in accordance with established acceptable randomisation methodology. |
| Allocation concealment (selection bias) | Unclear risk | Comment: insufficient data to make judgement. The author was contacted, but was unable to provide further details. |
| Blinding of participants and personnel (performance bias) – All outcomes | Unclear risk | Comment: insufficient data to make judgement. |
| Blinding of outcome assessment (detection bias) – All outcomes | Unclear risk | Comment: insufficient data to make judgement. |
| Incomplete outcome data (attrition bias) – All outcomes | Low risk | Data reported for those missing; balanced between study groups; reasons for withdrawal unlikely to be related to true outcome. |
| Selective reporting (reporting bias) | Unclear risk | The study includes results for adverse events, but these are not reported clearly enough to permit analysis and a resulting judgement as to risk of bias. |
| Other bias | Unclear risk | Insufficient information to ascertain whether baseline characteristics were balanced. |

| Yoshida 2012 | | |
| --- | --- | --- |
| Bias | Authors' judgement | Support for judgement |
| Random sequence generation (selection bias) | Low risk | Quote: "…randomization was done blindly according to a computer-generated scheme with blocks of two (each two patients were randomly assigned to IFX or to control). This was to minimize the risk of unbalanced group size"  Comment: computer-generated 1:1 randomisation |
| Allocation concealment (selection bias) | Low risk | Quote: "Randomization was done by a statistician at an independent institute"  Comment: probably done |
| Blinding of participants and personnel (performance bias) All outcomes | High risk | Open-label pilot study |
| Blinding of outcome assessment (detection bias) All outcomes | Unclear risk | Quote: "Endoscopic evaluations were performed using a videoscope (CF260AI; Olympus Optics, Tokyo, Japan) by endoscopists who were blinded. Video recording procedures were independently scored by different endoscopists"  Comment: blinding of outcome assessors performed. Insufficient information about clinical assessments |
| Incomplete outcome data (attrition bias) All outcomes | Low risk | All participants accounted for; only 1 withdrawal during study, which was due to adverse events |
| Selective reporting (reporting bias) | Low risk | Trial registration available (UMIN000004427). All outcomes stated in the methods section were reported |
| Other bias | Low risk | Quote: "There was no significant difference between the two groups with respect to entry demography, including smoking behavior"  Comment: groups well balanced at baseline. No other apparent sources of bias detected |

# **eAppendix 2**. Search strategies

**Database: EBM Reviews - Cochrane Central Register of Controlled Trials <December 2024>, Embase <1974 to 2025 February 12>, Ovid MEDLINE(R) ALL <1946 to February 12, 2025>**
**Search Strategy:**
**1**  exp Crohn Disease/
**2**  Crohn*.ti,ab,kw,kf.
**3**  ((inflammatory bowel disease* or IBD) not ulcerative colitis).ti,ab.
**4**  or/1-3 [CD]
**5**  (postoperat* or postsurgical*).ti,ab,kw,kf.
**6**  ((post or after or following or underwent or history or previous* or undergoing or peri-) adj5 (surger* or surgical or operat* or resect* or stricturoplast* or ileostom* or colectom*)).ti,ab,kw,kf.
**7**  (surgically induced adj5 (remission or Quiescence)).ti,ab,kw,kf.
**8**  Postoperative Period/
**9**  or/5-8 [surgically induced remission]
**10**  4 and 9 [CD and post surgery]
**11**  10 use cctr [Cochrane CENTRAL]
**12**  exp randomized controlled trial/ or controlled clinical trial.pt. or clinical trials as topic.sh. or (random* or placebo).ab. or trial.ti. (5976530)
**13**  exp animals/ not humans.sh.
**14**  12 not 13 [Cochrane Highly Sensitive Search Strategy for identifying randomized trials in MEDLINE: sensitivity- and precision-maximizing version (2023 revision); Ovid format]
**15**  10 and 14 use medall [MEDLINE ovid]
**16**  random:.tw. or placebo:.mp. or double-blind:.tw. [Wong 2006 Hedge Therapy filter for EMBASE best balance of sensitivity and specificity, ovid format https://hiruweb.mcmaster.ca/hkr/hedges/embase/]
**17**  10 and 16 use oemezd [Embase ovid]
**18**  11 or 15 or 17
**19**  remove duplicates from 18

# **eAppendix 3**. References to studies included in this review

1. Ardizzone S, Maconi G, Sampietro GM, et al. Azathioprine and mesalamine for prevention of relapse after conservative surgery for Crohn's disease. Gastroenterology 2004;127:730-40.

2. Armuzzi A, Felice C, Papa A, et al. Prevention of postoperative recurrence with azathioprine or infliximab in patients with Crohn's disease: an open-label pilot study. J Crohns Colitis 2013;7:e623-9.

3. Bergman L, Krause U. Postoperative treatment with corticosteroids and salazosulphapyridine (Salazopyrin) after radical resection for Crohn's disease. Scand J Gastroenterol 1976;11:651-6.

4. Bommelaer G, Laharie D, Nancey S, et al. Oral Curcumin No More Effective Than Placebo in Preventing Recurrence of Crohn's Disease After Surgery in a Randomized Controlled Trial. Clinical Gastroenterology and Hepatology 2020;18:1553-1560.e1.

5. Brignola C, Cottone M, Pera A, et al. Mesalamine in the prevention of endoscopic recurrence after intestinal resection for Crohn's disease. Italian Cooperative Study Group. Gastroenterology 1995;108:345-9.

6. Caprilli R, Andreoli A, Capurso L, et al. Oral mesalazine (5-aminosalicylic acid; Asacol) for the prevention of post-operative recurrence of Crohn's disease. Gruppo Italiano per lo Studio del Colon e del Retto (GISC). Aliment Pharmacol Ther 1994;8:35-43.

7. Chermesh I, Tamir A, Reshef R, et al. Failure of Synbiotic 2000 to prevent postoperative recurrence of Crohn's disease. Dig Dis Sci 2007;52:385-9.

8. de Bruyn JR, Bossuyt P, Ferrante M, et al. High-Dose Vitamin D Does Not Prevent Postoperative Recurrence of Crohn's Disease in a Randomized Placebo-Controlled Trial. Clin Gastroenterol Hepatol 2021;19:1573-1582.e5.

9. D'Haens GR, Vermeire S, Van Assche G, et al. Therapy of metronidazole with azathioprine to prevent postoperative recurrence of Crohn's disease: a controlled randomized trial. Gastroenterology 2008;135:1123-9.

10. D'Haens G, Taxonera C, Lopez-Sanroman A, et al. Vedolizumab to prevent postoperative recurrence of Crohn's disease (REPREVIO): a multicentre, double-blind, randomised, placebo-controlled trial. Lancet Gastroenterol Hepatol 2025;10:26-33.

11. Duan M, Lu M, Diao Y, et al. Azathioprine Plus Exclusive Enteral Nutrition Versus Azathioprine Monotherapy for the Prevention of Postoperative Recurrence in Patients with Crohn's Disease: An Open-Label, Single-Centre, Randomized Controlled Trial. J Crohns Colitis 2024;18:1113-1121.

12. Ewe K, Bottger T, Buhr HJ, et al. Low-dose budesonide treatment for prevention of postoperative recurrence of Crohn's disease: a multicentre randomized placebo-controlled trial. German Budesonide Study Group. Eur J Gastroenterol Hepatol 1999;11:277-82.

13. Fukushima K, Sugita A, Futami K, et al. Postoperative therapy with infliximab for Crohn's disease: a 2-year prospective randomized multicenter study in Japan. Surg Today 2018;48:584-590.

14. Hanauer SB, Korelitz BI, Rutgeerts P, et al. Postoperative maintenance of Crohn's disease remission with 6-mercaptopurine, mesalamine, or placebo: a 2-year trial. Gastroenterology 2004;127:723-9.

15. Hellers G, Cortot A, Jewell D, et al. Oral budesonide for prevention of postsurgical recurrence in Crohn's disease. The IOIBD Budesonide Study Group. Gastroenterology 1999;116:294-300.

16. Herfarth H, Tjaden C, Lukas M, et al. Adverse events in clinical trials with azathioprine and mesalamine for prevention of postoperative recurrence of Crohn's disease. Gut 2006;55:1525-6.

17. Herfarth HH, Katz JA, Hanauer SB, et al. Ciprofloxacin for the prevention of postoperative recurrence in patients with Crohn's disease: a randomized, double-blind, placebo-controlled pilot study. Inflamm Bowel Dis 2013;19:1073-9.

18. Hirsch A, Scapa E, Fliss-Isakov N, et al. Early Initiation of Adalimumab Significantly Diminishes Postoperative Crohn’s Disease Endoscopic Recurrence and Is Superior to 6-Mercaptopurine Therapy: An Open-Label, Randomized Controlled Study. Journal of Clinical Medicine 2023;12:7600.

19. Lochs H, Mayer M, Fleig WE, et al. Prophylaxis of postoperative relapse in Crohn's disease with mesalamine: European Cooperative Crohn's Disease Study VI. Gastroenterology 2000;118:264-73.

20. Lopez-Sanroman A, Vera-Mendoza I, Domenech E, et al. Adalimumab vs Azathioprine in the Prevention of Postoperative Crohn's Disease Recurrence. A GETECCU Randomised Trial. J Crohns Colitis 2017;11:1293-1301.

21. Marteau P, Lemann M, Seksik P, et al. Ineffectiveness of Lactobacillus johnsonii LA1 for prophylaxis of postoperative recurrence in Crohn's disease: a randomised, double blind, placebo controlled GETAID trial. Gut 2006;55:842-7.

22. McLeod RS, Wolff BG, Steinhart AH, et al. Prophylactic mesalamine treatment decreases postoperative recurrence of Crohn's disease. Gastroenterology 1995;109:404-13.

23. Mowat C, Arnott I, Cahill A, et al. Mercaptopurine versus placebo to prevent recurrence of Crohn's disease after surgical resection (TOPPIC): a multicentre, double-blind, randomised controlled trial. Lancet Gastroenterol Hepatol 2016;1:273-282.

24. Orlando A, Mocciaro F, Ventimiglia M, et al. Azathioprine for prevention of clinical recurrence in Crohn's disease patients with severe endoscopic recurrence: an IG-IBD randomized double-blind trial. Eur Rev Med Pharmacol Sci 2020;24:11356-11364.

25. Prantera C, Scribano ML, Falasco G, et al. Ineffectiveness of probiotics in preventing recurrence after curative resection for Crohn's disease: a randomised controlled trial with <em>Lactobacillus</em> GG. Gut 2002;51:405-409.

26. Regueiro M, Schraut W, Baidoo L, et al. Infliximab prevents Crohn's disease recurrence after ileal resection. Gastroenterology 2009;136:441-50.e1; quiz 716.

27. Regueiro M, Feagan BG, Zou B, et al. Infliximab Reduces Endoscopic, but Not Clinical, Recurrence of Crohn's Disease After Ileocolonic Resection. Gastroenterology 2016;150:1568-1578.

28. Reinisch W, Angelberger S, Petritsch W, et al. Azathioprine versus mesalazine for prevention of postoperative clinical recurrence in patients with Crohn's disease with endoscopic recurrence: efficacy and safety results of a randomised, double-blind, double-dummy, multicentre trial. Gut 2010;59:752-9.

29. Rutgeerts P, Van Assche G, Vermeire S, et al. Ornidazole for prophylaxis of postoperative Crohn's disease recurrence: a randomized, double-blind, placebo-controlled trial. Gastroenterology 2005;128:856-61.

30. Savarino E, Bodini G, Dulbecco P, et al. Adalimumab is more effective than azathioprine and mesalamine at preventing postoperative recurrence of Crohn's disease: a randomized controlled trial. Am J Gastroenterol 2013;108:1731-42.

31. Sutherland LR, Martin F, Bailey RJ, et al. A randomized, placebo-controlled, double-blind trial of mesalamine in the maintenance of remission of Crohn's disease. The Canadian Mesalamine for Remission of Crohn's Disease Study Group. Gastroenterology 1997;112:1069-77.

32. Tursi A, Elisei W, Picchio M, et al. Comparison of the effectiveness of infliximab and adalimumab in preventing postoperative recurrence in patients with Crohn's disease: an open-label, pilot study. Tech Coloproctol 2014;18:1041-6.

33. Wenckert A, Kristensen M, Eklund AE, et al. The long-term prophylactic effect of salazosulphapyridine (Salazopyrin) in primarily resected patients with Crohn's disease. A controlled double-blind trial. Scand J Gastroenterol 1978;13:161-7.

34. Yoshida K, Fukunaga K, Ikeuchi H, et al. Scheduled infliximab monotherapy to prevent recurrence of Crohn's disease following ileocolic or ileal resection: a 3-year prospective randomized open trial. Inflamm Bowel Dis 2012;18:1617-23.

# **eAppendix 4**. References to studies excluded from this review

35. Angelberger S, Schaeffeler E, Teml A, et al. Mucosal improvement in patients with moderate to severe postoperative endoscopic recurrence of Crohn's disease and azathioprine metabolite levels. Inflamm Bowel Dis 2013;19:590-8.

36. Buisson A, Nancey S, Manlay L, et al. Ustekinumab is more effective than azathioprine to prevent endoscopic postoperative recurrence in Crohn's disease. United European Gastroenterol J 2021;9:552-560.

37. De Cruz P, Kamm M, Hamilton AL, et al. P342 Adalimumab prevents post-operative Crohn's disease recurrence and is superior to thiopurines: Early results from the prospective POCER study. Journal of Crohn's and Colitis 2012;6:S146-S146.

38. De Cruz P, Kamm M, Hamilton A, et al. Strategic timing of anti-TNF therapy in postoperative Crohn's disease: Comparison of routine use immediately postoperatively with selective use after demonstrated recurrence at 6 month endoscopy. Results from POCER. Journal of Gastroenterology and Hepatology 2013;28:92-92.

39. De Cruz P, Kamm M, Hamilton A, et al. Smoking is the key risk factor that doubles the risk of postoperative recurrence of Crohn's disease despite preventive drug treatment. Results from the POCER study. Journal of Gastroenterology and Hepatology 2013;28:92-92.

40. De Cruz P, Kamm M, Hamilton A, et al. Strategic timing of anti-TNF therapy in postoperative Crohn's disease: Comparison of routine use immediately postoperatively with selective use after demonstrated recurrence at 6 month endoscopy. Results from POCER. United European Gastroenterology Journal 2013;1:A16.

41. De Cruz P, Kamm MA, Hamilton AL, et al. Efficacy of thiopurines and adalimumab in preventing Crohn's disease recurrence in high-risk patients - a POCER study analysis. Aliment Pharmacol Ther 2015;42:867-79.

42. Ewe K, Herfarth C, Malchow H. Surgical and internal medicine therapy study of the postoperative prevention of recurrence in Crohn's disease - completion of a partly randomized study. Verhandlungen der Deutschen Gesellscha1 fur Innere Medizin 1980;86:1327-37.

43. Ewe K. Effectiveness of Azulfidine/Salazopyrin in the postoperative prevention of recurrence in Crohn disease. Zeitschrift fur Gastroenterologie - Verhandlungsband 1981;19:41-4.

44. Ferrante M, Papamichael K, Duricova D, et al. Systematic versus Endoscopy-driven Treatment with Azathioprine to Prevent Postoperative Ileal Crohn's Disease Recurrence. J Crohns Colitis 2015;9:617-24.

45. Kamm MA, De Cruz P, Wright E, et al. Optimising post-operative Crohn's disease management: Best drug therapy alone versus endoscopic monitoring, disease evolution, and faecal calprotectin monitoring. The POCER study. Journal of Crohn's and Colitis 2014;8:S13.

46. Kennedy N, Ennis H, Gaya D, et al. Interobserver agreement in assessment of rutgeerts' score of endoscopic recurrence of ileal crohn–s disease. Gut 2015;64:A243.1-A243.

47. Liao NS, Ren JA, Fan CG, et al. Efficacy of polyglycosides of Tripterygium wilfordii in preventing postoperative recurrence of Crohn disease. Zhonghua Wei Chang Wai Ke Za Zhi 2009;12:167-9.

48. McLeod RS, Wolff BG, Steinhart AH, et al. Risk and significance of endoscopic/radiological evidence of recurrent Crohn's disease. Gastroenterology 1997;113:1823-7.

49. NCT00074542. An efficacy and safety study of Omega-3 free fatty acids (Epanova™) for the maintenance of symptomatic remission in subjects with Crohn's disease, 2003.

50. NCT01696942. Cimzia versus mesalamine for Crohn's recurrence, 2012.

51. NCT02247258. Azathioprine in the prevention of ileal Crohn'sdisease postoperative recurrence, 2014.

52. NCT02255370. Curcumin associated with thiopurin in the prevention of post-op recurrence in Crohn disease.

53. NCT02997059. Effect of fluconazole on the levels of ASCA after surgical resection for Crohn's disease.

54. Papamichael K, Archavlis E, Lariou C, Mantzaris GJ. Adalimumab for the prevention and/or treatment of post-operative recurrence of Crohn's disease: a prospective, two-year, single center, pilot study. J Crohns Colitis 2012;6:924-31.

55. Regueiro M, Baidoo L, Kip K, et al. Infliximab Maintenance Beyond One Year Prevents Postoperative Crohn's Disease Recurrence: Long-Term Follow-up From the Randomized Controlled Pilot Study. Gastroenterology 2013;144:S-173.

56. Regueiro M, Kip KE, Baidoo L, et al. Postoperative therapy with infliximab prevents long-term Crohn's disease recurrence. Clin Gastroenterol Hepatol 2014;12:1494-502 e1.

57. Ren J, Wu X, Liao N, et al. Prevention of postoperative recurrence of Crohn's disease: Tripterygium wilfordii polyglycoside versus mesalazine. J Int Med Res 2013;41:176-87.

58. Steinhart AH, O'Rourke K, Wolff BG, McLeod RS. Application of a stopping rule based on total treatment failures: the postoperative Crohn's disease trial. J Clin Epidemiol 1992;45:495-504.

59. Tao QS, Ren JA, Ji ZL, et al. Maintenance effect of polyglycosides of Tripterygium wilfordii on remission in postoperative Crohn disease. Zhonghua Wei Chang Wai Ke Za Zhi 2009;12:491-3.

60. Wright EK, De Cruz PP, Kamm MA, et al. DOP086 Intestinal resection in Crohn's disease is associated with significant and durable improvement in health related quality of life although to a lesser extent in women and smokers. Results from the POCER study. Journal of Crohn's and Colitis 2014;8:S56-S57.

61. Yamamoto T, Umegae S, Matsumoto K. Impact of infliximab therapy after early endoscopic recurrence following ileocolonic resection of Crohn's disease: a prospective pilot study. Inflamm Bowel Dis 2009;15:1460-6.

62. Zhu W, Li Y, Gong J, et al. Tripterygium wilfordii Hook. f. versus azathioprine for prevention of postoperative recurrence in patients with Crohn's disease: a randomized clinical trial. Dig Liver Dis 2015;47:14-9.
